# Multi-Step Synthesis of Chimeric Nutlin–DCA Compounds Targeting Dual Pathways for Treatment of Cancer

**DOI:** 10.3390/molecules30193908

**Published:** 2025-09-28

**Authors:** Davide Illuminati, Rebecca Foschi, Paolo Marchetti, Vinicio Zanirato, Anna Fantinati, Claudio Trapella, Rebecca Voltan, Virginia Cristofori

**Affiliations:** 1 Department of Life Sciences, University of Modena and Reggio Emilia, Via G. Campi 213/d, 41125 Modena, Italy; davide.illuminati@unife.it; 2 Department of Environmental and Prevention Sciences, University of Ferrara, Via Luigi Borsari, 46, 44121 Ferrara, Italy; rebecca.foschi@unife.it (R.F.); anna.fantinati@unife.it (A.F.); rebecca.voltan@unife.it (R.V.); 3 Department of Chemical, Pharmaceutical and Agricultural Sciences, University of Ferrara, Via Fossato di Mortara, 17, 44121 Ferrara, Italy; paolo.marchetti@unife.it (P.M.); vinicio.zanirato@unife.it (V.Z.); claudio.trapella@unife.it (C.T.); 4 Laboratorio per le Tecnologie delle Terapie Avanzate (LTTA), Via Fossato di Mortara, 70, 44121 Ferrara, Italy

**Keywords:** cancer research, Nutlin, DCA, chimeric compounds, chemoenzymatic synthesis

## Abstract

Chimeric compounds represent a promising strategy in cancer therapy by simultaneously targeting multiple pathways responsible for tumour growth and survival. Their structure comprises two or more pharmacophores connected through suitable chemical linker. These dual or multi-functional drugs can interact with several biological targets for a more pronounced pharmacological effect. In order to identify new multi-targeting agents with anticancer efficacy, we designed and synthesised a series of novel multi-functional molecules by covalently linking antitumor compounds dichloroacetate (DCA) and Nutlin-3a. The design was aimed at addressing two critical events in cancer: (1) the Warburg effect and (2) the dysregulations of protein p53 pathway, both of which are directly linked to the predominant survival and aggressive proliferation of malignant cells. DCA reactivate oxidative phosphorylation by inhibiting mitochondria pyruvate dehydrogenase kinase (PDK), thereby unlocking the Warburg metabolism of cancer cells and its antiapoptosis state. Concurrently, Nutlin-3a restores the protective function of the “genome guardian” p53 protein, by blocking its antagonist oncoprotein E3 ligase MDM2. Chimeric compounds were obtained using a chemoenzymatic multi-step procedure that included a key lipase-catalysed asymmetric reaction. Biological evaluation of the synthesised Nutlin-DCA chimeras in a panel of three cancer cell lines demonstrated promising results in vitro. Specifically, compounds *rac*-**19a**, *rac*-**19b**, *rac*-**20a**, *rac*-**20b** and enantioenriched **20a** caused a statistically significant reduction in cell viability at micromolar concentrations. These findings suggest that targeting both the Warburg effect and the p53 pathway with a single molecule is a viable approach for future cancer therapeutic development.

## 1. Introduction

A major challenge in cancer research is selectively inducing death exclusively in damaged cells, leaving normal cells unharmed. One approach to address this issue is the treatment of different pathways that integrate multiple signals involved in tumour growth. In recent years, a new concept of dual- or multi-functional drug has taken hold in chemical biology and can be applied accordingly [1,2]. This concept is based on the possibility to bridge, within the same chemical construct, through appropriate conjugation chemistry strategies, two entities with distinct biological activities. An organic spacer connecting the pharmacophores is typically used as a chemical linker. The result is a chimeric molecule with the ability to interact with different targets, ideally having related functions for more pronounced therapeutic effect. These systems are primarily used to treat multi-factorial diseases, where multiple receptors and pathways are simultaneously dysregulated. Indeed, due to its potential synergistic effect, such an approach is particularly valuable during the intertwined mechanistic nature of the onset and the development of cancer [1,2,3,4,5].

In order to design and build novel multi-functional ligands, in this work we focused on two critical pathways that concurrently contribute to tumour expansion. In particular, we aimed to tackle the predominant survival state of cancer cells caused by (1) the Warburg effect and (2) the dysregulations of protein p53 pathway. Our research group has developed expertise in the synthesis and derivatisation of two small-molecules that inhibit these phenomena: dichloroacetate-loaded (DCA) compounds [6] and enantioenriched Nutlin-3a [7,8].

The most common metabolic dysfunction observed in solid tumours is known as Warburg effect and it consists in the metabolic shift from oxidative phosphorylation to a frenetic aerobic glycolysis for a rapid ATP synthesis. This translates to an uncontrolled proliferative state and a strong resistance to programmed death (apoptosis). There is growing evidence that mitochondria-targeting is a powerful way to restore normal metabolism [9,10,11]. Dichloroacetic acid and in particular, dichloroacetate (DCA) has gained attention in this field as a potent and effective small-molecule that unlocks cancer cells from that antiapoptosis state. DCA indirectly activates the key switching enzyme, pyruvate dehydrogenase (PDH), via inhibition of pyruvate dehydrogenase kinase (PDK). PDH converts pyruvate to acetyl-CoA, promoting oxidative phosphorylation while leaving the mitochondria of normal cells largely unaffected. It was shown that DCA activity increases mitochondrial reactive oxygen species (ROS) and leads to tumour growth suppression both in vitro and in vivo [12,13,14]. However, due to its negatively ionised nature in physiological conditions, DCA itself needs to be chemically engineered for its clinical evaluation to overcame solubility problems, limited cellular uptake and bioavailability issues. For instance, in the trivalent Mito-DCA, DCA is carried by a lipophilic triphenylphosphonium cation [15,16]. We reported noncytotoxic ammonium salts and tertiary amines for efficient mitochondria DCA delivery [6].

The transcription factor protein p53, often referred to as “the genome guardian”, plays a crucial role in cell cycle regulation and maintaining DNA integrity. Its function as a tumour suppressor is well-known and it has been the focus of extensive research aimed at harnessing its potential for cancer therapy [17,18,19,20]. Studies using advanced animal models have demonstrated that activating p53 protective response—even in advanced tumours—can have therapeutic effects [21]. One promising strategy to restore wild-type p53, extensively explored as a potential cancer treatment, consists in targeting its antagonist, the MDM2 oncoprotein, with small molecule inhibitors [22,23,24,25,26,27,28,29]. In cancer, MDM2 overexpression or genetic amplification is the most common genomic alteration, which inactivates p53 through an auto-regulatory negative feedback loop. One class of inhibitors, 1,2,4,5-tetrasubstituted-4,5-*cis*-imidazolines, known as Nutlins, specifically disrupt the p53-MDM2 protein–protein interaction (PPI). These compounds have demonstrated antiproliferative activity and the ability to inhibit tumour growth [23,30]. Developed through high-throughput screening by Hoffmann-La Roche, the preclinical compound (−)-Nutlin-3, also called Nutlin-3a, is among the most extensively studied [31]. From seminal crystallographic studies (PDB ID 7C44) emerged that Nutlin-3a binds to the p53-binding pocket of MDM2 with high affinity, displacing p53 [23,32,33]. Nutlin-3a activity also influences mitochondrial function as it reduces the levels of dihydrolipoamide dehydrogenase/dihydrolipoamide acetyltransferase protein complexes, which in turn, leads to the disruption of the pyruvate dehydrogenase complex and inhibition of mitochondrial activity [34,35]. Numerous studies regarding the use of these heterocyclic chemotherapeutic drugs in basic cancer biology and clinical trials are pervasive in the oncology literature. Moreover, scaffold represents an excellent leading compound for ligand structure optimisation. In fact, Nutlins development has evolved from a simple synthetic target to a platform for more complex, multi-target therapies with enhanced antitumoral properties [22,28,36,37]. Several efforts have been initially focused on asymmetric syntheses and racemic resolution since there is a rather high eudysmic ratio between the levorotatory eutomer (i.e., more potent enantiomer) (−)-(4S,5R)-Nutlin-3 (Nutlin-3a) and distomer (i.e., less potent enantiomer) (+)-(4R,5S)-Nutlin-3 (Nutlin-3b) [7,38,39,40,41]. More recent advances have also aimed to make the synthesis of Nutlins more efficient and environmentally friendly, with the use of green chemistry techniques such as solvent-free reactions and catalytic processes. We described an enzymatic solvent-free process to enatioselectively provide an alkoxycarbonylated desymmetrised *meso*-diamine, a key Nutlin-3a precursor, with good enantiomeric excesses [8]. Other advances in the synthesis of Nutlins aimed to enhance their bioavailability, stability and selectivity, improving water solubility and reducing off-target effects, to address the challenges of clinical application. Several new approaches have emerged leading to interesting Nutlin derivatives that have been pursued in clinical trials [37,42]. Some strategies have also included the incorporation of hydrophilic groups, such as PEGylation and the development of nanoparticles delivery [36,43,44]. Finally, a significant area of focus has been the synthesis of Nutlin-based chimeric compounds that combine Nutlin’s MDM2-p53 inhibition with other pharmacophores targeting additional cancer-related pathways. For example, hybrid compounds that incorporate Nutlin with DNA-damaging agents or kinase inhibitors have shown promise in enhancing anticancer efficacy [3,4,5,44,45,46,47,48,49,50,51,52].

Moreover, it has been shown that combined treatment with DCA and Nutlin-3a resulted in significantly greater antileukemic activity with increased cytotoxicity compared to treatment with the single agents, showing a synergistic effect both in primary B-chronic lymphocytic leukemia (B-CLL) patient samples as well as in B leukemic cell lines [53].

Taking together these considerations, new chimeric compounds, derived from the connection of DCA and Nutlin through suitable biodegradable linkers, could simultaneously inhibit mitochondrial kinase PDK and prevent degradation of p53, to impede tumour development while minimising harm to normal cells. Therefore, the ultimate aim of this work is the selection of new multi-targeting drugs with enhanced antitumoral efficacy. This would also allow for a reduction in the otherwise prohibitive dosage, ultimately minimising adverse effects and increase prospective in clinical applications.

## 2. Discussion and Results

### 2.1. Chemistry

#### 2.1.1. Structural Modification of Original Molecule

**Figure 1:**
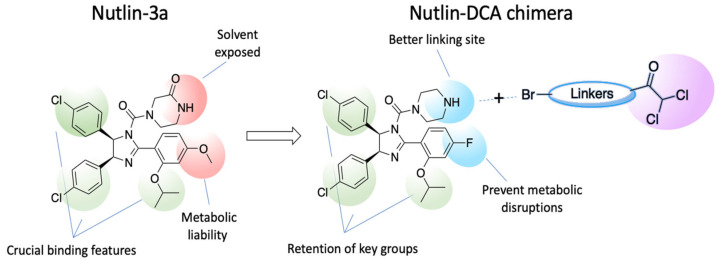
Structural modification of Nutlin-3a: Key modifications, highlighted with colours red and cyano, include (1) OMe group substitution with F atom for improved metabolic stability (2) oxo-piperazine substitution with piperazine for exploration of side chain effects while maintaining crucial MDM2-binding interactions. Unchanged moieties are highlighted in green.

To optimize Nutlin-3a structure and develop chimeric derivatives, we prioritised preserving key MDM2-binding elements while strategically modifying the structure to address metabolic stability and synthetic accessibility (Figure 1). Crystallographic analyses of Nutlin-MDM2 complexes revealed the critical binding interactions: the *para*-chlorophenyl groups at the C4 and C5 positions of the imidazoline scaffold efficiently mimic p53 aminoacidic residues Trp23 and Leu26, respectively, while the *ortho*-isopropoxy group at C2 occupies the Phe19 pocket [32]. These moieties, needed for protein recognition, were retained in our design. In vitro studies had shown that the *para*-methoxy group on aromatic ring attached at imidazoline C2 position is particularly susceptible to metabolic degradation, yielding a phenol as major metabolite. To mitigate this liability, we replaced this methoxy group with a metabolically more robust fluorine atom, enhancing stability and thus, improving the pharmacokinetic profile. This modification was also expected to fine-tune lipophilicity and electronic properties of the molecule without compromising MDM2 binding. Nutlin-3a features an oxo-piperazine appendage extending from the N1 position of the imidazoline that does not penetrate the binding cleft. This solvent-exposed moiety offers an ideal point of attachment for the linkers to the DCA pharmacophore. As demonstrated in prior structure-activity relationship (SAR) studies, modifications at this site, such as side chain introduction, do not significantly impact protein binding. For synthetic convenience in connecting the DCA moiety through chemical spacers, we replaced the original oxo-piperazine moiety with piperazine, enabling facile *N*-alkylation of the resulting secondary amine, a more synthetically tractable conjugation compared to oxo-piperazine *N*-derivatisation.

**Scheme 1:**
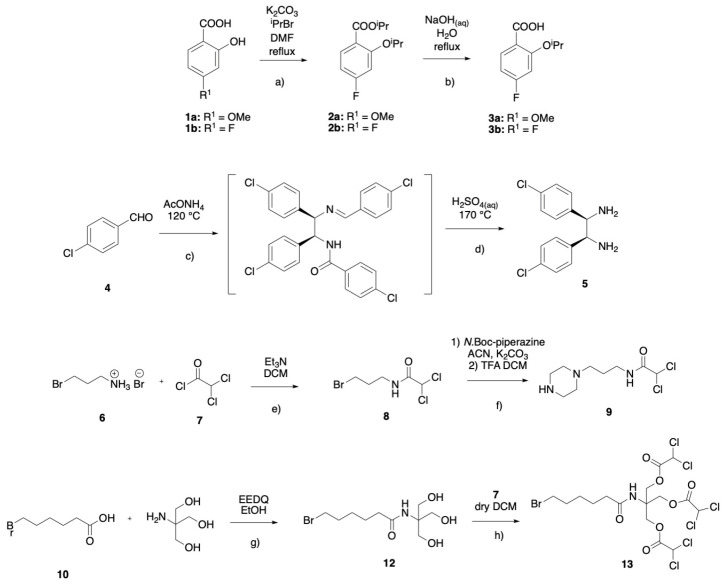
Synthesis of building blocks **3a–b**, **5** and DCA-linkers **9** and **13**. Reaction condition: (**a**) ^i^PrBr, K_2_CO_3_, DM, refluxF; (**b**) NaOH_(aq)_, reflux; (**c**) AcONH_4_, neat, 120 °C; (**d**) H_2_SO_4(aq)_, 170 °C; (**e**) Et_3_N, DCM; (**f**) (**1**) N-Boc-piperazine, K_2_CO_3_, ACN, r.t.; (**2**) TFA, DCM, r.t.; (**g**) EEDQ, EtOH; (**h**) **7**, dry DCM.

The choice for an appropriate linker can be critical in chimera design, for this reason, we installed two diverse linkers having different lengths and reactive groups for conjugation, to investigate their impact on the properties and activity of the resulting chimeric compounds. Both linkers feature a terminal bromine atom for the nucleophilic substitution onto the Nutlin piperazine (Scheme 1). One linker, compound **6**, comprises a propane chain with a primary amine group for coupling to DCA chloride via amide bond formation. The other linker, spacer **12**, has a slightly longer carbon chain terminating in a tris(hydroxymethyl)amide that enables the attachment of up to three DCA molecules through ester bonds, offering the possibility for a multivalent conjugation.

#### 2.1.2. Synthesis of Chimeric Compounds

The Nutlin structural scaffold was prepared via both asymmetric and racemic approaches, incorporating substantial improvements to previously reported methods [8,39]. Full characterisation of synthesised molecules and intermediates are available in Supporting Information.

Building blocks used to construct imidazoline ring, namely decorated salicylic acids **3a–b** [41], *meso*-diamine **5** [54,55], as well as the pharmacophore spacers-DCA **9** and **13** were synthesised through standard procedures as outlined in Scheme 1 [6,15].

**Scheme 2:**
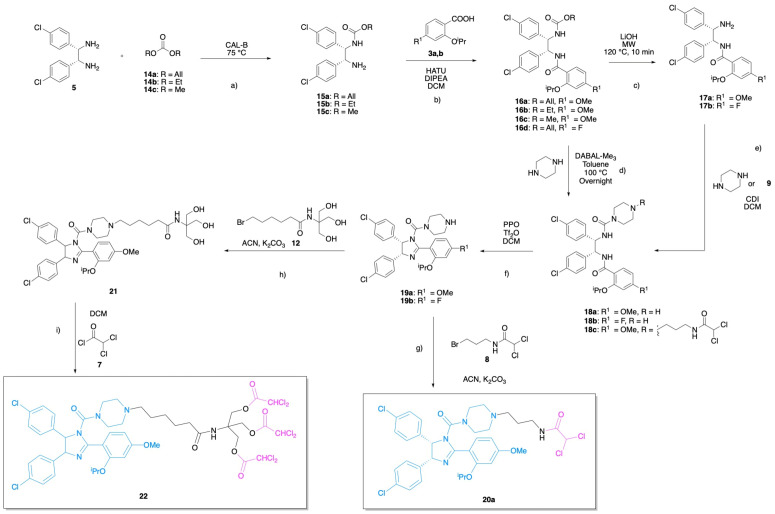
Overall enantioselective synthesis of Nutlin–DCA chimera compounds **22** and **20**. (**a**) dialkyl carbonate **14a–c**, lipase from Candida Antarctica type B, 75 °C; (**b**) HATU, DIPEA, **3a–b**, DCM, r.t;. (**c**) LiOH 2M, H_2_O:MeOH:THF, 120 °C, MW; (**d**) DABAL-Me_3_, Toluene, 100 °C; (**e**) CDI, piperazine or **9**, DCM, r.t.; (**f**) PPO, Tf_2_O, DCM, r.t.; (**g**) **8**, K_2_CO_3_, ACN, reflux; (**h**) **12**, K_2_CO_3_, ACN reflux; (**i**) **7**, DCM.

The overall enantioselective synthesis of the target molecules is shown in Scheme 2. The key asymmetric transformation involves the desymmetrisation of prochiral *meso*-diamine **5** via green biocatalytic process, yielding Alloc-protected compounds **15a–c**. Specifically, supported lipase from *Candida Antarctica* type B (CAL-B) was employed as a biological catalyst, affording enantioenriched carbamates **15a–c** with good enantiomeric excess [8]. Subsequent amide coupling with substituted benzoic acid **3a–b** was accomplished in the presence of coupling agent HATU and DIPEA, furnishing compound **16a–d**. Standard methods for removal of allyl, ethyl, or methyl carbamate protecting groups, e.g., acid catalysis, Pd-catalysed transfer, proved ineffective or led to poor yields. Nevertheless, we optimised a high-temperature hydrolysis with LiOH under microwave (MW) irradiation to rapidly and efficiently afford the corresponding mono-amine **17a–b**. The piperazine ring was then introduced at amine nitrogen via urea formation using carbonyl diimidazole (CDI) as a carbonyl synthon [40]. Alternatively, our more direct approach based on one-step protocol to the unsymmetrical piperazine urea **18a–b** was conveniently achieved using air-stable reagent DABAL-Me_3_ in toluene at 100 °C, starting from corresponding *N*-Alloc amine **15a–c**. This streamlined procedure reduced the number of steps, ultimately enhancing the overall synthesis. Imidazoline ring closure was catalysed by Hendrickson ‘POP’ dehydrating agent [56], prepared in situ from PPO and Tf_2_O. However, the cyclisation of compound **18c**, applying the same ‘POP’ conditions did not occur, due to somehow different reactivity of this DCA-bearing substrate.

The conjugation of the spacer and DCA moiety was achieved in the final stages of the synthesis. Specifically, compound **20** was prepared via a convergent synthetic route involving the initial attachment of DCA to the spacer, followed by linkage to the Nutlin core. In contrast, the preparation of more complex trivalent compound **22** preferentially involved introducing DCA as the last step to the trihydroxy precursor **21**, as this strategy resulted in better yields.

**Scheme 3:**
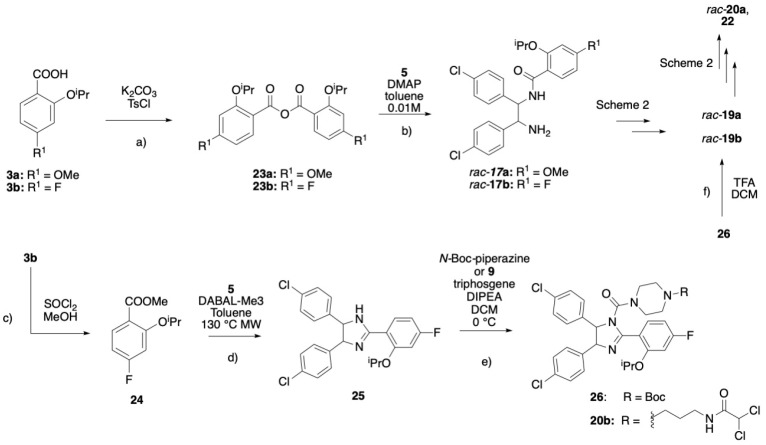
Key steps for racemic synthesis. (**a**) K_2_CO_3_, TsCl, r.t.; (**b**) **5**, DMAP, toluene, 0.01 M (**c**) SOCl_2_, MeOH; (**d**) **5**, DABAL-Me_3_, toluene, 130 °C, MW; (**e**) *N*-Boc-piperazine or **9**, triphosgene, DIPEA, DCM 0 °C; (**f**) TFA, DCM.

Racemic chimeras were prepared with almost identical synthetic paths, only excluding the enzymatic alkoxycarbonylation step. In this case, the amide coupling of the vicinal diamine **5** with aromatic acid **3a–b** to obtain monoamide *rac*-**17a–b** mostly resulted in unwanted dialkylation, leading to unwanted *meso*-diamide as the main product. This side reaction was mitigated by performing the coupling with slow addition of less reactive acylating agent, namely the corresponding anhydride **23a–b**, to a low concentration solution of **5** in the presence of DMAP as a catalyst (Scheme 3). The following steps, namely, CDI coupling and Hendrickson’s ring closure, to afford *rac*-**19a–b** were the same as those described for enantiomeric synthesis at Scheme 2.

Alongside this route, we also carried out an alternative strategy for fluorine chimeric derivative *rac*-**20b** that involved direct imidazoline ring closure by reacting the corresponding aromatic methyl ester **24** under microwave irradiation, with DABAL-Me_3_ as a coupling agent. This afforded the achiral compound **25**, which was then functionalised with *N*-Boc-piperazine or DCA-bearing compound **9** through triphosgene-mediated carbonylation (Scheme 3). Piperazine was protected as a Boc anhydride to prevent its carbonylation and subsequent side reactions, which would significantly reduce the yield; Boc removal was performed in standard acidic condition with trifluoroacetic acid (TFA).

Finally, as previously depicted in Scheme 2, alkylation of piperazine in *rac*-**19a** with bromide compound **8** or compound **12**, in presence of K_2_CO_3_ led to racemic mixtures of target molecules, *rac*-**20a** or **22** after DCA conjugation, respectively.

**Scheme 4:**
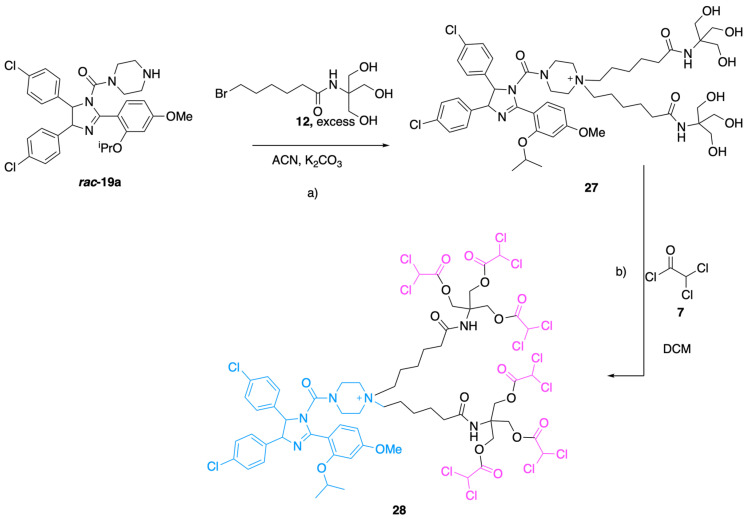
Overalkylation and subsequent acetylation of intermediate **19a** to obtain hexa-DCA derivative **28**. Reaction conditions: (**a**) excess of **12**, K_2_CO_3_, ACN; (**b**) **7**, DCM.

We also found that with excess of alkylating agent **12**, the secondary amine in piperazine ring undergoes overalkylation, leading to quaternary ammonium species **27**. This unexpected side product was in turn purified and kept for subsequent acetylation with DCA chloride **7** to afford hexavalent derivative **28** (Scheme 4), which was also evaluated in biological tests.

### 2.2. Biological Evaluation

#### Compounds *rac*-**19a,b**, **20a**, *rac*-**20a,b** Induce Cytotoxicity in Cancer Cells

**Figure 2:**
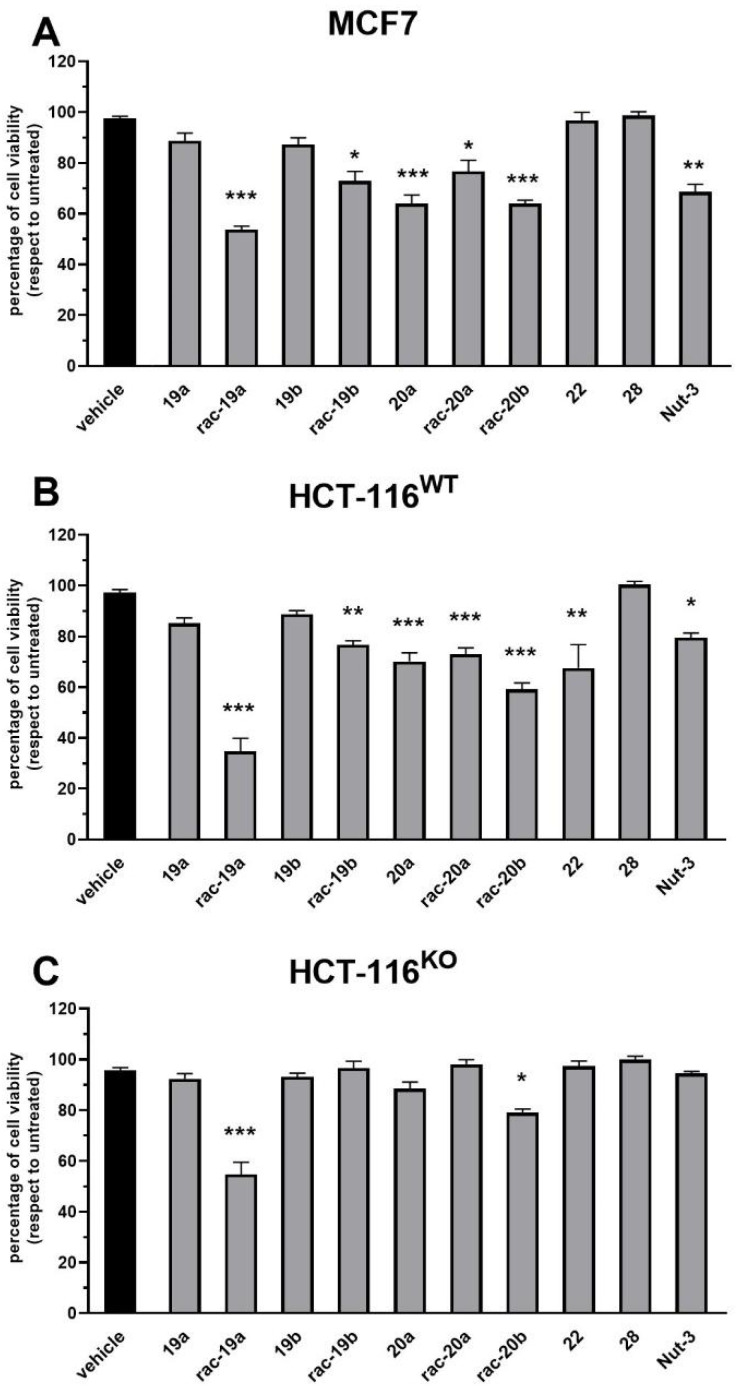
Cytotoxicity effects of the new synthetic compounds on MCF7 (**A**) and HCT-116^WT^ (**B**) cell lines, both expressing p53 wild-type and on the p53 knock-out HCT-116^KO^ cell line (**C**). Cells were treated with the new synthetic compounds (10 µM) and commercial Nutlin-3 (Nut-3, 10 µM) for 24 h and assessed with MTT for cytotoxic evaluation. Untreated cultures and cultures exposed to vehicle were run as negative controls. Results are reported as mean ± SEM percentage of cell viability with respect to untreated cultures set to 100 percent, from at least three independent experiments. Statistical analyses were performed using the Kruskal–Wallis method. * *p* < 0.05; ** *p* < 0.01; *** *p* < 0.005 with respect to vehicle.

Racemic and enantiomer Nutlin’s, the commercially available racemic Nutlin-3 and a series of chimeric Nutlin-DCA compounds were evaluated for biological activity in two human cancer cell lines known for their sensitivity to Nutlin-3 due to the expression of wild-type p53 protein: MCF7 and HCT-116^WT^. As shown in Figure 2A,B treatment with *rac*-**19a**, *rac*-**19b**, enantioenriched **20a**, *rac*-**20a** and *rac*-**20b** and commercial Nutlin-3 led to a statistically significant reduction in cell viability compared to vehicle (*p* < 0.05) in both cell lines. Notably, HCT-116^WT^ cells displayed increased sensitivity to compound **22**, which also significantly reduced cell viability. These active compounds demonstrated comparable or superior efficacy in reducing cell viability relative to the commercial Nutlin-3. To assess whether these effects were dependent on functional p53, the compounds were also tested in HCT-116^KO^ cells that completely miss the expression of p53 (Figure 2C). In this model, which serves as a negative control for p53 activity, many compounds did not significantly affect cell viability, indicating the dependence on p53 for the biological effect of the active molecules. Exceptions are the compounds *rac*-**19a** and *rac*-**20b**, both of which induced a significant decrease in cell viability (*p* < 0.05) also in this cell line, suggesting a potential off-target cytotoxicity independent by p53. For clarity, Figure 2 shows the cellular response to the 10 µM concentration, while the response to the full range of concentrations of every molecule is displayed in the Supplementary Information.

It is worth noting that compound **28**, bearing six equivalents of DCA moiety and that was afforded after overalkylation of piperazine derivative, unfortunately displayed poor activity. This result can be compared with slightly higher activity observed with its homologue **22**, especially in HCT-116^WT^. Since compounds **22** and **28** display almost a similar structure, this result demonstrates an insight into the structure–activity relationship to evaluate the impact of overalkylation.

## 3. Materials and Methods

### 3.1. Chemistry

All the NMR spectra were elaborated using Mestre MNova 6.0.2 software. http://www.mestrelab.com (accessed on 1 August 2025) and FID data are available on request. Analytical thin layer chromatography (TLC) was performed on silica gel Macherey-Nagel Polygram SIL/UV 254 of 0.25 mm and visualisation was achieved using UV light (254) and potassium permanganate (KMnO_4_) 2% in water. Flash column chromatography was undertaken on silica gel Merck 60–200 mesh using chromatography (Isolera, Biotage, Uppsala, Sweden). Products were dried using anhydrous sodium sulphate (Carlo Erba, Milan, Italy). Proton nuclear magnetic resonance (^1^H NMR) and carbon nuclear magnetic resonance (^13^C NMR) were recorded using Nuclear Magnetic Resonance Spectrometer (VARIAN 400 MHz, Varian, Palo Alto, CA, USA). All spectra were recorded using as solvent CDCl_3_, unless otherwise specified. Chemical shifts (*δ*) were quoted in ppm relative to residual solvent and coupling constants (*J*) were quoted in hertz (Hz). Multiplicity was reported with the following abbreviations: s = singlet; d = doublet; t = triplet; q = quartet; m = multiplet, bs = broad signal. Molecular weights were measured with a mass spectrometer ESI MICROMASS ZMD 2000 (Waters, Manchester, UK) and high-resolution spectra with an Agilent ESI-Q- TOF LC/MS 6520 system (Agilent Technologies, Santa Clara, CA, USA). Solvents and chemicals used for TLC, chromatographic purification, crystallisations and reactions were reported with the following abbreviations: Et_2_O for diethyl ether, THF for tetrahydrofuran, AcOEt for ethyl acetate, DCM for methylene chloride, ACN for acetonitrile, TFA for trifluoroacetic acid. Preparative HPLC purifications were performed using Phenomenex Jupiter C_18_ column, 15 µm, 300 Å, 250 × 30 mm (L × ID), flow rate 20 mL/min. The biocatalysts *Candida antarctica* lipase type B immobilised on acrylic polymeric support (Novozyme 435^®^, CAL-B, 9000 PLU/g propyl laurate unit) was provided by Novozymes (Lyngby, Denmark).

#### 3.1.1. Isopropyl 4-Fluoro-2-isopropoxybenzoate (**2b**)

In a 100 mL round-bottom flask equipped with a magnetic stirrer and a reflux condenser, 4.0 g (26 mmol) of *p*-fluorosalicylic acid **1b** and 14.2 g (102 mmol, 4 equivalents) of K_2_CO_3_ were dissolved in 20 mL of DMF. After 15 min, 9.5 mL (102 mmol, 4 equivalents) of 2-bromopropane were added. The mixture was heated in an oil bath under reflux and stirred for 3.5 h, then it was cooled to room temperature and the solvent was removed to dry using a rotary evaporator. The residue was dissolved in AcOEt and filtered through nylon discs filter to remove K_2_CO_3_. The filtrate was washed with NaHCO_3_ three times. The organic phase was dried with anhydrous Na_2_SO_4_, filtered through a cotton plug and concentrated under reduced pressure. 4.1 g (17 mmol, 66% yield) of desired product **2b**, was obtained as a yellow solid. R_f_ (solvent system: AcOEt/Petroleum ether 7:3) = 0.95. ESI [M + H]^+^_calc_ = 241.12, ESI [M + H]^+^_found_ = 241.22. ^1^H NMR (400 MHz, Chloroform-*d*) *δ* 7.8–7.7 (m, 1H), 6.7–6.5 (m, 2H), 5.2 (*p*, *J* = 6.2 Hz, 1H), 4.5 (hept, *J* = 6.1 Hz, 1H), 1.5–1.3 (m, 12H). ^13^C NMR (101 MHz, cdcl_3_) *δ* 166.9, 165.7, 164.5, 133.5, 133.4, 118.6, 107.2, 106.9, 102.6, 102.4, 71.9, 68.3, 22.1, 22.0.

#### 3.1.2. 4-Fluoro-2-isopropoxybenzoic Acid (**3b**)

In a 100 mL round-bottom flask equipped with a magnetic stirrer and a reflux condenser, 4.1 g (17 mmol) of ester **2b** were dissolved in 34 mL (68 mmol, 4 equivalents) of 2N aqueous solution of NaOH. The mixture was refluxed for 2.5 h, then the solvent was removed to dry using a rotary evaporator. The residue was resuspended in water and it was acidified with a 2M aqueous solution of HCl (pH = 2). The acidic aqueous phase was extracted with AcOEt three times. The organic phases were combined, dried with Na_2_SO_4_, filtered through a cotton plug and concentrated to dry under reduced pressure. A total of 3 g (15 mmol, 89% yield) of acid **3b**, was obtained as a pale yellow solid. R_f_ (solvent system: AcOEt/Petroleum ether 3:7) = 0.34. ESI [M + H]^+^_calc_ = 199.08, ESI [M + H]^+^_found_ = 199.2. ^1^H NMR (400 MHz, Chloroform-*d*) *δ* 8.2 (ddt, *J* = 8.9, 6.9, 0.4 Hz, 1H), 6.8 (tdd, *J* = 7.6, 2.3, 0.7 Hz, 1H), 6.8–6.7 (m, 1H), 4.8 (hept, *J* = 6.1, 0.6 Hz, 1H), 1.5 (dd, *J* = 6.1, 0.7 Hz, 6H). ^13^C NMR (101 MHz, cdcl_3_) *δ* 164.6, 136.2, 136.1, 109.8, 109.6, 102.1, 101.9, 74.8, 22.0. ^19^F NMR (376 MHz, cdcl_3_) *δ* -101.3.

#### 3.1.3. N-(3-Bromopropyl)-2,2-dichloroacetamide (**8**)

In a 100 mL round-bottom flask, with magnetic stirring immerse an ice bath, 1 g (4.6 mmol) of 3-bromopropylamine hydrobromide **6** was dissolved in freshly distilled DCM (40 mL). Then, 0.5 mL (5 mmol; 1.25 equivalents) of 2,2-dichloroacetyl chloride **7** and 1.9 mL of triethylamine (TEA) (13 mmol; 3 equivalents) were added dropwise at 0 °C. After 3 h, the reaction was quenched with 2 M HCl aqueous solution, transferred to a separatory funnel and extracted 3 times with DCM. The organic phases were combined and dried over Na_2_SO_4_, filtered with a funnel on cotton and brought to dry with a rotary evaporator to give 1.1 g of product N-(3-bromopropyl)-2,2-dichloroacetamide **8** (3.8 mmol; 97%), an orange solid. ESI [M + H]^+^_calc_ = 247.92, [M + H]^+^_found_ = 249.64. ^1^H NMR (400 MHz, Chloroform-*d*) *δ* 6.70 (bs, 1H, NH), 5.92 (s, 1H), 3.57–3.49 (m, 2H), 3.45 (t, *J* = 6.4 Hz, 2H), 2.20–2.13 (m, 2H). ^13^C NMR (101 MHz, cdcl_3_) *δ* 164.6, 66.5, 39.1, 31.6, 30.4.

#### 3.1.4. *tert*-Butyl 4-(3-(2,2-Dichloroacetamido)propyl)piperazine-1-carboxylate, ***N*-Boc-9**

In a 100 mL flask equipped with a magnetic stirrer, 290 mg (0.83 mmol, 1.5 equivalents) of **8** and 155.45 mg (0.83 mmol, 1 equivalent) of N-Boc-piperazine were dissolved in 10 mL of ACN. The mixture was stirred for 1 h. Then, 400 mg (2.9 mmol, 4 equivalents) of K_2_CO_3_ were added and the mixture was stirred overnight. The next day, the reaction filtered through a Nylon membrane to remove excess K_2_CO_3_. The filtrate was concentrated under reduced pressure using a rotary evaporator and the residue was washed with water and AcOEt. The organic phase was dried over Na_2_SO_4_, filtered through a cotton plug and the solvent was removed to dry using a rotary evaporator. A total of 293 mg of crude product ***N*-Boc-9**, a brown solid, was obtained and used for the following step (Quantitative yield). ESI [M + H]^+^_calc_ = 354.13, [M + H]^+^_found_ = 354.23. ^1^H NMR (400 MHz, Chloroform-*d*) *δ* 8.5 (s, 1H), 5.9 (d, *J* = 0.5 Hz, 1H), 3.5 (dq, *J* = 12.0, 6.8, 5.9 Hz, 4H), 2.6–2.5 (m, 2H), 1.8–1.7 (m, 2H), 1.4 (d, *J* = 1.0 Hz, 9H). ^13^C NMR (101 MHz, cdcl_3_) *δ* 164.4, 154.6, 79.8, 66.7, 58.3, 53.2, 44.9, 41.2, 28.4, 28.3, 23.7.

#### 3.1.5. 2,2-Dichloro-N-(3-(piperazin-1-Yl)propyl)cetamide (**9**)

In a 100 mL flask equipped with a magnetic stirrer, 293 mg (0.83 mmol, 1 equivalent) of compound ***N*-Boc-9** was dissolved in 20 mL of DCM. Then, 635 µL (8.3 mmol, 10 equivalents) of TFA were added. The mixture was stirred overnight. The next day, the reaction was quenched and the solvent was removed to dry using a rotary evaporator. The residue was dissolved in a solution of NaHCO_3_ until a pH of 9 was reached. The product was extracted with AcOEt three times, the organic phases were combined and dried over Na_2_SO_4_, filtered through a cotton plug and concentrated under reduced pressure. Eventually, 150 mg (0.60 mmol) of product **9**, a yellow solid, were obtained (71% yield). ESI [M + H]^+^_calc_ = 254.08, [M + H]^+^_found_ = 254.11. ^1^H NMR (400 MHz, Chloroform-*d*) *δ* 8.6 (s, ^1^H), 5.9 (s, 1H), 3.5–3.3 (m, 2H), 3.0 (t, *J* = 4.9 Hz, 4H), 2.7–2.4 (m, 8H), 1.8–1.6 (m, 2H). ^13^C NMR (101 MHz, cdcl_3_) *δ* 164.5, 66.9, 58.9, 54.3, 45.7, 41.5, 29.8, 23.6.

#### 3.1.6. 6-Bromo-N-(1,3-dihydroxy-2-(hydroxymethyl)propan-2-Yl)hexanamide (**12**)

In a 100 mL round-bottom flask equipped with a magnetic stirrer, 1 g (5.1 mmol, 1 equivalent) of 6-bromohexanoic acid **10** and 1.52 g (6.15 mmol, 1.2 equivalents) of EEDQ were dissolve in ethanol (50 mL). After stirring the mixture for 20 min, a solution of 0.62 g (5.13 mmol, 1:1 equivalents) of tris(hydroxymethyl)aminomethane **11** in 40 mL of EtOH was added with a dropping funnel and the reaction was refluxed overnight. After the starting material was no longer detectable by TLC, the solvent was evaporated using a rotary evaporator. The product was purified from the crude mixture by gradient elution flash chromatography (2–15% DCM/MeOH) with dry loading on silica, obtaining 1 g of hexanamide **12** (3.3 mmol, yield 66%) as a colourless oil. TLC: (CH_2_Cl_2_/MeOH 9:1) R_f_ = 0,45. ESI [M + H]^+^_calc_ = 298.06, ESI [M + H]^+^_found_ = 298,18. ^1^H NMR (400 MHz, Methanol-*d*_4_) *δ* 5.5–5.4 (m, 1H), 3.7 (t, *J* = 2.9 Hz, 6H), 3.5–3.3 (m, 2H), 3.3–3.2 (m, 2H), 2.2 (t, *J* = 7.6 Hz, 2H), 1.9–1.8 (m, 2H), 1.6 (*p*, *J* = 7.5 Hz, 2H), 1.4 (dq, *J* = 10.8, 7.4, 7.0 Hz, 2H). ^13^C NMR (101 MHz, cd_3_od) *δ* 176.9, 63.4, 62.6, 54.7, 37.2, 34.1, 33.5, 28.6, 25.9.

#### 3.1.7. 2-(6-Bromohexanamido)-2-((1,2-dichloro-2-oxoethoxy)methyl)propane-1,3-diyl bis(2,2-Dichloroacetate) (**13**)

A previously dried 100 mL round-bottom flask was filled with 900 mg (3 mmol, 1 equivalent) of compound **12** and 50 mL of freshly distilled DCM. The suspension was gently heated until complete dissolution; then, 870 µL (9.05 mmol, 3 equivalents) of dichloroacetic acid chloride **7** was added to the solution. The reaction was stirred at r.t. overnight then the solvent was evaporated under reduced pressure. The product was purified from the crude by gradient concentration chromatography (10–40% petroleum ether/AcOEt) on silica gel. Finally, the fractions were collected to afford 267 mg (0.4 mmol, 13% yield) of product **13**. TLC: (ethyl acetate/petroleum ether 2:8) R_f_ = 0,4. ESI [M + H]^+^_calc_ = 627.86, ESI [M + H]^+^_found_ = 630.01. ^1^H NMR (400 MHz, Chloroform-*d*) *δ* 6.01 (d, *J* = 0.6 Hz, 3H), 4.68 (d, *J* = 2.0 Hz, 6H), 3.40 (td, *J* = 6.6, 1.7 Hz, 2H), 2.22 (td, *J* = 7.5, 1.4 Hz, 2H), 1.94–1.79 (m, 2H), 1.62 (*p*, *J* = 7.3 Hz, 2H), 1.54–1.39 (m, 2H). ^13^C NMR (101 MHz, cdcl_3_) *δ* 174.1, 163.9, 64.7, 63.9, 58.5, 36.7, 33.7, 32.3, 27.6, 24.5.

#### 3.1.8. General Enantioselective Biocatalytic Reaction [8]

A solution of *meso*-diamine **5** (200 mg, 0.7 mmol) in dialkyl carbonate **14a–c** was placed in an Erlenmeyer flask, under inert atmosphere and 2 g of immobilised CAL-B was added. The mixture was gently shaken at 70 °C in orbital shaking for 2 days. The mixture was cooled to room temperature and the reaction was finished by filtering off the enzyme which was rinsed twice with DCM. The solvent was evaporated under reduced pressure and the enantioenriched monocarbamate product **15a–c** was purified by silica gel column chromatography.

##### Allyl 2-Amino-1,2,-bis(4-chlorophenyl)ethyl)carbamate (**15a**)

A 65% yield. ESI [M + H]^+^_calc_ = 365.08, ESI [M + H]^+^_found_ = 365.1. TLC R_f_ (solvent system: AcOEt/Petroleum ether 1:1) = 0.28. Analytical Chiral HPLC Method A (Chiralpack-ID 250 mm × 4.6 mm, 5 μm. % MP (Hex:IPA) = 90:10. Flow = 1.5 mL/min. UV: 254 nm): t_R_ [(1S,2R)-**15a**] = 11.43 min (major ent in enzymatic reaction), t_R_ [(1R,2S)-**15a**] = 13.47 min (minor ent in enzymatic reaction). Method B (Whelk-01 SS 100 mm × 4.6 mm, 1.8 μm. % MP (Hex:IPA) = 80:20). Flow 1 mL/min): t_R_ [(1S,2R)-**15a**] = 4.68 min (major ent in enzymatic reaction), t_R_ [(1R,2S)-**15a**] = 12.00 min (minor ent in enzymatic reaction). 89% ee%. ^1^H NMR (400 MHz, Methanol-d4) *δ* 7.40–7.25 (m, 8H), 5.77 (ddt, J = 16.2, 10.6, 5.4 Hz, 1H), 5.16–5.02 (m, 2H), 4.77 (d, J = 8.5 Hz, 1H), 4.36 (qdt, J = 13.5, 5.2, 1.6 Hz, 2H), 4.10 (d, *J* = 8.6 Hz, 1H). ^13^C NMR (101 MHz, CD_3_OD) *δ* 134.2, 130.5, 130.3, 129.7, 129.4, 117.3, 66.3, 62.1, 60.5. [α]$\begin{array}{c} 20\\ D \end{array}$ = +12.5 (*c* 0.085, MeOH).

##### Ethyl 2-Amino-1,2,-bis(4-chlorophenyl)ethyl)carbamate (**15b**)

A 30% yield. ESI [M + H]^+^_calc_ = 352.08, ESI [M + H]^+^_found_ = 353.1. TLC R_f_ (solvent system: AcOEt/Petroleum ether 1:1) = 0.27. Analytical chiral HPLC: (Chiralpack-ID 250 mm × 4.6 mm, 5 μm. % MP (Hex:IPA) = 95:5. Flow = 1.5 mL/min. UV: 254 nm): t_R_ [(1S,2R)-**15b**] = 6.24 min (major ent) in enzymatic reaction, t_R_ [(1R,2S)- **15b**] = 9.12 min (minor ent in enzymatic reaction). 78% ee%. ^1^H NMR (400 MHz, DMSO-*d*6) *δ* 7.62 (d, *J* = 9.3 Hz, 1H), 7.43–7.24 (m, 8H), 4.54 (t, *J* = 8.9 Hz, 1H), 3.98 (d, *J* = 8.4 Hz, 1H), 3.79 (*p*, *J* = 6.7 Hz, 2H), 1.02 (t, *J* = 7.1 Hz, 3H). ^13^C NMR (101 MHz, dmso) *δ* 219.5, 197.7, 194.3, 155.3, 143.2, 140.3, 131.5, 131.1, 129.7, 129.2, 127.8, 127.6, 60.6, 59.6, 58.8, 14.4. [α]$\begin{array}{c} 20\\ D \end{array}$ = +14.9 (*c* 0.085, MeOH).

##### Methyl 2-Amino-1,2,-bis(4-chlorophenyl)ethyl)carbamate (**15c**)

A 95% yield. ESI [M + H]^+^_calc_ = 339.1, ESI [M + H]^+^_found_ = 339.07. TLC R_f_ (solvent system: AcOEt/Petroleum ether 1:1) = 0.3. Analytical chiral HPLC Method A: (Chiralpack-ID 250 mm × 4.6 mm, 5 μm. % MP (Hex:IPA) = 95:5. Flowrate = 1.5 mL/min. UV: 254 nm): t_R_ [(1S,2R)-**15c**] = 7.95 min, t_R_ [(1R,2S)-**15c**] = 10.19 min. Method B: (Whelk-01 SS 100 × 4.6 mm, 1.8 μm. % MP (Hex:IPA) = 80:20. Flowrate = 1 mL/min. UV: 228 nm): t_R_ [(1S,2R)-**15c**] = 4.88 min (major ent in enzymatic reaction), t_R_ [(1R,2S)- **15c**] = 14.80 min (minor ent in enzymatic reaction). 73% ee%. ^1^H NMR (400 MHz, Chloroform-*d*) *δ* 7.3–7.2 (m, 4H), 7.0 (m, 2H), 7.0–6.9 (m, 2H), 5.7 (d, *J* = 8.2 Hz, 1H), 4.8 (s, 1H), 4.2 (d, *J* = 5.3 Hz, 1H), 3.6 (s, 3H), 1.6–1.4 (m, 2H). ^13^C NMR (101 MHz, cdcl_3_) *δ* 156.4, 140.2, 136.8, 133.7, 133.6, 128.9, 128.7, 128.5, 128.4, 59.3, 52.4. [α]$\begin{array}{c} 20\\ D \end{array}$ = +16.0 (*c* 0.085, MeOH).

#### 3.1.9. General Amidic Coupling Procedure

Benzoic acid derivatives **3a–b** (1.7 mmol) were dissolved in DCM, with 5.1 mmol HATU (3 equivalents). After 30 min, a solution of monocarbamate **15a–c** (1.4 mmol, 0.8 equivalents) in DCM (20 mL) and DIPEA (3.4 mmol, 2 equivalents) were added dropwise. The mixture was stirred overnight at room temperature. A white solid was filtered off through a Gooch filter and the liquid phase was transferred in a separatory funnel and washed three times with water and brine. Organic layers were combined, dried over Na_2_SO_4_ and concentrated (up to 30%) on a rotary evaporator then filtered to obtain the amido carbamate product **16a–d**.

##### Allyl (1,2-Bis(4-chlorophenyl)-2-(2-isopropoxy-4-methoxybenzamido)ethyl) Carbamate (**16a**)

Yellowish solid, 70% yield. ESI [M + H]^+^_calc_ = 557.16, ESI [M + H]^+^_found_ = 557.1. R_f_ (solvent system: AcOEt/Petroleum ether 1:1) = 0.5. ^1^H NMR (400 MHz, Chloroform-d) *δ* 8.4 (d, *J* = 8.4 Hz, 1H), 8.2 (d, *J* = 8.8 Hz, 1H), 7.3 (d, *J* = 8.4 Hz, 2H), 7.2 (d, *J* = 8.4 Hz, 2H), 7.0 (d, *J* = 8.1 Hz, 2H), 7.0–6.9 (m, 2H), 6.6 (dd, *J* = 8.8, 2.3 Hz, 1H), 6.5 (d, *J* = 2.3 Hz, 1H), 5.9 (dd, *J* = 11.0, 6.0 Hz, 1H), 5.8–5.7 (m, 1H), 5.2 (dd, *J* = 31.5, 13.7 Hz, 2H), 5.1 (dd, *J* = 7.4, 3.5 Hz, 1H), 4.7 (dp, *J* = 11.5, 6.0 Hz, 1H), 4.5 (td, *J* = 18.6, 15.5, 8.7 Hz, 2H), 3.8 (s, 3H), 1.3–1.2 (m, 9H). ^13^C NMR (101 MHz, cdcl_3_) *δ* 157.4, 136.6, 134.6, 133.7, 129.0, 128.8, 128.6, 128.5, 118.2, 105.5, 100.4, 71.6, 66.0, 60.5, 57.0, 55.7, 22.2.

##### Ethyl (1,2-Bis(4-chlorophenyl)-2-(2-isopropoxy-4-methoxybenzamido)ethyl) Carbamate (**16b**)

Yellowish solid, 87% yield. ESI [M + H]^+^_calc_ = 545.14, ESI [M + H]^+^_found_ = 545.8. R_f_ (solvent system: AcOEt/Petroleum ether 7:3) = 0.28. ^1^H NMR (400 MHz, DMSO-*d*_6_) *δ* 8.3 (d, *J* = 9.1 Hz, 1H), 7.8 (d, *J* = 9.4 Hz, 1H), 7.5–7.3 (m, 8H), 6.6–6.4 (m, 2H), 5.5 (t, *J* = 9.3 Hz, 1H), 5.0 (t, *J* = 9.7 Hz, 1H), 4.7 (*p*, *J* = 5.9 Hz, 1H), 3.8 (q, *J* = 7.0 Hz, 2H), 3.8 (d, *J* = 1.9 Hz, 3H), 2.1 (s, 2H), 1.2 (dt, *J* = 13.4, 6.5 Hz, 7H), 1.0 (t, *J* = 7.1 Hz, 3H). ^13^C NMR (101 MHz, dmso) *δ* 206.5, 163.7, 162.6, 156.5, 155.4, 139.7, 139.5, 132.1, 131.9, 131.7, 129.5, 128.0, 115.6, 105.9, 100.4, 71.1, 59.8, 57.7, 55.5, 55.3, 39.9, 30.7, 21.5, 21.4, 14.4.

##### Methyl (1,2-Bis(4-chlorophenyl)-2-(2-isopropoxy-4-methoxybenzamido)ethyl) Carbamate (**16c**)

Yellowish solid, 98% yield. ESI [M + H]^+^_calc_ = 531.14, ESI [M + H]^+^_found_ = 531.1. R_f_ (solvent system: AcOEt/Petroleum ether 1:1) = 0.28. ^1^H NMR (400 MHz, Chloroform-d) *δ* 8.5–8.4 (m, 1H), 8.2 (d, *J* = 8.8 Hz, 1H), 7.3–7.3 (m, 2H), 7.2–7.2 (m, 2H), 7.0 (d, *J* = 8.2 Hz, 2H), 7.0–6.9 (m, 2H), 6.6 (dd, *J* = 8.9, 2.3 Hz, 1H), 6.5–6.4 (m, 1H), 5.8 (d, J = 8.3 Hz, 1H), 5.1 (dd, *J* = 7.3, 3.4 Hz, 1H), 4.7 (*p*, *J* = 6.1 Hz, 1H), 3.8 (s, 3H), 2.8 (s, 3H), 1.3–1.2 (m, 6H). ^13^C NMR (101 MHz, cdcl_3_) *δ* 163.9, 157.4, 156.4, 134.6, 133.7, 129.1, 128.8, 128.6, 128.5, 114.1, 105.4, 100.4, 71.6, 60.5, 57.1, 55.7, 52.4, 38.7, 22.1, 21.7.

##### Allyl (1,2-Bis(4-chlorophenyl)-2-(4-fluoro-2-isopropoxybenzamido)ethyl)carbamate (**16d**)

Yellowish solid, 67% yield. ESI [M + H]^+^_calc_ = 545.14, ESI [M + H]^+^_found_ = 545.23. TLC R_f_ (solvent system: AcOEt/Petroleum ether 7:3) = 0.83. ^1^H NMR (400 MHz, Chloroform-*d*) *δ* 8.4 (d, *J* = 8.3 Hz, 1H), 8.2 (dd, *J* = 8.9, 7.1 Hz, 1H), 7.4–7.1 (m, 4H), 7.1–6.8 (m, 4H), 6.8 (ddd, *J* = 8.8, 7.5, 2.3 Hz, 1H), 6.7 (dd, *J* = 10.9, 2.3 Hz, 1H), 6.2 (d, *J* = 7.6 Hz, 1H), 6.0–5.7 (m, 2H), 5.3–5.0 (m, 3H), 4.7 (dp, *J* = 11.1, 5.9 Hz, 1H), 4.5 (d, *J* = 6.4 Hz, 2H), 1.3 (dd, *J* = 14.8, 6.0 Hz, 6H). ^13^C NMR (101 MHz, cdcl_3_) *δ* 167.1, 165.2, 164.6, 157.5, 157.4, 155.6, 136.5, 136.3, 135.0, 134.9, 134.1, 133.9, 132.7, 128.9, 128.9, 128.6, 118.3, 117.4, 108.5, 108.3, 101.4, 101.1, 72.4, 66.1, 60.2, 57.2, 22.1, 21.7. ^19^F NMR (376 MHz, cdcl_3_) *δ* -104.47.

#### 3.1.10. General Carbamate Removal Procedure

In a microwave vial, equipped with a magnetic stirrer, the enantiomerically enriched alkylcarbamate **16a–c** (0.4 mmol) were dissolved in 10 mL of a 2 M solution of LiOH (20 mmol), prepared in H_2_O, THF and MeOH (1:1:1). The mixture was reacted for 10 min at 120 °C. After this time, the mixture was filtered through a Gooch filter. The filtrate was diluted with AcOEt and washed three times with water. The organic phase was dried over anhydrous Na_2_SO_4_, filtered over cotton and evaporated using a rotary evaporator to give the enantiomerically enriched products **17a–b**.

##### N-(2-Amino-1,2-bis(4-chlorophenyl)ethyl)-2-isopropoxy-4-methoxybenzamide (**17a**)

*Y*ellowish oil, 92% yield. R_f_ (solvent system: AcOEt/Petroleum ether 7:3 = 0.43. ESI [M + H]^+^_calc_ = 473.4, ESI [M + H]^+^_found_ = 473.2. 1H NMR (400 MHz, Chloroform-*d*) *δ* 8.84 (d, *J* = 7.8 Hz, 1H), 8.11 (d, *J* = 8.8 Hz, 1H), 7.29–7.16 (m, 5H), 7.01 (dd, *J* = 8.5, 3.0 Hz, 4H), 6.55 (dd, *J* = 8.8, 2.3 Hz, 1H), 6.48 (d, *J* = 2.3 Hz, 1H), 5.47 (dd, *J* = 7.9, 4.6 Hz, 1H),4.74 (*p*, *J* = 6.1Hz, 1H), 4.43 (d, *J* = 4.6 Hz, 1H), 3.83 (d, *J* = 0.7 Hz, 3H), 1.41 (t, *J* = 5.8 Hz, 6H). 13C NMR (101 MHz, CDCl3) *δ* 165.1, 163.5, 157.4, 140.0, 136.8, 134.3, 133.4, 129.3, 128.6, 128.4, 114.9, 105.3, 100.5, 71.7, 59.2, 58.6, 55.7, 22.3, 22.2. 1H NMR (400 MHz, Methanol-*d*4) *δ* 7.92 (d, *J* = 9.4 Hz, 1H), 7.42 (m, 4H), 7.29 (d, *J* = 8.5 Hz, 2H), 7.22 (d, *J* = 8.5 Hz, 2H), 6.63 (dd, *J* = 4.7, 2.4 Hz, 2H), 5.83 (d, *J* = 5.5 Hz, 1H), 4.80 (dd, *J* = 8.7, 5.8 Hz, H), 3.84 (s, 3H), 1.29 (d, *J* = 6.1 Hz, 3H), 1.19 (d, *J* = 6.1 Hz, 3H). 13C NMR (101 MHz, CD3OD) *δ* 165.7, 137.5, 136.3, 135.5, 134.5, 130.7, 130.2, 130.1, 107.1, 101.4, 73.2, 59.9, 57.0, 56.1, 22.2, 22.2.

##### N-(2-Amino-1,2-bis(4-chlorophenyl)ethyl)-4-fluoro-2-isopropoxybenzamide (**17b**)

Light-yellow oil, 81% yield. R_f_ (solvent system: AcOEt/Petroleum ether 7:3 = 0.33. ESI [M + H]^+^_calc_ = 461.12, ESI [M + H]^+^_found_ = 461.26. ^1^H NMR (400 MHz, Chloroform-*d*) *δ* 8.8 (d, *J* = 7.6 Hz, 1H), 8.1 (dd, *J* = 8.8, 7.1 Hz, 1H), 7.2 (dd, *J* = 8.6, 2.0 Hz, 4H), 7.0 (tt, *J* = 7.2, 2.0 Hz, 4H), 6.8–6.6 (m, 2H), 5.5 (dt, *J* = 8.5, 4.3 Hz, 1H), 4.7 (hept, *J* = 6.1 Hz, 1H), 4.4 (d, *J* = 4.4 Hz, 1H), 3.3 (s, 3H), 1.4 (dd, *J* = 10.3, 6.1 Hz, 6H). ^13^C NMR (101 MHz, cdcl_3_) *δ* 165.0, 164.5, 157.6, 136.2, 134.8, 134.7, 133.9, 129.1, 128.7, 128.6, 108.4, 108.2, 101.4, 101.1, 72.5, 59.3, 58.4, 22.1, 22.0.

#### 3.1.11. General CDI Coupling Procedure

A previously dried 100 mL round bottom flask under Ar atmosphere was filled with a solution of amino amide **17a–b** (0.25 mmol) in freshly distilled DCM (20 mL) and (0.3 mmol, 1.2 equivalents) CDI were added. After complete conversion to imidazoyl intermediate, which was monitored by mass spectrometry (MS ESI), piperazine or piperazine–DCA **9** (0.25 mmol, 1 equivalent) was added and the reaction was stirred overnight at room temperature. The reaction was quenched with water and transferred into a separatory funnel in order to wash the mixture three times with water and brine. The organic phase was separated, dried over Na_2_SO_4_ and the crude material was purified on silica gel chromatography with gradient elution, to give compound **18a–c**.

#### 3.1.12. General DABAL-Me_3_ Coupling Procedure

In a 50 mL round-bottom flask, previously dried and equipped with a magnetic stirrer and reflux condenser, three vacuum-argon cycles were performed to create an inert atmosphere. Anhydrous piperazine (0.88 mmol, 1.2 equivalents) and 226 mg (0.88 mmol, 1.2 equivalents) of DABAL-Me_3_ were dissolved in 10 mL of anhydrous toluene. The mixture was stirred for 1 h at 40 °C; then, desymmetrised amido carbamate **16a–c** (0.74 mmol) was added. The temperature was increased to 90 °C and the mixture was stirred for additional 2 h. After this time, the reaction was stopped and the solvent was removed to dry using a rotary evaporator. The residue was dissolved in DCM and washed with water three times. The organic phase was dried over Na_2_SO_4_, filtered through cotton and concentrated using a rotary evaporator. The crude product was purified by flash chromatography on silica gel, affording pure enantioenriched products **18a–b**.

##### *N*-(1,2-Bis(4-chlorophenyl)-2-(2-isopropoxy-4-methoxybenzamido)ethyl)piperazine-1-carboxamide (**18a**)

- CDI coupling method: 96% yield (MS ESI Imidazoyl intermediate [M + H]^+^_found_ = 567.71).
- DABAL-Me_3_ coupling method: 58% yield.

Product ESI [M + H]^+^_calc_ = 585.20, ESI [M + H]^+^_found_ = 585.9. TLC: (CH_2_Cl_2_/MeOH 9.5:0.5) R_f_ = 0.45. ^1^H NMR (400 MHz, Chloroform-*d*) *δ* 8.4 (d, *J* = 8.2 Hz, 1H), 8.3 (dd, *J* = 8.8, 2.0 Hz, 1H), 7.3 (dd, *J* = 8.3, 1.4 Hz, 2H), 7.2–7.1 (m, 2H), 7.0–6.9 (m, 3H), 6.9–6.8 (m, 2H), 6.6 (dd, *J* = 8.9, 2.3 Hz, 1H), 6.5–6.4 (m, 1H), 5.8 (dd, *J* = 8.2, 2.6 Hz, 1H), 5.1 (dd, *J* = 5.2, 2.4 Hz, 1H), 4.7 (*p*, *J* = 6.0 Hz, 1H), 3.4 (tdt, *J* = 18.2, 12.9, 5.8 Hz, 3H), 2.9 (t, *J* = 5.2 Hz, 3H), 1.2 (d, *J* = 6.0 Hz, 4H), 1.2 (dd, *J* = 5.9, 2.7 Hz, 3H). ^13^C NMR (101 MHz, cdcl_3_) *δ* 167.1, 164.1, 157.5, 157.2, 134.5, 133.3, 129.5, 128.8, 128.6, 128.2, 113.9, 105.5, 100.5, 71.6, 61.9, 57.7, 55.8, 46.1, 44.9, 22.1, 21.7.

##### *N*-(1,2-Bis(4-chlorophenyl)-2-(4-fluoro-2-isopropoxybenzamido)ethyl)piperazine-1-carboxamide (**18b**)

- CDI coupling method: 70% yield. (MS ESI Imidazoyl intermediate [M + H]^+^_found_ = 555.1).
- DABAL-Me_3_ coupling method: 40% yield.

Product ESI [M + H]^+^_calc_ = 573.18, ESI [M + H]^+^_found_ = 573.44. TLC: (CH_2_Cl_2_/MeOH 9:1) R_f_ = 0.17., ^1^H NMR (400 MHz, Methanol-*d*_4_) *δ* 7.5 (ddt, *J* = 8.6, 6.9, 1.2 Hz, 1H), 7.3–7.1 (m, 3H), 6.7 (dd, *J* = 11.1, 2.3 Hz, 1H), 6.6–6.5 (m, 1H), 5.6 (dd, *J* = 9.1, 1.9 Hz, 1H), 5.1 (d, *J* = 9.1 Hz, 1H), 4.6 (dt, *J* = 12.2, 6.3 Hz, 1H), 3.5–3.3 (m, 5H), 3.0–2.9 (m, 4H), 1.1–1.0 (m, 6H). ^13^C NMR (101 MHz, cd_3_od) *δ* 139.9, 139.5, 134.7, 130.7, 130.4, 129.7, 129.5, 73.7, 59.7, 57.4, 44.3, 42.2, 22.1, 21.9.

##### *N*-(1,2-Bis(4-chlorophenyl)-2-(2-isopropoxy-4-methoxybenzamido)ethyl)-4-(3-(2,2-dichloroacetamido)propyl)piperazine-1-carboxamide (**18c**)

CDI coupling method: 40% yield. Imidazoyl intermediate: MS ESI [M + H]^+^ = 567.71. TLC (CH_2_Cl_2_/MeOH 9.5:0.5): R_f_ = 0.43. Product: ESI [M + H]^+^_calc_ = 752.19, ESI [M + H]^+^_found_ = 752.52. ^1^H NMR (400 MHz, Chloroform-*d*) *δ* 8.5 (s, 1H), 8.4 (d, *J* = 8.0 Hz, 1H), 8.2 (dd, *J* = 8.8, 1.3 Hz, 1H), 7.5 (d, *J* = 4.9 Hz, 1H), 7.3–7.2 (m, 3H), 7.2–7.1 (m, 2H), 7.0 (dd, *J* = 8.5, 6.9 Hz, 2H), 6.9–6.8 (m, 2H), 6.6 (dd, *J* = 8.9, 2.2 Hz, 1H), 6.5 (d, *J* = 2.2 Hz, 1H), 5.9 (d, *J* = 1.6 Hz, 1H), 5.8 (dd, *J* = 8.0, 2.4 Hz, 1H), 5.1 (dt, *J* = 7.5, 3.7 Hz, 1H), 4.7 (h, *J* = 6.1 Hz, 1H), 3.8 (d, *J* = 1.3 Hz, 3H), 3.5 (q, *J* = 4.7 Hz, 4H), 3.4 (tt, *J* = 6.2, 2.8 Hz, 3H), 2.5 (dt, *J* = 17.1, 5.4 Hz, 7H), 2.4 (d, *J* = 5.1 Hz, 2H), 1.8–1.7 (m, 4H), 1.2 (dd, *J* = 19.8, 6.1 Hz, 6H). ^13^C NMR (101 MHz, cdcl_3_) *δ* 200.8, 200.2, 167.3, 164.5, 164.1, 157.5, 156.9, 136.9, 136.7, 134.4, 133.3, 129.5, 128.8, 128.6, 128.2, 113.7, 105.5, 100.4, 77.5, 71.6, 66.9, 62.1, 58.5, 57.7, 55.8, 53.4, 43.5, 41.4, 29.8, 28.5, 23.8, 22.1, 21.6.

#### 3.1.13. General Ring Closure Catalysed by Hendrickson’s Reagent

In a previously dried 100 mL round-bottom flask, equipped with a magnetic stirrer under inert atmosphere, Ph_3_PO (0.7 mmol, 4 equivalents) was dissolved in 10 mL of distilled DCM and, Tf_2_O (0.35 mmol, 2 equivalents) were added with a syringe. The mixture was allowed to react for 2 h. Next, amido urea **18a–b** (0.2 mmol, 1 equivalent) was added and the reaction was stirred for an additional 2 h. Then the solvent was removed to dry using a rotary evaporator. The resulting residue was purified by flash chromatography on silica gel.

##### (4,5-Bis(4-chlorophenyl)-2-(2-isopropoxy-4-methoxyphenyl)-4,5-dihydro-1H-imidazol-1-yl)(piperazin-1-yl)methanone (**19a**)

A 43% yield; TLC (DMC/MeOH 9:1), ESI [M + H]^+^_calc_ = 567.19, ESI [M + H]^+^_found_ = 567.02. Preparative RP-HPLC: (solvent A = H_2_O + 0.1% TFA, solvent B = H_2_O/ACN (40:60) + 0.1% TFA, gradient from 35% of B to 95% B in 20 min; flow 20 mL/min; t_R_: 21.3 min). Chiral HPLC (Whelk01 150 mm × 4.6 mm, 5 µm. % MP solvent system A = H_2_O, B = ACN: from 20% to 40% of B in 40 min. Flow = 1 mL/min. UV: 300 nm): t_R_[(1S,2R)-**19a**] = 28.2 min (minor ent), t_R_[(1R,2S)-**19a**] = 29.8 min (major ent) (ee_%_ = 89%). ^1^H NMR (400 MHz, DMSO-*d*_6_) *δ* 7.4 (d, *J* = 8.3 Hz, 1H), 7.2–7.1 (m, 4H), 7.1–7.0 (m, 2H), 7.0–6.9 (m, 2H), 6.7–6.6 (m, 2H), 5.6 (d, *J* = 10.0 Hz, 1H), 5.5 (d, *J* = 10.0 Hz, 1H), 4.7 (*p*, *J* = 6.0 Hz, 1H), 3.8 (s, 3H), 3.0 (d, *J* = 5.2 Hz, 4H), 2.2 (s, 4H), 1.8 (d, *J* = 12.3 Hz, 2H), 1.3 (dd, *J* = 14.6, 6.0 Hz, 7H). ^13^C NMR (101 MHz, dmso) *δ* 162.2, 160.2, 156.8, 154.8, 137.6, 136.7, 131.7, 131.1, 129.8, 128.8, 127.5, 113.7, 104.7, 99.4, 70.7, 69.9, 67.9, 55.5, 46.6, 44.9, 21.8, 21.8.

^1^H NMR (400 MHz, Methanol-*d*_4_) *δ* 7.8–7.8 (m, 1H), 7.3–7.2 (m, 4H), 7.2–7.0 (m, 4H), 6.9–6.8 (m, 2H), 6.2 (d, *J* = 10.8 Hz, 1H), 6.1 (d, *J* = 10.8 Hz, 1H), 4.9 (dt, *J* = 12.4, 6.2 Hz, 1H), 3.5 (t, *J* = 5.3 Hz, 4H), 2.9 (tq, *J* = 13.0, 6.6, 5.3 Hz, 4H), 1.5 (d, *J* = 6.0 Hz, 3H), 1.4 (d, *J* = 6.0 Hz, 3H). ^13^C NMR (101 MHz, cd_3_od) *δ* 168.7, 160.0, 152.2, 136.0, 134.4, 133.8, 133.1, 132.7, 132.5, 130.6, 130.4, 129.9, 129.8, 129.6, 108.2, 105.1, 101.6, 74.1, 70.8, 65.1, 56.8, 44.0, 22.1, 22.2.

##### (4,5-Bis(4-chlorophenyl)-2-(4-fluoro-2-isopropoxyphenyl)-4,5-dihydro-1H-imidazol-1-yl)(piperazin-1-yl)methanone (**19b**)

A 41% yield; TLC: (CH_2_Cl_2_/MeOH 9:1) R_f_ = 0.37. ESI [M + H]^+^_calc_ = 555.2, ESI [M + H]^+^_found_ = 555.46. ^1^H NMR (400 MHz, Methanol-*d*_4_) *δ* 8.0 (s, 1H), 7.9 (dd, *J* = 8.7, 6.2 Hz, 1H), 7.3–7.1 (m, 9H), 7.0 (ddd, *J* = 8.6, 7.8, 2.2 Hz, 1H), 6.3 (d, *J* = 11.3 Hz, 1H), 6.2 (d, *J* = 11.3 Hz, 1H), 5.0 (h, *J* = 6.0 Hz, 1H), 3.6 (ddd, *J* = 6.5, 4.3, 2.2 Hz, 4H), 3.0–2.9 (m, 7H), 2.9 (s, 2H), 1.5 (d, *J* = 6.0 Hz, 3H), 1.4 (d, *J* = 6.0 Hz, 3H). ^13^C NMR (101 MHz, cd_3_od) *δ* 170.6, 168.1, 167.2, 160.0, 159.9, 151.7, 136.2, 136.1, 134.4, 134.3, 132.4, 132.4, 130.7, 130.6, 130.4, 129.8, 129.7, 109.8, 109.6, 103.6, 103.4, 74.8, 70.9, 65.5, 44.1, 43.8, 37.1, 31.8, 30.7, 22.2, 22.0. ^19^F NMR (376 MHz, cd_3_od) *δ* -80.0. ^1^H NMR (400 MHz, Chloroform-*d*) *δ* 7.5 (dd, *J* = 8.4, 6.6 Hz, 1H), 7.1–7.0 (m, 2H), 7.1–7.0 (m, 2H), 7.0–6.9 (m, 2H), 6.9–6.8 (m, 2H), 6.8–6.7 (m, 2H), 5.6 (d, *J* = 10.0 Hz, 1H), 5.5 (d, *J* = 10.0 Hz, 1H), 4.6 (*p*, *J* = 6.1 Hz, 1H), 3.1 (t, *J* = 4.9 Hz, 4H), 2.4 (s, 4H), 1.4 (dd, *J* = 18.0, 6.1 Hz, 6H). ^13^C NMR (101 MHz, cdcl_3_) *δ* 166.4, 163.9, 160.3, 157.5, 155.3, 136.4, 135.3, 133.3, 133.0, 132.2, 132.1, 129.4, 128.6, 128.3, 128.1, 107.4, 107.1, 101.1, 100.8, 71.9, 71.6, 69.2, 46.8, 45.5, 29.8, 22.2.^19^F NMR (376 MHz, cdcl_3_) *δ* −75.9.

#### 3.1.14. N-(3-(4-(4,5-Bis(4-chlorophenyl)-2-(2-isopropoxy-4-methoxyphenyl)-4,5-dihydro-1H-imidazole-1-carbonyl)piperazin-1-Yl)propyl)-2,2-bichloroacetamide (**20a**)

A previously dried 100 mL round bottom flask was filled with 146 mg (0.26 mmol) of compound **19a**, 105 mg (0.78 mmol, 3 equivalents) of K_2_CO_3_ and 64 mg (0.26 mmol, 1 equivalent) of bromide **9** in 20 mL of ACN. The reaction was heated until reflux and stirred overnight. The solvent was removed to dry and the residue mixture was purified by preparative RP-HPLC: (solvent A = H_2_O + 0.1% TFA, solvent B = H_2_O/ACN (40:60) + 0.1% TFA, gradient from 30% B to 90% B in 25 min; flow 20 mL/min; R_t_: 28.5 min) to give 124 mg of **20a** (65% yield). MS ESI: [M + H]^+^_calc_ = 734.2, [M + H]^+^_found_ = 734.38; 736.38. Chiral HPLC (Whelk01 150 mm × 4.6 mm, 5 µm. % MP solvent system A = H_2_O, B = ACN: from 30% to 50% of B in 40 min. Flow = 1 mL/min. UV: 300 nm): t_R_[(4S,5R)-**20a**] = 19.1 min (minor ent), t_R_[(4R,5S)-**20a**] = 20.6 min (major ent)(ee_%_ = 88.2%). ^1^H NMR (400 MHz, DMSO-d6) *δ* 11.0 (t, *J* = 5.7 Hz, 1H), 10.6 (s, 1H), 10.0–9.8 (m, 5H), 9.6–9.4 (m, 4H), 9.4–9.2 (m, 4H), 9.1–9.0 (m, 2H), 8.8 (s, 1H), 8.2 (d, *J* = 33.6 Hz, 2H), 7.2–7.1 (m, 1H), 6.2 (s, 3H), 5.4 (q, *J* = 6.6 Hz, 2H), 5.3–5.1 (m, 3H), 4.0 (d, *J* = 8.0 Hz, 3H), 3.6 (d, *J* = 6.0 Hz, 3H), 3.5 (d, *J* = 6.0 Hz, 3H). ^13^C NMR (101 MHz, dmso) *δ* 163.9, 157.3, 133.2, 132.7, 132.0, 131.5, 131.4, 129.6, 129.0, 128.8, 128.7, 127.8, 105.8, 99.7, 79.2, 70.9, 68.1, 66.8, 55.9, 53.5, 50.5, 42.5, 36.7, 23.2, 21.7, 21.6.

#### 3.1.15. 4-Fluoro-2-isopropoxybenzoic Anhydride (**23b**)

In a mortar, 581 mg (2.9 mmol) of acid **3b** and 1.7 g (12,7 mmol, 4.3 equivalents) of K_2_CO_3_ were mixed with a pestle. A solution of 346 mg (1.8 mmol) of tosyl chloride (TsCl) in 4 mL of AcOEt was added dropwise and mixed for 20 min. A few drops of AcOEt were added to obtain a uniform mixture. The resulting mixture were diluted with 20 mL of DCM and filtered through a Gooch crucible. The filtrate was dried using a rotary evaporator. Then, 332 mg (0.9 mmol, 60% yield) of anhydride **23b**, an amorphous compound, was obtained. R_f_ (solvent system: AcOEt/Petroleum ether 1:9) = 0.45.

#### 3.1.16. General Procedure for Racemic Monoamide (**17**)

In a two-neck 250 mL round-bottom flask, equipped with a magnetic stirrer and previously dried, under inert atmosphere, *meso*-diamine **5** (1 mmol) was dissolved in 40 mL of toluene. Then, a solution of DMAP (1.5 mmol, 1.5 eq) in 20 mL of toluene was added. After 10 min, a solution of anhydride **23a–b** (0.8 mmol, 0.8 equivalents) in 30 mL of toluene was slowly added via dropping funnel. The mixture was stirred for three hours then the solvent was removed to dry with rotary evaporator. The residue was dissolved in AcOEt and transferred in a separatory funnel, washed three times with water and then three times with brine. The organic phase was dried over anhydrous Na_2_SO_4_, filtered through a cotton plug and solvent was evaporated to dry with a rotary evaporator. The crude solid was purified by gradient column chromatography on silica gel, affording monoamide products as a racemic mixture. *rac*-**17a** (53% yield), *rac*-**17b** (50% yield).

#### 3.1.17. Methyl 4-Fluoro-2-isopropoxybenzoate (**24**)

In a 100 mL two-neck flask equipped with a mechanical stirrer and a reflux condenser, 500 mg (2.5 mmol) of compound **3b** were dissolved in MeOH and 202 µL (2.8 mmol, 1.1 equivalents) of thionyl chloride (SOCl_2_) were slowly added with a syringe. The flask was immersed in a silicone oil bath on a heating plate until reflux. The mixture was left to react overnight at reflux temperature. The flask was then allowed to cool to room temperature and the solvent was removed to dry using under reduced pressure. The residue was dissolved in DCM and washed with H_2_O three times. The organic phase was dried, filtered through a cotton plug and concentrated to dry using a rotary evaporator. A total of 525 mg (2.5 mmol, 98%) of methyl ester **24** were obtained as a brownish oil. R_f_ (solvent system: AcOEt/Petroleum ether 1:1) = 0.91. ESI [M + H]^+^_calc_ = 213,09, ESI [M + H]^+^_found_ = 213.13. ^1^H NMR (400 MHz, Chloroform-*d*) *δ* 7.8 (ddd, *J* = 8.3, 7.0, 0.6 Hz, 1H), 6.7–6.6 (m, 2H), 4.5 (hept, *J* = 6.1, 0.6 Hz, 1H), 3.9 (s, 3H), 1.4 (d, *J* = 6.1 Hz, 6H).

#### 3.1.18. 4,5-Bis(4-chlorophenyl)-2-(4-fluoro-2-isopropoxyphenyl)-4,5-dihydro-1H-imidazole (**25**)

In a microwave vial equipped with a magnetic stirrer, 500 mg (1.79 mmol) of *meso*-diammine **5**, 406 mg (2.13 mmol, 1.1 equivalents) of methyl ester **24** and 456 mg (1.79 mmol, 1 equivalent) of DABAL-Me_3_ were dissolved in 20 mL of toluene. The mixture was reacted in a microwave for 90 min at 130 °C. Subsequently, the toluene was removed to dry using a rotary evaporator and the crude product was purified by flash chromatography (10–100% DCM/MeOH) on silica gel, yielding 400 mg of product **25** (yield 50%), a yellow amorphous solid. TLC: (DCM/MeOH 9.5:0.5): R_f_ = 0.30. MS ESI: [M + H]^+^_calc_ = 443.11, [M + H]^+^_found_ = 443.32. ^1^H NMR (400 MHz, Chloroform-*d*) *δ* 8.2 (dd, *J* = 8.7, 7.0 Hz, 1H), 7.1–7.0 (m, 4H), 6.9–6.8 (m, 4H), 6.8–6.6 (m, 2H), 5.4 (s, 2H), 4.7 (hept, *J* = 6.1 Hz, 1H), 1.4 (d, *J* = 6.1 Hz, 6H). ^13^C NMR (101 MHz, cdcl_3_) *δ* 166.6, 164.1, 163.6, 157.6, 157.5, 137.7, 133.6, 133.5, 132.7, 128.9, 128.6, 128.0, 115.2, 108.4, 108.2, 101.5, 101.3, 72.0, 69.6, 22.1. ^19^F NMR (376 MHz, cdcl_3_) *δ* −106.2.

#### 3.1.19. *tert*-Butyl 4-(4,5-Bis(4-chlorophenyl)-2-(4-fluoro-2-isopropoxyphenyl)-4,5-dihydro-1H-imidazole-1-carbonyl)piperazine-1-carboxylate (**26**)

In a 100 mL round-bottom flask equipped with a magnetic stirrer, an inert atmosphere was created by performing three vacuum-argon cycles. Then, 80 mg (0.180 mmol) of imidazoline **25** was dissolved in 1.6 mL of freshly distilled DCM and 283 µL (1.62 mmol, 9 equivalents) of DIPEA were added at 0 °C. To the clear solution, a solution of triphosgene (40 mg, 0.135 mmol, 0.75 equivalents) in 1.6 mL of anhydrous DCM was added at 0 °C. The mixture was stirred for 3 h at room temperature. Next, 54 mg of *N*-Boc-Piperazine dissolved in 0.8 mL of anhydrous DCM (0.288 mmol, 1.6 equivalents) were added. The reaction was stirred for an additional 3 h at room temperature. After quenching the reaction with water, the heterogeneous mixture was transferred into a separatory funnel and washed twice with water. The organic phase was dried over Na_2_SO_4_, filtered through a cotton plug and the solvent was removed to dry under reduced pressure. The crude product was purified by flash chromatography (5–15% MeOH/DCM) on silica gel, yielding 80 mg of pure compound **26**, a light-yellow solid (71% yield). TLC: (CH_2_Cl_2_/MeOH 9.5:0.5) R_f_ = 0.70. ESI [M + H]^+^_calc_ = 655.22, ESI [M + H]^+^_found_ = 655.63.

#### 3.1.20. (4,5-Bis(4-chlorophenyl)-2-(4-fluoro-2-isopropoxyphenyl)-4,5-dihydro-1H-imidazol-1-Yl)(piperazin-1-Yl)methanone (*rac*-**19b**)

In a round-bottom 100 mL flask equipped with a magnetic stirrer, 80 mg (0.122 mmol) of compound **26** were dissolved in 4 mL of DCM. After adding 280 µL (3.66 mmol, 30 equivalents) of trifluoracetic acid (TFA), the solution was allowed to react overnight at room temperature. The following day, complete consumption of starting material was detected and the reaction was quenched by adding a saturated solution of NaHCO_3_ to reach basic pH (9). The mixture was stirred for 30 min and transferred in a separatory funnel. The two layers were separated and organic phase was washed three times with water then dried over Na_2_SO_4_, filtered through a funnel with cotton. The solvent was removed to dry using a rotary evaporator. Finally, 80 mg of deprotected racemic product *rac*-**19b**, was obtained as a light orange solid (90% yields).

#### 3.1.21. N-(3-(4-(4,5-Bis(4-chlorophenyl)-2-(4-fluoro-2-isopropoxyphenyl)-4,5-dihydro-1H-imidazole-1-carbonyl)piperazin-1-Yl)propyl)-2,2-dichloroacetamide (*rac*-**20b**)

In a previously dried 100 mL round-bottom flask, equipped with a magnetic stirrer, an inert atmosphere was created by performing three vacuum-argon cycles. A solution of 100 mg (0.226 mmol) of imidazoline **25** in 2 mL of freshly distilled DCM was mixed with 354 µL (2.034 mmol, 9 equivalents) of DIPEA at 0 °C. To the clear solution, a solution of triphosgene (50.45 mg, 0.170 mmol, 0.75 equivalents) in 2 mL of anhydrous DCM was added at 0 °C. The mixture was stirred for 3 h at room temperature. Next, 92 mg of compound 2,2-dichloro-*N*-(3-(piperazin-1-yl)propyl)acetamide **9** (0.362 mmol, 1.6 equivalents) were added and the reaction was stirred for an additional 2.5 h at room temperature. After quenching with water, the mixture was transferred in a separatory funnel and washed with distilled water twice. The organic phase was dried over Na_2_SO_4_, filtered through a cotton plug and the solvent was removed to dry using a rotary evaporator. The resulting residue was purified by flash chromatography (10–20% DCM/MeOH) on silica gel, yielding 60 mg of racemic product *rac*-**20b**, a pale orange solid (37% yield). TLC: (DCM/MeOH 9.5:0.5): R_f_ = 0,74. MS ESI: [M + H]^+^_calc_ = 722.16, [M + H]^+^_found_ = 724.68. ^1^H NMR (400 MHz, Chloroform-*d*) *δ* 8.2 (s, 1H), 7.6–7.5 (m, 1H), 7.1–7.0 (m, 4H), 7.0–6.9 (m, 2H), 6.8 (d, *J* = 7.6 Hz, 2H), 6.8–6.7 (m, 2H), 5.9 (s, 1H), 5.6 (d, *J* = 9.9 Hz, 1H), 5.5 (d, *J* = 10.0 Hz, 1H), 4.6 (h, *J* = 6.0 Hz, 1H), 3.4 (q, *J* = 5.5 Hz, 2H), 3.2–3.1 (m, 3H), 2.4 (t, *J* = 5.7 Hz, 2H), 2.1 (d, *J* = 9.7 Hz, 4H), 1.7 (*p*, *J* = 5.9 Hz, 2H), 1.4 (d, *J* = 6.1 Hz, 3H), 1.4 (d, *J* = 6.1 Hz, 3H). ^13^C NMR (101 MHz, cdcl_3_) *δ* 166.4, 164.4, 163.9, 136.3, 135.2, 133.3, 133.0, 132.2, 129.3, 128.6, 128.3, 128.1, 107.5, 107.2, 101.1, 100.9, 71.9, 71.6, 69.2, 66.8, 58.1, 52.9, 45.4, 41.1, 29.8, 23.8, 22.2, 22.1.

#### 3.1.22. 6-(4-(4,5-Bis(4-chlorophenyl)-2-(2-isopropoxy-4-methoxyphenyl)-4,5-dihydro-1H-imidazole-1-carbonyl)piperazin-1-yl)-N-(1,3-dihydroxy-2-(hydroxymethyl)propan-2-Yl)hexanamide (**21**)

A previously dried 100 mL round bottom flask was filled with 100 mg (0.17 mmol) of compound **19a**, 83 mg 0.6 mmol, 3 equivalents) of K_2_CO_3_ and 60 mg (0.20 mmol, 1.2 equivalents) of bromide **12** in 10 mL of ACN. The temperature of the flask was gradually increased until reflux and the reaction was stirred overnight. The solvent was removed to dry and the residue mixture was purified by isocratic chromatography (Solvent system DCM/MeOH/Toluene 17:1:2) on silica gel, to obtain 30 mg, 0.04 mmol, 23% yield) of product **21**. ESI [M + H]^+^_calc_ = 784.32, ESI [M + H]^+^_found_ = 784.89. ^1^H NMR (400 MHz, Methanol-*d*_4_) *δ* 7.6–7.5 (m, 1H), 7.2–7.0 (m, 8H), 6.9 (d, *J* = 8.1 Hz, 2H), 6.7–6.7 (m, 2H), 5.8 (d, *J* = 10.2 Hz, 1H), 5.5 (d, *J* = 10.3 Hz, 1H), 4.7 (*p*, *J* = 6.0 Hz, 1H), 3.9 (s, 3H), 3.7 (s, 7H), 3.3–3.1 (m, 4H), 2.2 (dt, *J* = 10.3, 7.1 Hz, 4H), 2.2–1.9 (m, 4H), 1.6 (*p*, *J* = 7.5 Hz, 2H), 1.4 (d, *J* = 6.0 Hz, 3H), 1.4 (d, *J* = 6.0 Hz, 3H), 1.3 (d, *J* = 5.3 Hz, 6H), 1.0–0.8 (m, 1H). ^13^C NMR (101 MHz, cd_3_od) *δ* 177.2, 165.0, 158.8, 156.4, 137.8, 136.6, 134.2, 132.9, 130.7, 130.0, 129.2, 129.0, 114.2, 106.4, 101.4, 72.4, 71.7, 70.0, 63.6, 62.7, 59.2, 56.2, 53.2, 49.9, 49.6, 49.4, 49.2, 49.0, 48.8, 48.6, 48.4, 46.6, 37.3, 27.9, 26.7, 22.5, 22.4. Dialkylated compound **27** was also isolated from crude mixture by chromatography to obtain (MS ESI [M + H]^+^_calc_^=^ 1001.46, ESI [M + H]^+^_found_ = 1002.4).

#### 3.1.23. 2-(6-(4-(4,5-Bis(4-chlorophenyl)-2-(2-isopropoxy-4-methoxyphenyl)-4,5-dihydro-1H-imidazole-1-carbonyl)piperazin-1-Yl)hexanamido)-2-((2,2-dichloroacetoxy)methyl)propane-1,3-diyl Bis(2,2-dichloroacetate) (**22**)

A previously dried 50 mL round-bottom flask was filled with 65 mg (0.08 mmol, 1 equivalent) of compound **21** and 5 mL of freshly distilled DCM. Then 24 µL (0.25 mmol, 3 equivalents) of dichloroacetic chloride **7** was added to the solution. The reaction was stirred at r.t. overnight then the solvent was evaporated under reduced pressure. The product was purified from the crude by gradient concentration chromatography (0–15% DCM/MeOH) on silica gel. Finally, the fractions were collected to afford 8 mg (9% yield) of finale product **22**. TLC: (DCM/MeOH 9.5:0.5) R_f_ = 0.3. ESI [M + H]^+^_calc_ = 1114.12, ESI [M + H]^+^_found_ = 1118.42, 1006.42 [M–DCA], 896.55 [M–2DCA]. ^1^H NMR (500 MHz, Chloroform-d) *δ* 9.84 (s, 1H), 7.53 (dd, *J* = 8.6, 1.9 Hz, 1H), 7.44 (dd, *J* = 7.3, 1.7 Hz, 1H), 7.42–7.35 (m, 1H), 7.12–7.05 (m, 2H), 7.07–7.00 (m, 2H), 6.96–6.90 (m, 2H), 6.87–6.82 (m, 2H), 6.70 (s, 1H), 6.57 (dd, *J* = 8.5, 2.3 Hz, 1H), 5.59 (d, *J* = 9.8 Hz, 1H), 5.45 (d, *J* = 9.8 Hz, 1H), 4.61 (h, *J* = 6.0 Hz, 1H), 3.95 (s, 1H), 3.86 (s, 2H), 3.64 (s, 4H), 3.25 (s, 4H), 2.38 (s, 2H), 2.23 (dt, *J* = 13.7, 7.0 Hz, 2H), 1.66 (*p*, *J* = 7.0 Hz, 2H), 1.51 (s, 2H), 1.40 (d, *J* = 6.0 Hz, 3H), 1.34 (d, *J* = 6.0 Hz, 3H). ^13^C NMR (101 MHz, cdcl_3_) *δ* 191.1, 175.1, 163.2, 157.5, 155.0, 136.2, 135.2, 133.3, 133.1, 132.2, 130.4, 129.4, 128.9, 128.6, 128.4, 128.3, 128.1, 127.3, 126.7, 113.7, 112.5, 109.5, 104.7, 100.5, 71.6, 71.3, 71.0, 69.3, 64.3, 61.5, 57.8, 56.2, 55.8, 44.4, 36.5, 29.8, 26.2, 24.8, 22.3.

#### 3.1.24. 1,1-Bis(6-((1,3-bis(2,2-dichloroacetoxy)-2-((2,2-dichloroacetoxy)methyl)propan-2-Yl)amino)-6-oxohexyl)-4-(4,5-bis(4-chlorophenyl)-2-(2-isopropoxy-4-methoxyphenyl)-4,5-dihydro-1H-imidazole-1-carbonyl)piperazin-1-ium (**28**)

A previously dried 50 mL round-bottom flask was filled with 65 mg (0.08 mmol, 1 equivalent) of compound **27** and 5 mL of freshly distilled DCM. Then 24 µL (0.25 mmol, 3 equivalents) of dichloroacetic chloride **7** was added to the solution. The reaction was stirred at r.t. overnight then the solvent was evaporated under reduced pressure. The product **28** was purified from the crude through Preparative RP-HPLC: (solvent A = H_2_O + 0.1% TFA, solvent B = H_2_O/ACN (40:60) + 0.1% TFA, gradient from 10% B to 80% of B in 20 min then isocratic 100% of B for 15 min; flow 20 mL/min; t_R_: 33 min). ESI [M]^+^_calc_ = 1661.05, ESI [M]^+^_found_ = 1667.1, 1556.2 [M–DCA].

### 3.2. Biological Evaluation

#### 3.2.1. Cell Lines

The biological effect of the different chimeric Nutlin-DCA and commercial Nutlin (#10004372 Cayman, Ann Arbor, Michigan, USA) compounds, resuspended in DMSO and stored at -20 °C in single-use aliquots, was evaluated using three cancer cell lines. MCF7 breast cancer cell line, expressing p53 wild-type, was purchased from Lonza (Basel, Switzerland, CH) and the two isogenic colon cancer cell lines, HCT-116 p53 wild-type (WT) and HCT-116 p53 knockout (KO), were obtained from the American Type Culture Collection (ATCC, Manassas, VA, USA). Cells were maintained in Dulbecco’s Modified Eagle Medium (DMEM; Corning, Glendale, AZ, USA), supplemented with 10% fetal bovine serum (FBS; Gibco, Grand Island, NY, USA), 2 mM L-glutamine, 100 U/mL penicillin and 100 µg/mL streptomycin (L-glutamine–penicillin–streptomycin solution; Sigma-Aldrich, St. Louis, MO, USA). All cell lines were cultured at 37 °C in a humidified atmosphere with 5% CO_2_, detached with trypsin (Sigma-Aldrich, St. Louis, MO, USA) for routine passage three times a week and used for the experiments at passage in the range of 7–20. All cell lines were checked for mycoplasma contamination (Invivogen, San Diego, CA, USA).

#### 3.2.2. Cell Viability Analysis by MTT Assay

The day before the experiments, MCF7, HCT-116^WT^ and HCT-116^KO^ cells were detached with trypsin, counted with trypan blue exclusion dye (Sigma-Aldrich, St. Louis, MO, USA), seeded at a density of 3.5 × 10^3^ cells/well in 96-well plates and cultured in complete medium for 24 h. On the day of the experiments, the cells were treated with chimeric Nutlin-DCA compounds and commercial Nutlin-3 at different predetermined concentrations: 0.1; 1; 5; and 10 μM for 24 h. For each experiment, untreated cultures were used as negative control and DMSO was used as control vehicle. Cell proliferation was assessed using the MTT colorimetric assay (Cell Proliferation Kit I, Roche Diagnostics Corporation, Indianapolis, IN, USA). This assay is based on the conversion of the yellow tetrazolium salt, 3-(4,5-dimethylthiazol-2-yl)-2,5-diphenyltetrazolium bromide (MTT), into a purple formazan product by mitochondrial dehydrogenases, in metabolically active cells. 24 h after treatments, 10 µL of MTT reagent was added to each well and the plates were incubated for 4 h at 37 °C. Subsequently, 100 µL of the solubilisation solution provided in the kit was added to each well and left overnight to dissolve the formazan crystals. Absorbances of every well was measured at 570 nm using the TECAN Infinite^®^ M Plex microplate reader (Tecan Trading AG, Männedorf, Switzerland, CH) to quantify cell viability.

#### 3.2.3. Statistical Analysis

For the evaluation of the biological effects, data results obtained from at least three independent experiments were analysed using the non-parametric Kruskal–Wallis test with GraphPad Prism software, version 8.0.1 (GraphPad Software, San Diego, CA, USA). Results were expressed as mean ± Standard Error of the Mean (SEM) of at least three independent experiments run in triplicate. Statistical significance was defined as *p* < 0.05.

## 4. Conclusions

DCA aims to revert the Warburg effect by inhibiting pyruvate dehydrogenase kinase (PDK), thereby reactivating oxidative phosphorylation. Simultaneously, Nutlin-3a restores the tumour suppressive function of p53 by blocking its antagonist MDM2. By linking these two pharmacophores, our chimeric molecules are designed to induce cell cycle arrest and apoptosis through a dual-action mechanism, offering a promising strategy for enhanced anticancer efficacy by simultaneously targeting two critical cancer hallmarks.

This study describes the rational design and multi-step synthesis of a novel class of chimeric Nutlin-DCA compounds, strategically engineered to simultaneously target distinct anticancer pathways. Our ambitious synthetic strategy incorporated a crucial chemoenzymatic step catalysed by an immobilised lipase, aiming for an efficient and stereoselective preparation of these complex molecules. The implementation of this asymmetric transformation, particularly given the inherent challenge lipase faces in desymmetrisation reactions, represents a synthetic achievement, highlighting the potential of biocatalysis to streamline the synthesis drug candidates. Additionally, this offers a greener and more precise avenue for accessing intricate molecular architectures. We refined key synthetic steps from earlier reports to achieve a more efficient overall synthesis of the Nutlin core.

Biological evaluation of the synthesised Nutlin–DCA chimeras in a panel of three cancer cell lines in vitro yielded promising results, with compounds *rac*-**19a**, *rac*-**19b**, *rac*-**20a**, *rac*-**20b** and enantioenriched **20a** leading to a statistically significant reduction in cell viability at micromolar concentrations. This finding underscores the therapeutic potential of our dual-targeting hypothesis to enhance efficacy compared to individual parent drugs.

Intriguingly, our biological assays revealed that the racemic mixtures of the Nutlin–DCA compounds exhibited greater antiproliferative activity than their enantiomerically enriched counterparts obtained through the enzymatic asymmetric procedure. While this finding might initially appear counterintuitive, it suggests a critical insight into the inherent complexities of enzyme–substrate interactions. It is plausible that the enzymatic reaction, while highly effective in catalysing the asymmetric transformation, undesirably favoured the formation of the less biologically potent enantiomer of the Nutlin-DCA precursor. This finding is consistent with what is known about structurally similar molecule Nutlin-3a, where the (4R, 5S) configuration is the less bioactive enantiomer. Therefore, we speculate that our enantioenriched compounds possess identical configuration. The unpredictability of enzymatic stereoselectivity in this context highlights a crucial aspect of biocatalysis: the specific enantiomer produced as the dominant product for a novel substrate cannot always be predicted a priori. This outcome provides invaluable lessons for future drug design and enzymatic catalysis. It underscores the trial-and-error nature inherent in exploring novel enzyme-substrate pairings and the nuanced influence of subtle variations in enzyme active site and substrate binding on the final stereochemical outcome.

Our work demonstrates a substantial synthetic effort culminating in successful implementation of a multi-step synthesis of dual-functional compounds. This research lays a solid foundation for further investigations, including thorough in vivo studies, to explore the full therapeutic potential of these chimeric compounds, reinforcing the need for continued exploration into synthetic methodologies and stereochemical outcomes in the pursuit of novel anticancer agents.

## Data Availability

The raw data supporting the conclusions of this article will be made available by the authors on request.

## Supplementary Materials

The following supporting information can be downloaded at: https://www.mdpi.com/article/10.3390/molecules30193908/s1, Figures: ^1^H NMR compound **2b**; ^13^C NMR compound **2b**^1^H NMR compound **3b**; ^13^C NMR compound **3b**; ^19^F NMR compound **3b**; ^1^H NMR compound ***N*-Boc-9**; ^13^C NMR compound ***N*-Boc-9**; ^1^H NMR compound **9**; ^13^C NMR compound **9**; ^1^H NMR compound **12**; ^13^C NMR compound **12**; ^1^H NMR compound **13**; ^13^C NMR compound **13**; ^1^H NMR compound **16a**; ^13^C NMR compound **16a**; ^1^H NMR compound **16b**; ^13^C NMR compound **16b**; ^1^H NMR compound **16c**; ^13^C NMR compound **16c**; ^19^F NMR compound **16c**; ^1^H NMR compound **16d**; ^13^C NMR compound **16d**; ^1^H NMR compound **17b**; ^13^C NMR compound **17b**; ^1^H NMR compound **18a**; ^13^C NMR compound **18a**; ^1^H NMR compound **18b**; ^13^C NMR compound **18b**; ^1^H NMR compound **18c**; ^13^C NMR compound **18c**; ^1^H NMR compound **18d**; ^13^C NMR compound **18d**; ^1^H NMR compound **19a**; ^13^C NMR compound **19a**; ^1^H NMR compound **19b**; ^13^C NMR compound **19b**; ^19^F NMR compound **19b**; ^1^H NMR compound **20a**; ^13^C NMR compound **20a**; ^1^H NMR compound **20b**; ^13^C NMR compound **20b**; ^1^H NMR compound **21**; ^13^C NMR compound **21**; ^1^H NMR compound **22**; ^13^C NMR compound **22**; ^1^H NMR compound **25**; ^13^C NMR compound **25**; 19F NMR compound 25; HPLC chromatogram compound **19a**; HPLC chromatogram compound *rac*-**19a**; HPLC chromatogram compound **19b**; HPLC chromatogram compound *rac*-**19b**; HPLC chromatogram compound *rac*-**20b**; Exact mass compound **18c**; Exact mass compound **19a**; Exact mass compound **19b**; Exact mass compound **20a**; Exact mass compound **20b**; Exact mass compound **22**; Exact mass compound **28**; Chiral HPLC Chromatogram compound **19a**; Chiral HPLC Chromatogram Compound **20a**; Biological results.

## Abbreviations

The following abbreviations are used in this manuscript:

DCA	dichloroacetate
PDK	pyruvate dehydrogenase kinase
PDH	pyruvate dehydrogenase
DABAL-Me_3_	Bis(trimethylaluminum)-1,4-diazabicyclo [2.2.2]octane adduct
EEDQ	2-Ethoxy-1-ethoxycarbonyl-1,2-dihydroquinoline
CAL-B	lipase from *Candida Antarctica* type B
MW	microwaves
WT	Wild type
KO	knockout
