# Supplementary material for: Multi-Step Synthesis of Chimeric Nutlin–DCA Compounds Targeting Dual Pathways for Treatment of Cancer
Source: Molecules. 2025 Sep 28;30(19):3908. doi: 10.3390/molecules30193908 (PMC12525975; doi:10.3390/molecules30193908)
Supplement: Supplementary file 1 [file molecules-30-03908-s001.zip › molecules-3821851-supplementary.pdf]

Supporting information

Multi-step synthesis of Chimeric Nutlin-DCA compounds Targeting Dual Pathways for treatment of cancer

Davide Illuminati[1], Rebecca Foschi[2], Paolo Marchetti[3], Vinicio Zanirato[3], Anna Fantinati[2], Claudio Trapella[3,4], Rebecca Voltan[2,4] and Virginia Cristofori [2,*]

[1] Department of Life Sciences, University of Modena and Reggio Emilia, via G. Campi 213/d, 41125 Modena, Italy; davide.illuminati@unife.it

[2] Department of Environmental and Prevention Sciences, University of Ferrara, via Luigi Borsari, 46, 44121 Ferrara, Italy; R.B. rebecca.foschi@unife.it, A.F. anna.fantinati@unife.it, R.V. rebecca.voltan@unife.it,

[3] Department of Chemical, Pharmaceutical and Agricultural Sciences, University of Ferrara, via Fossato di Mortara,17, 44121 Ferrara, Italy; P.M. paolo.marchetti@unife.it, V.Z. vinicio.zanirato@unife.it, C.T. claudio.trapella@unife.it, V.C. virginia.cristofori@unife.it

[4] Laboratorio per le Tecnologie delle Terapie Avanzate (LTTA), via Fossato di Mortara, 70, 44121 Ferrara, Italy

* Correspondence: virginia.cristofori@unife.it.

Table of contents

^{1}H NMR Compound 2b **S4**

^{13}C NMR Compound 2b **S5**

^{1}H NMR Compound 3b **S6**

^{13}C NMR Compound 3b **S7**

^{19}F NMR Compound 3b **S8**

^{1}H NMR Compound *N*-Boc-9 **S9**

^{13}C NMR Compound *N*-Boc-9 **S10**

^{1}H NMR Compound 9 **S11**

^{13}C NMR Compound 9 **S12**

^{1}H NMR Compound 12 **S13**

^{13}C NMR Compound 12 **S14**

^{1}H NMR Compound 13 **S15**

^{13}C NMR Compound 13 **S16**

^{1}H NMR Compound 16a **S17**

^{13}C NMR Compound 16a **S18**

^{1}H NMR Compound 16b **S19**

^{13}C NMR Compound 16b **S20**

^{1}H NMR Compound 16c **S21**

^{13}C NMR Compound 16c **S22**

^{1}H NMR Compound 16d **S23**

^{13}C NMR Compound 16d **S24**

^{19}F NMR Compound 16d **S25**

^{1}H NMR Compound 17b **S26**

^{13}C NMR Compound 17b **S27**

^{1}H NMR Compound 18a **S28**

^{13}C NMR Compound 18a **S29**

^{1}H NMR Compound 18b **S30**

^{13}C NMR Compound 18b **S31**

^{1}H NMR Compound 18c **S32**

^{13}C NMR Compound 18c **S33**

^{1}H NMR Compound 19a **S34**
- CD_3OD S34
- DMSO-d_6 S35

^{13}C NMR Compound 19a **S36**
- CD_3OD S36
- DMSO-d_6 S37

^{1}H NMR Compound 19b **S38**
- CD_3OD S38
- $CDCl_3$ S39

^{13}C NMR Compound 19b **S40**
- CD_3OD S40
- $CDCl_3$ S41

^{19}F NMR Compound 19b **S42**

^{1}H NMR Compound 20a **S43**

^{13}C NMR Compound 20a **S44**

^{1}H NMR Compound 20b **S45**

^{13}C NMR Compound 20b **S46**

^{1}H NMR Compound 21 **S47**

^{13}C NMR Compound 21 **S48**

^{1}H NMR Compound 22 **S49**

^{13}C NMR Compound 22 **S50**

^{1}H NMR Compound 25 **S51**

^{13}C NMR Compound 25 **S52**

^{19}F NMR Compound 25 **S53**

HPLC chromatogram compound 19a **S54**

HPLC chromatogram compound *rac*-19a **S56**

HPLC chromatogram compound 19b **S57**

HPLC chromatogram compound *rac*-19b **S58**

HPLC chromatogram compound *rac*-20b **S60**

Exact mass Compound 18c **S62**

Exact mass Compound 19a **S64**

Exact mass Compound 19b **S66**

Exact mass Compound 20a **S68**

Exact mass Compound 20b **S70**

Exact mass Compound 22 **S71**

Exact mass Compound 28 **S73**

Chiral HPLC Chromatogram Compound 19a **S75**

Chiral HPLC Chromatogram Compound 20a **S77**

Biological results **S79**

SS

^{1}H NMR Compound 2b

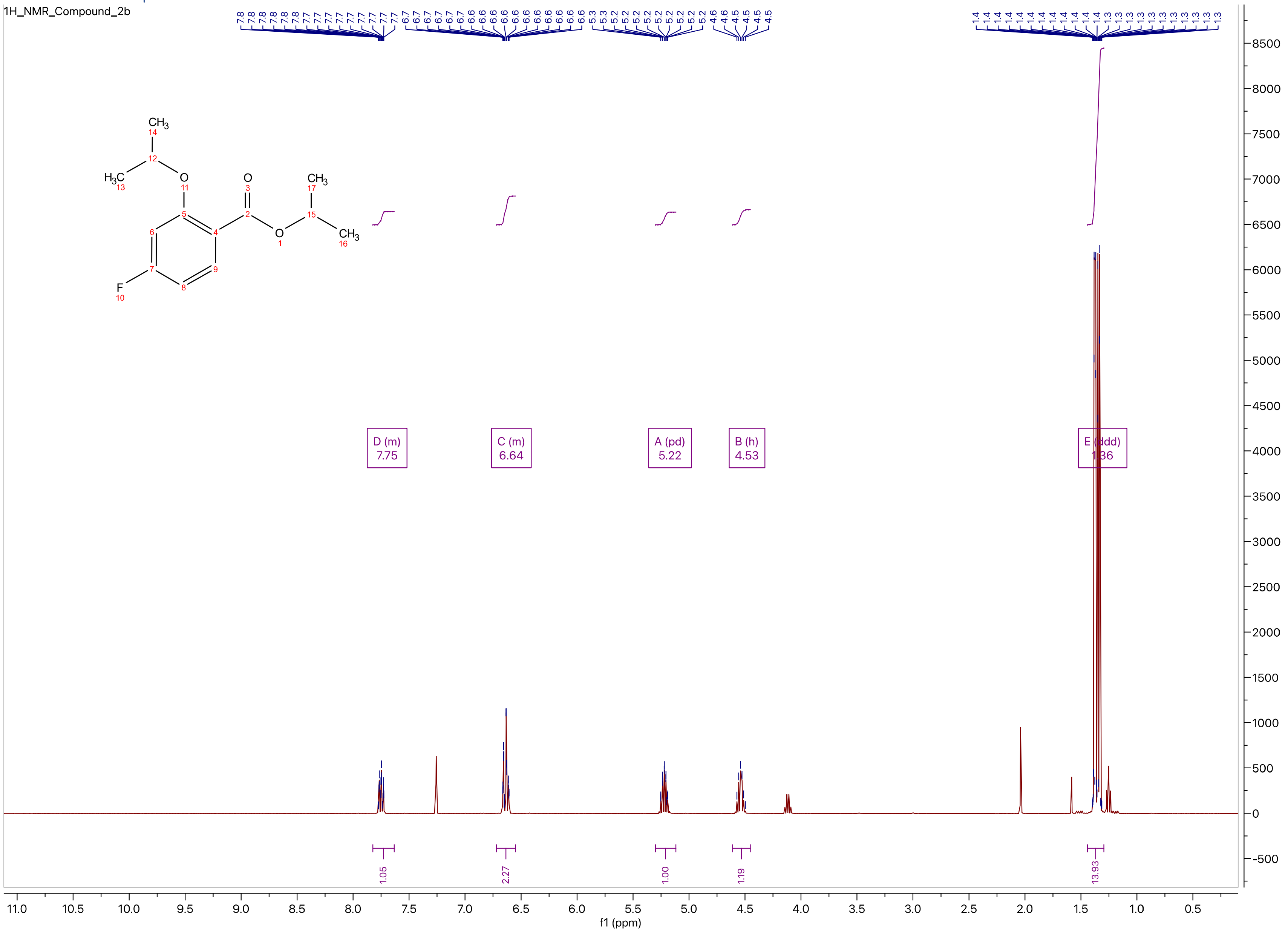

^{13}C NMR Compound **2b**

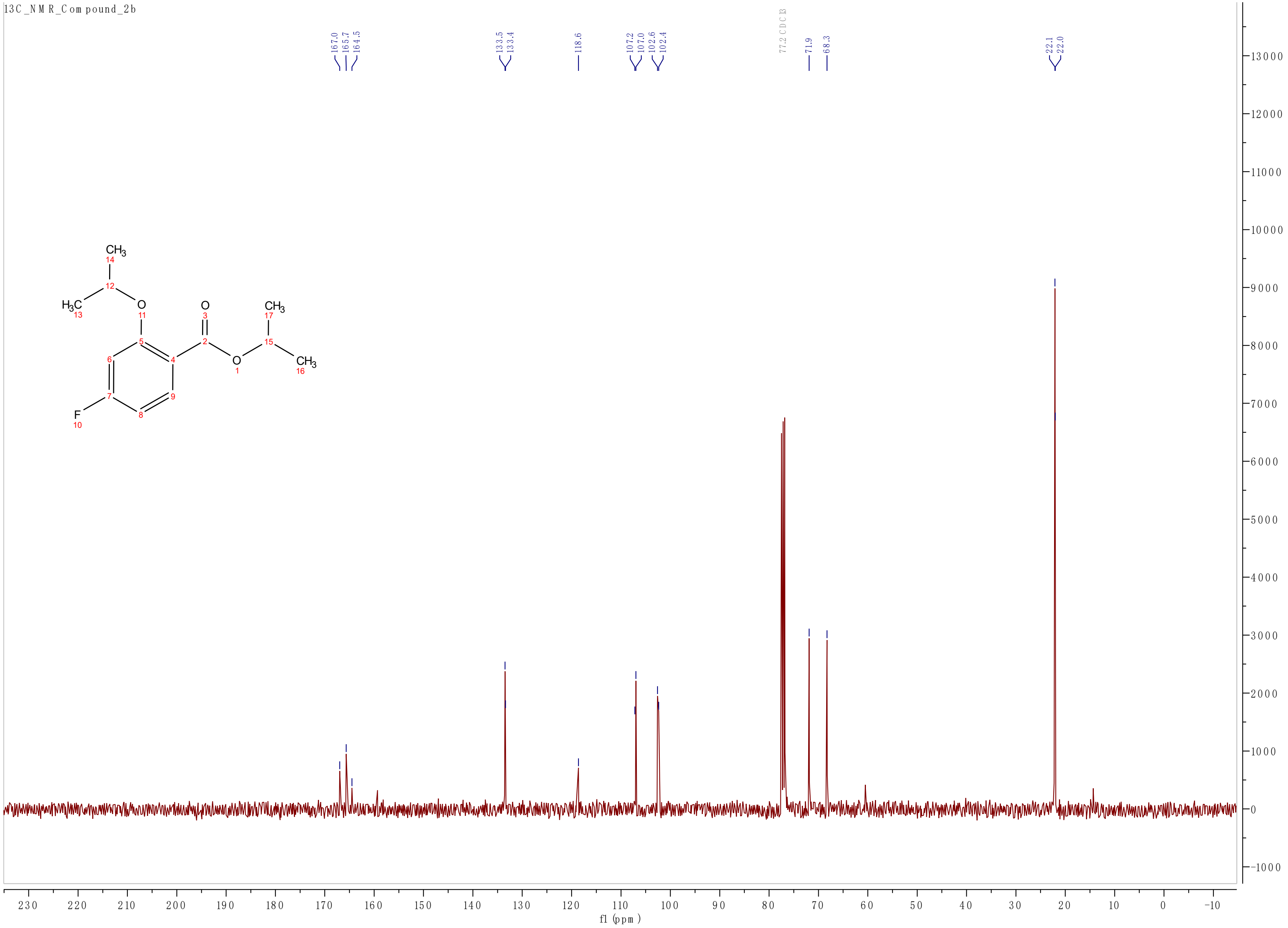

^{1}H NMR Compound **3b**

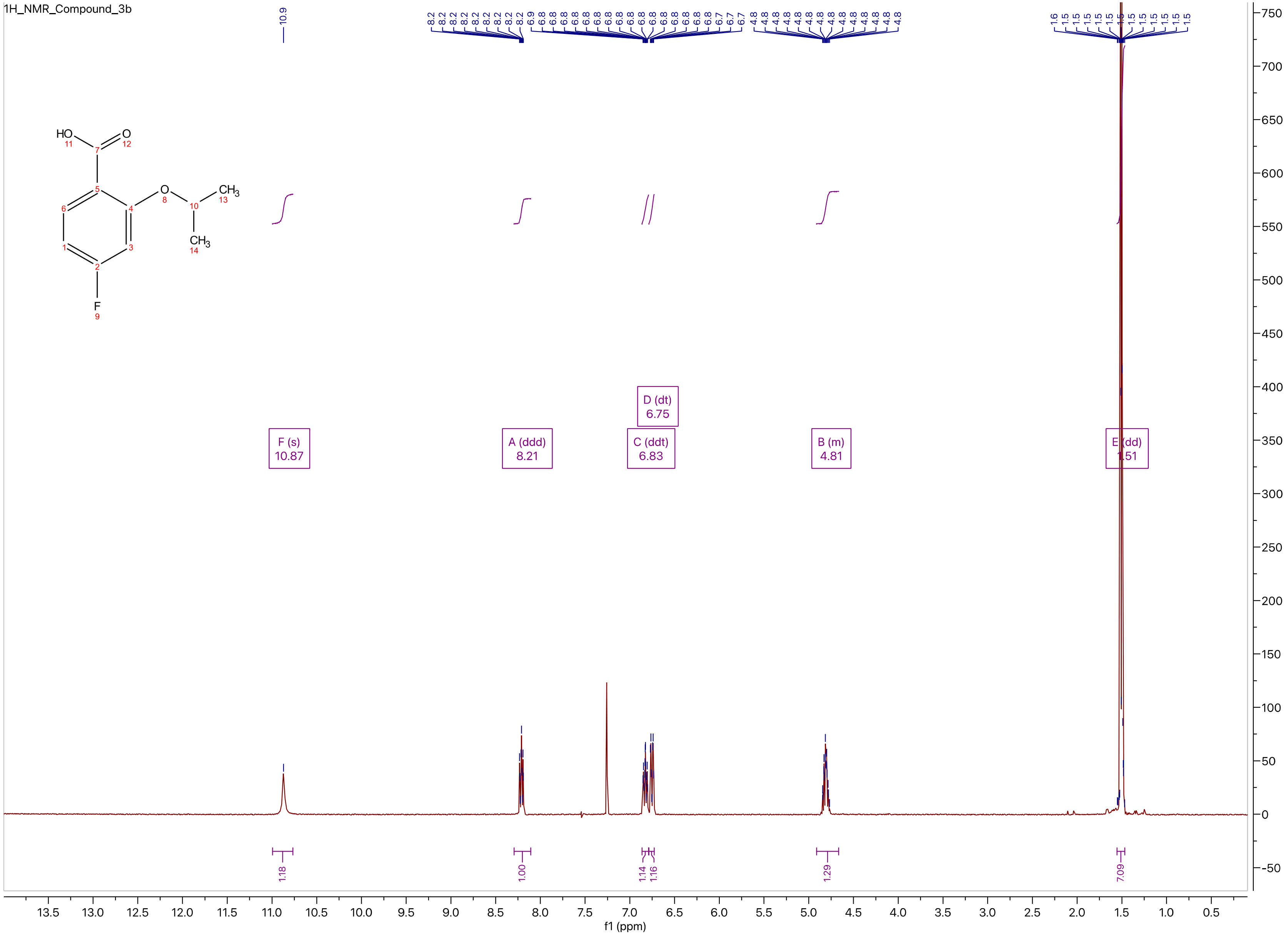

^{13}C NMR Compound **3b**

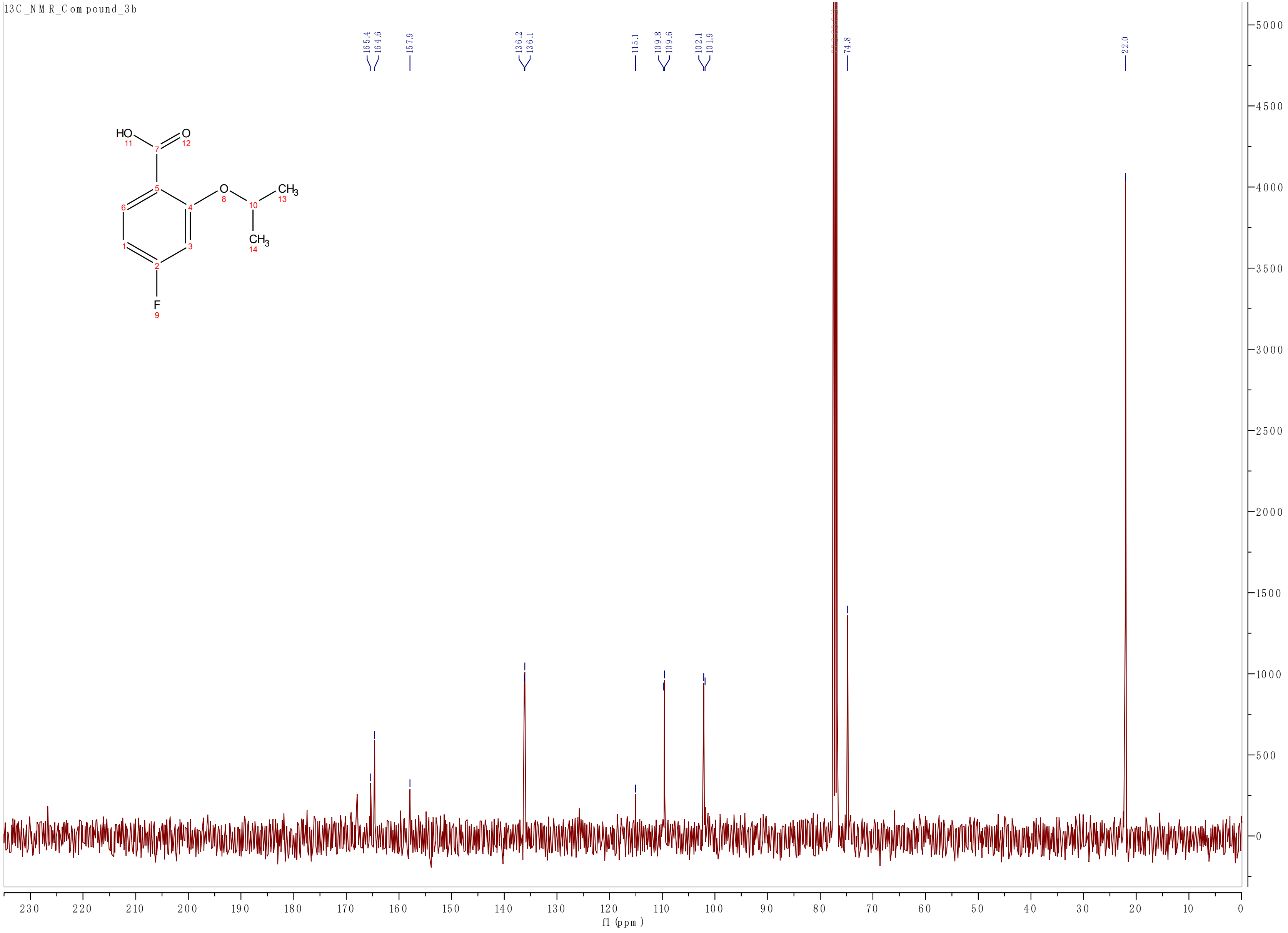

^{19}F NMR Compound **3b**

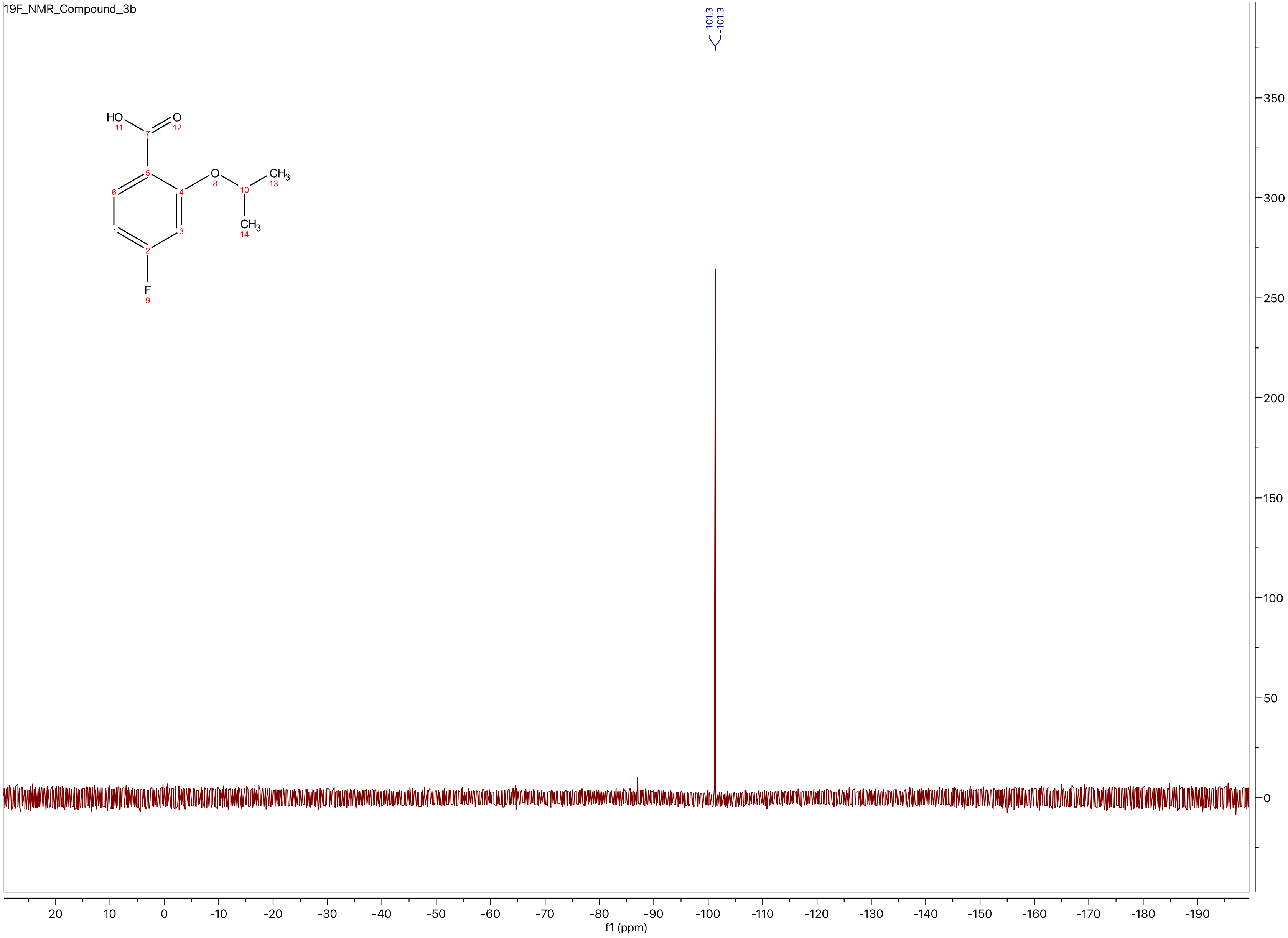

^{1}H NMR Compound *N*-Boc-9

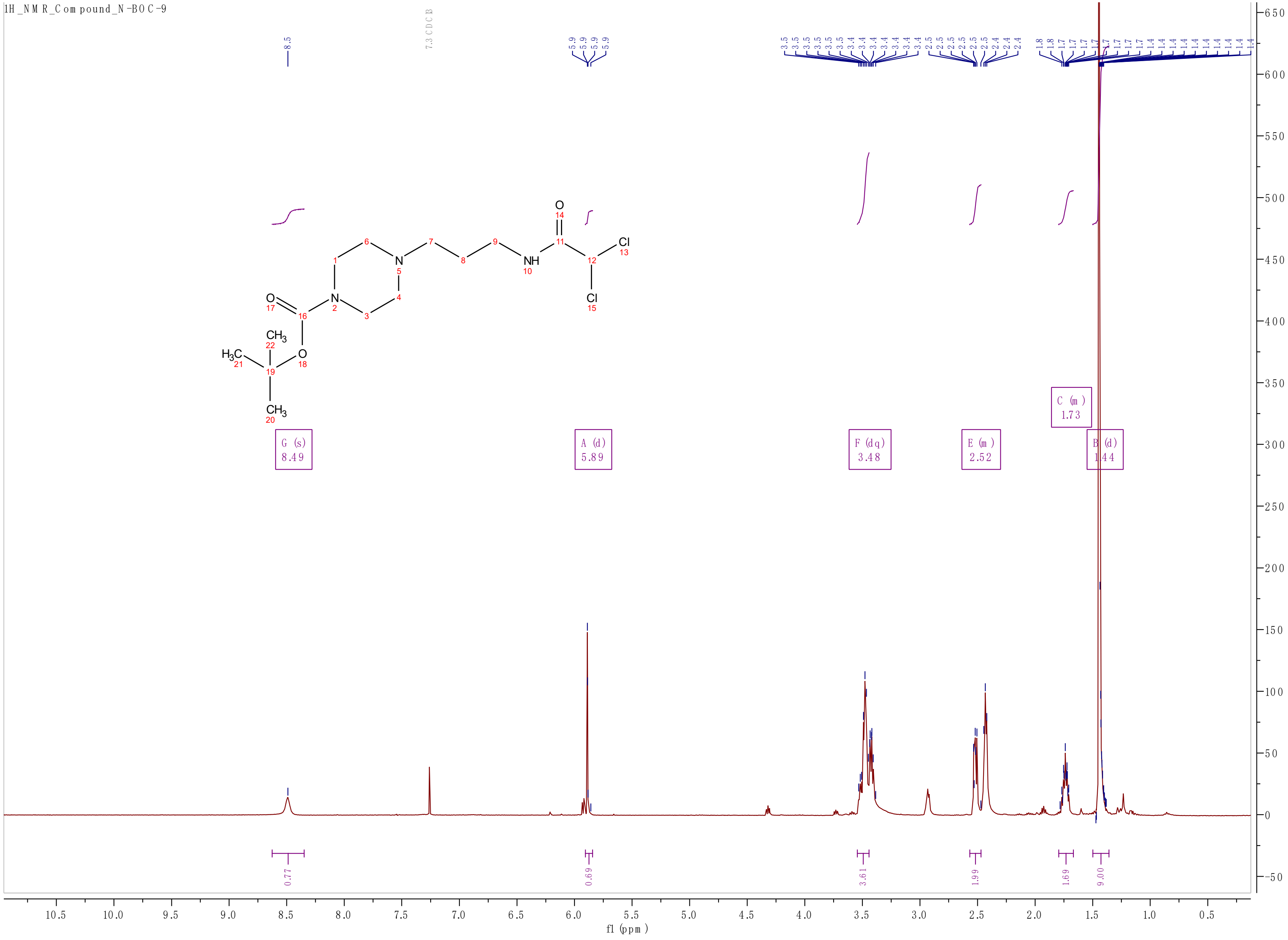

^{13}C NMR Compound *N*-Boc-9

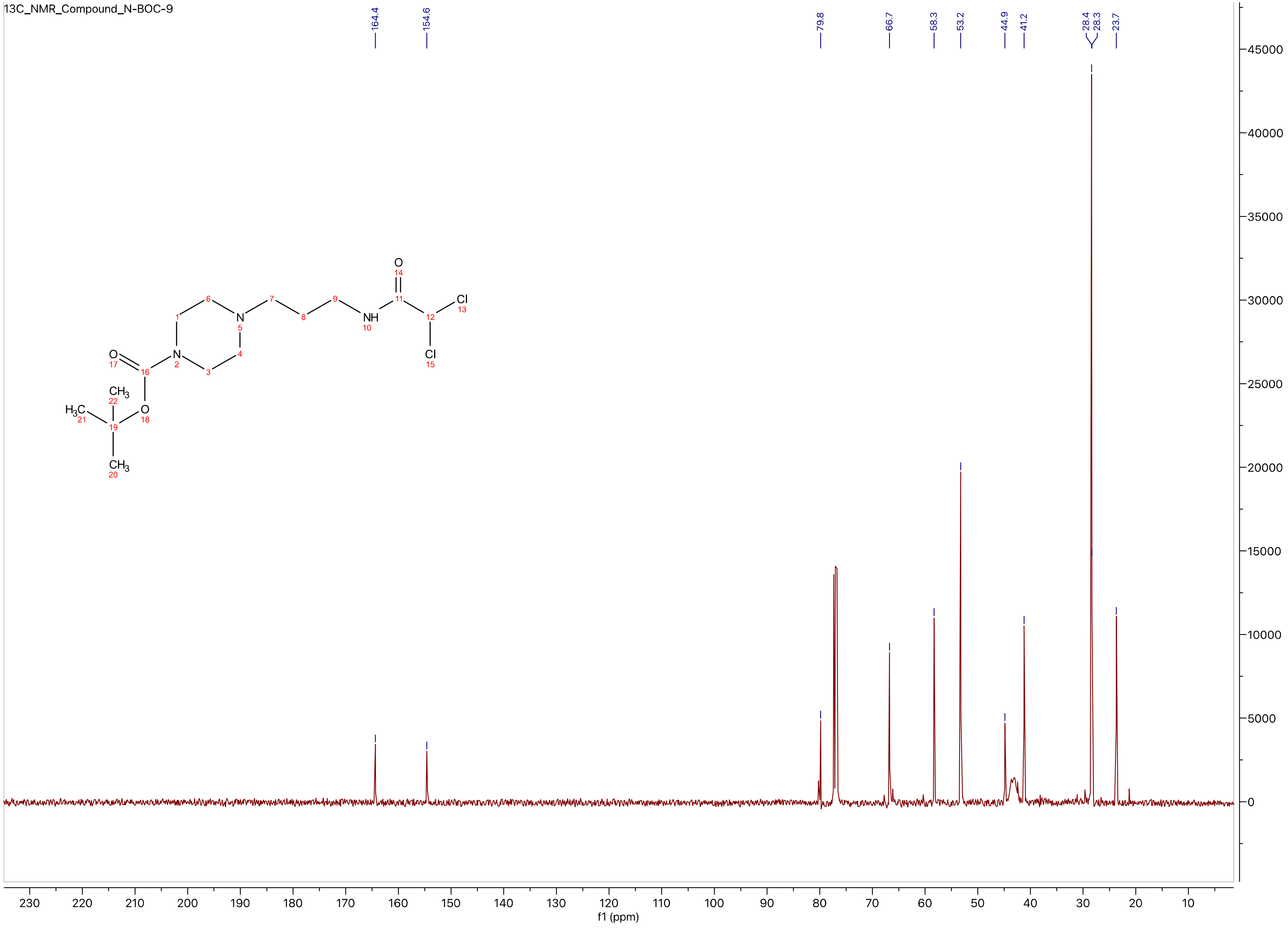

^{1}H NMR Compound 9

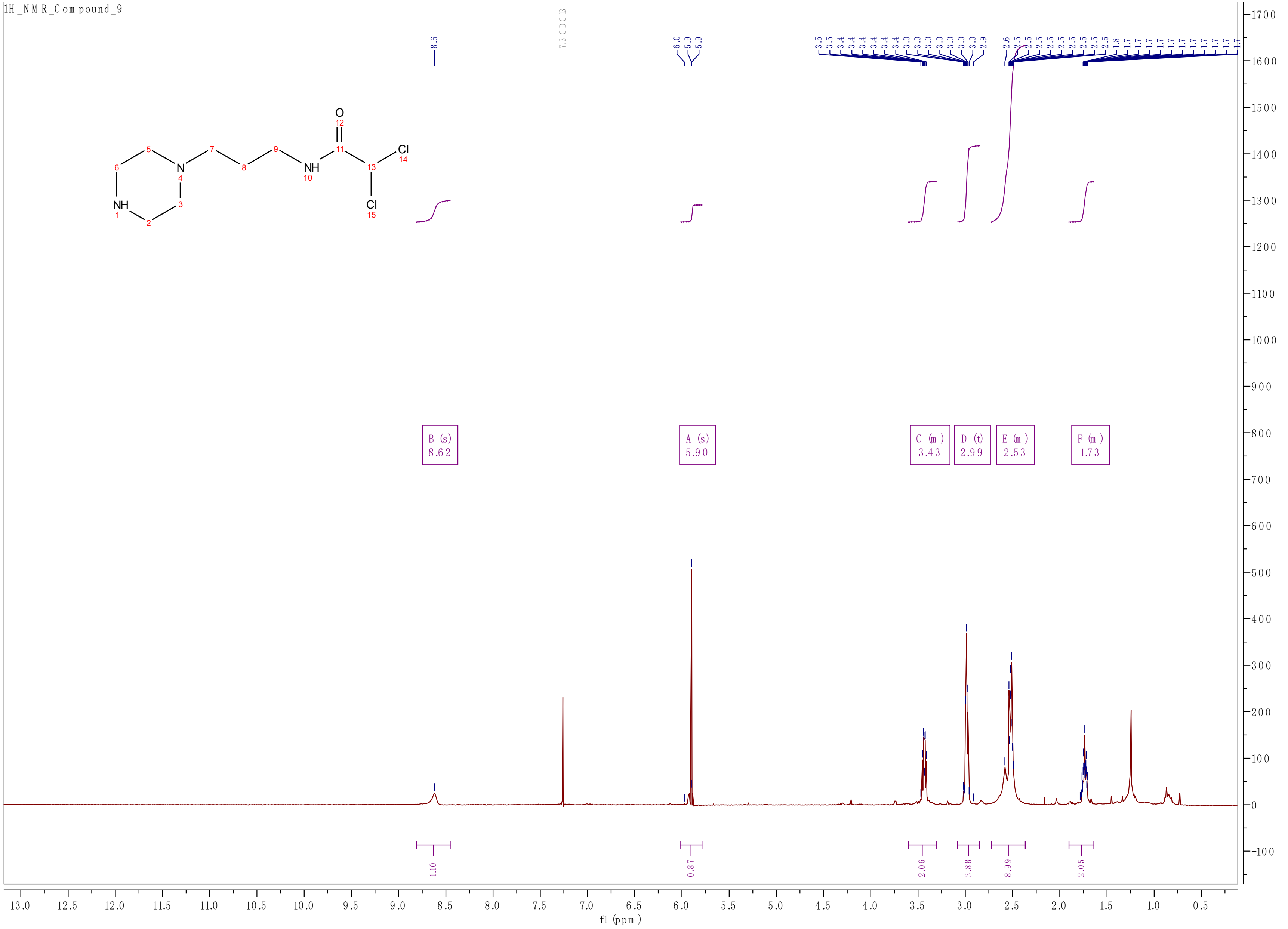

^{13}C NMR Compound 9

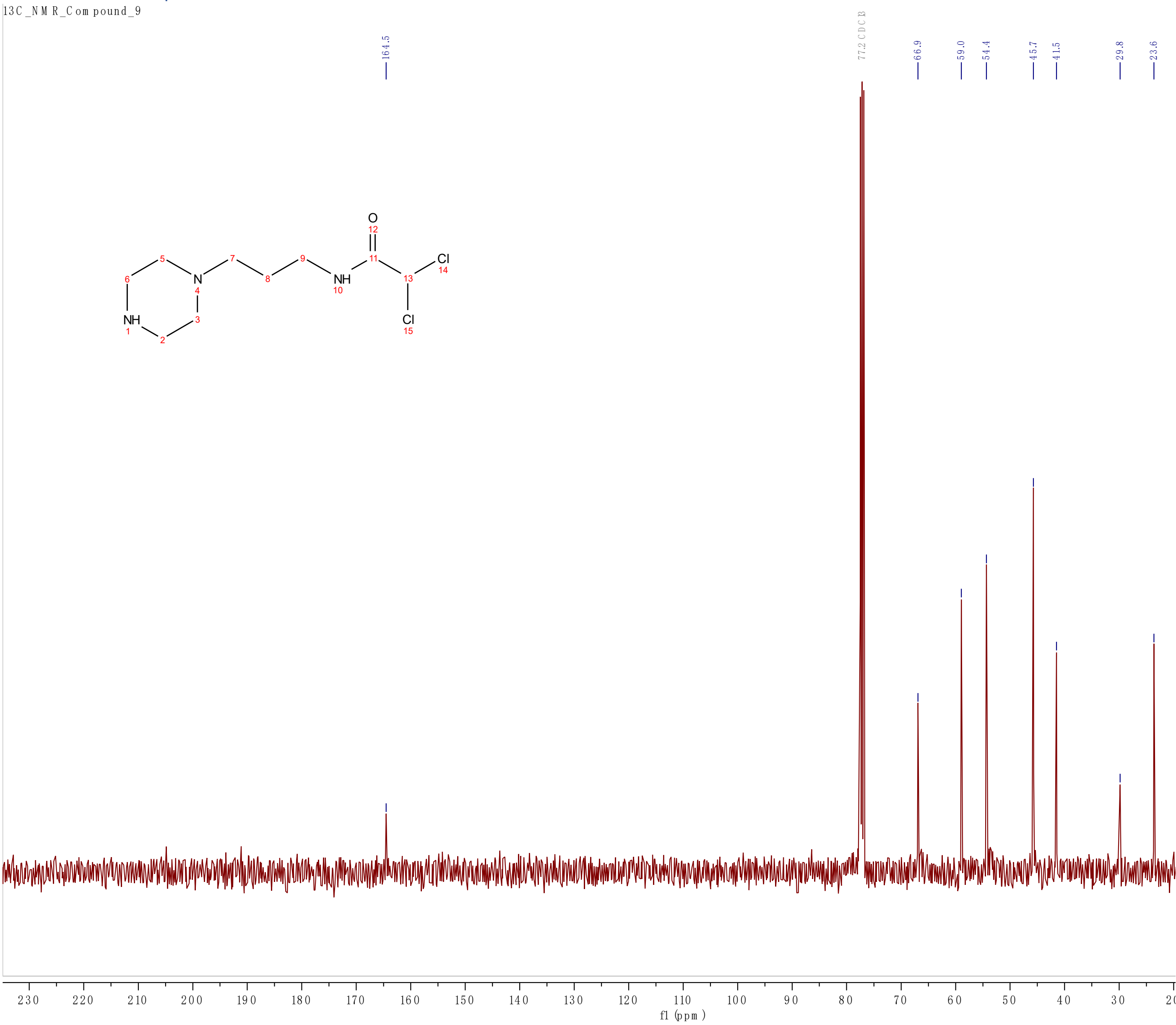

^{1}H NMR Compound **12**

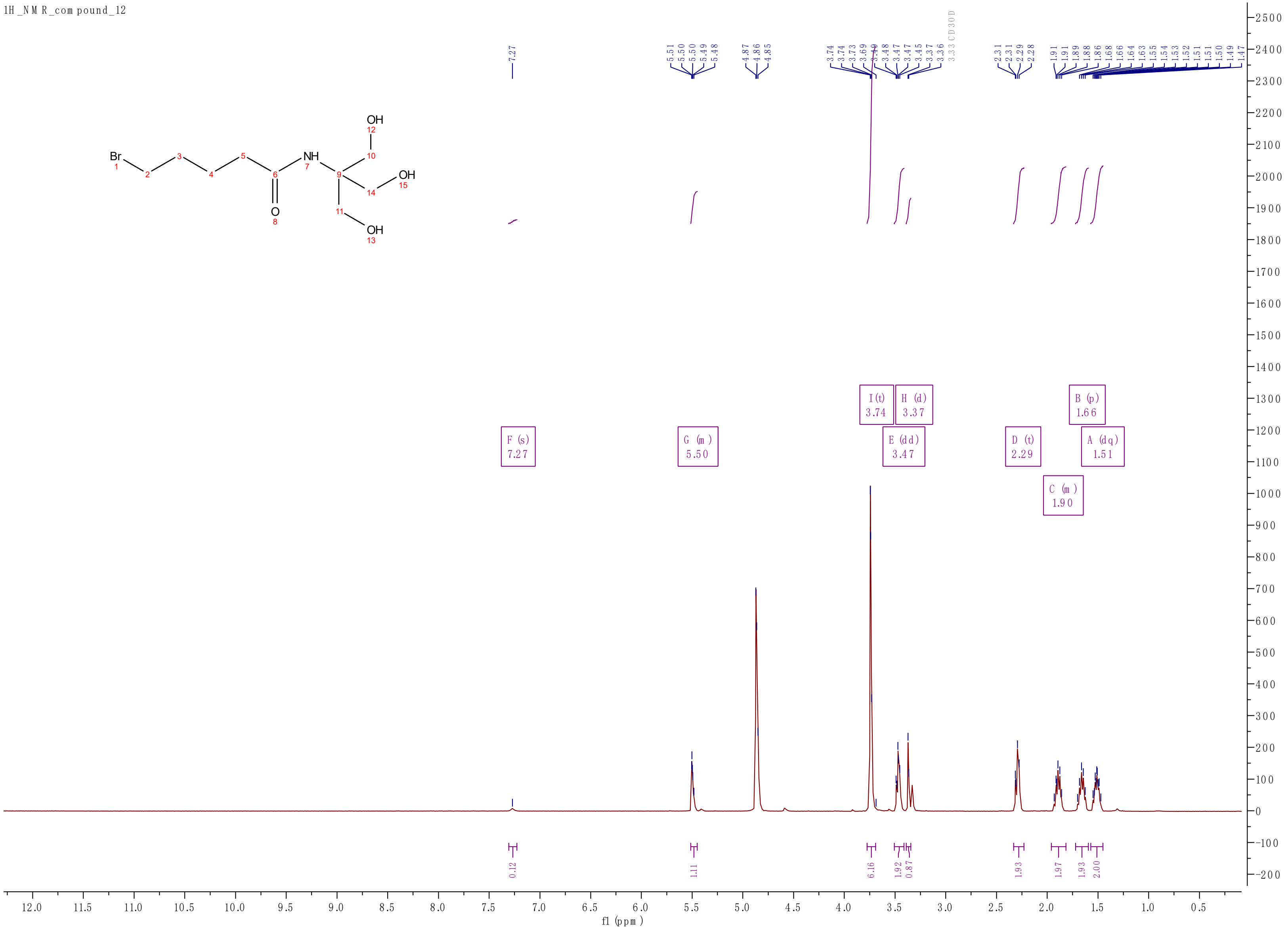

^{13}C NMR Compound 12

13C_NMR_Compound_12

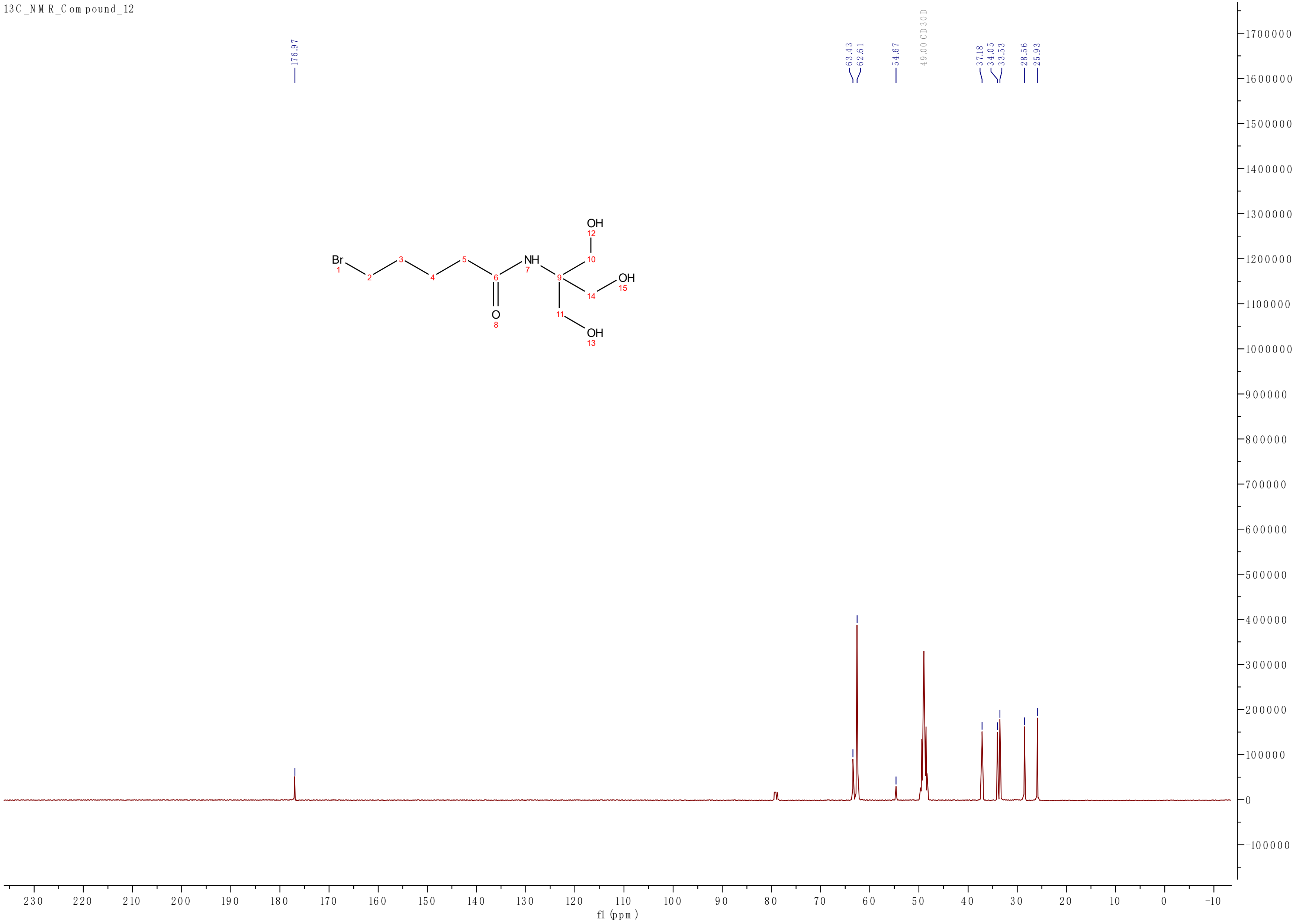

^{1}H NMR Compound 13

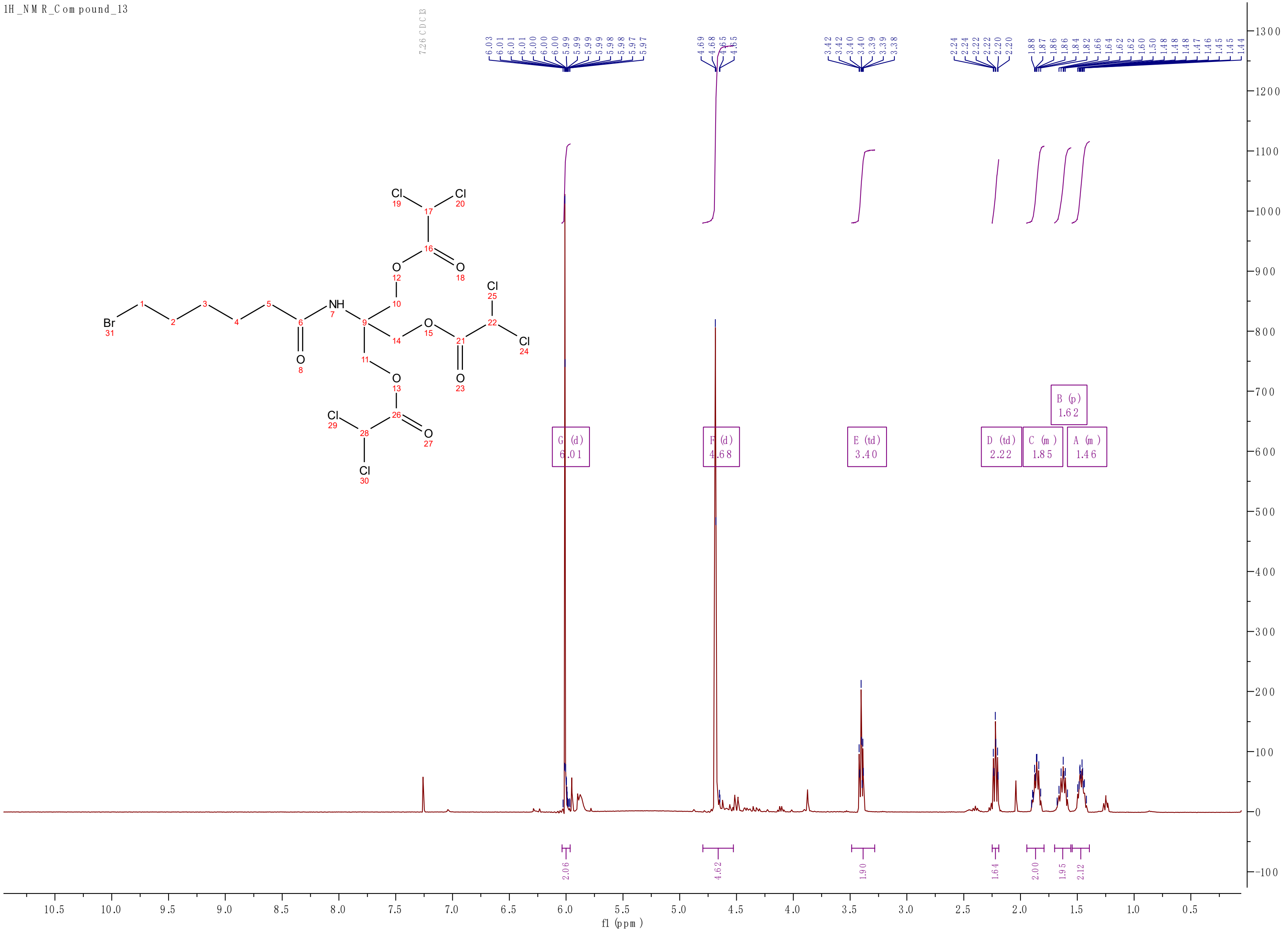

^{13}C NMR Compound 13

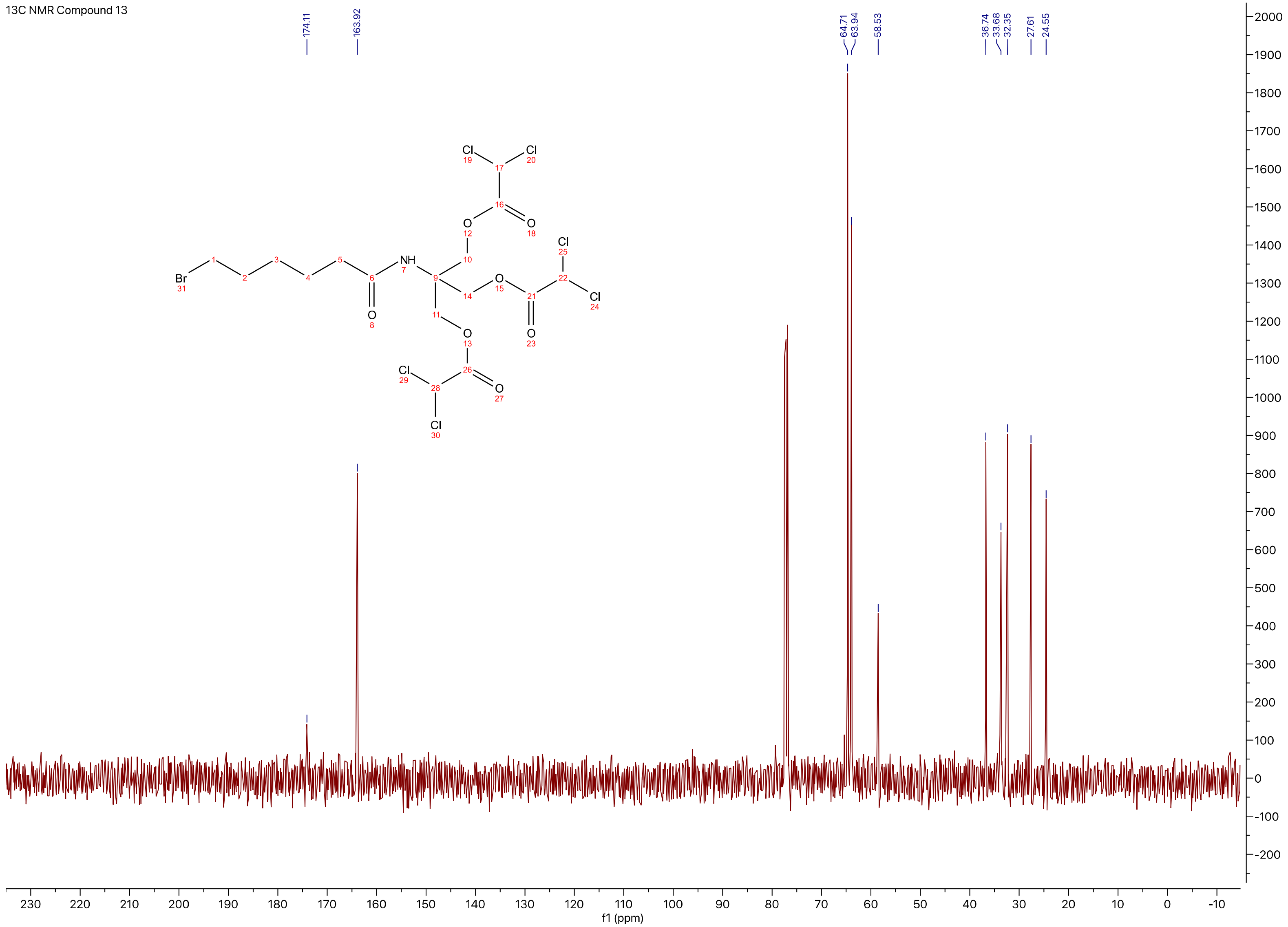

^{1}H NMR Compound **16a**

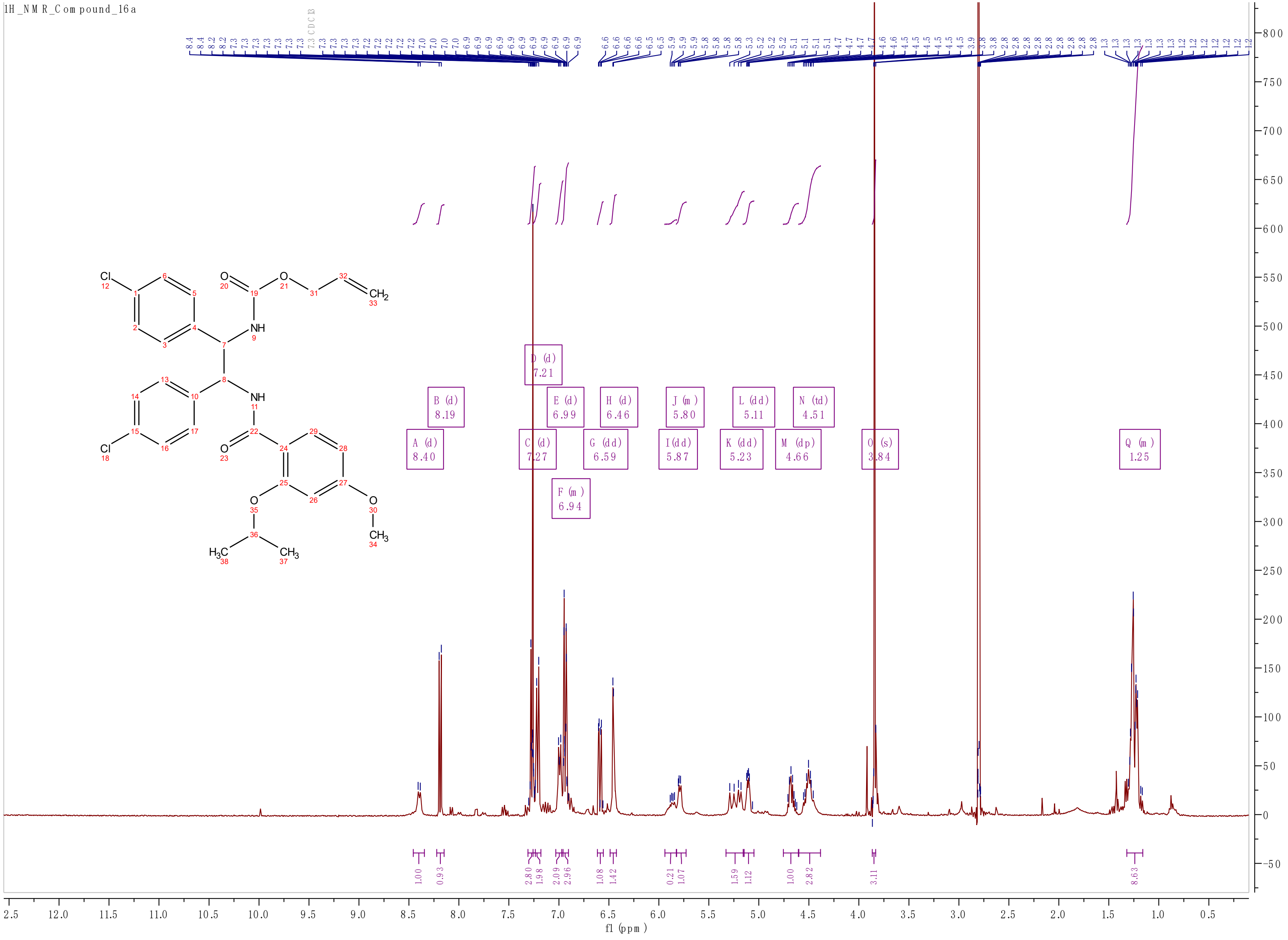

^{13}C NMR Compound **16a**

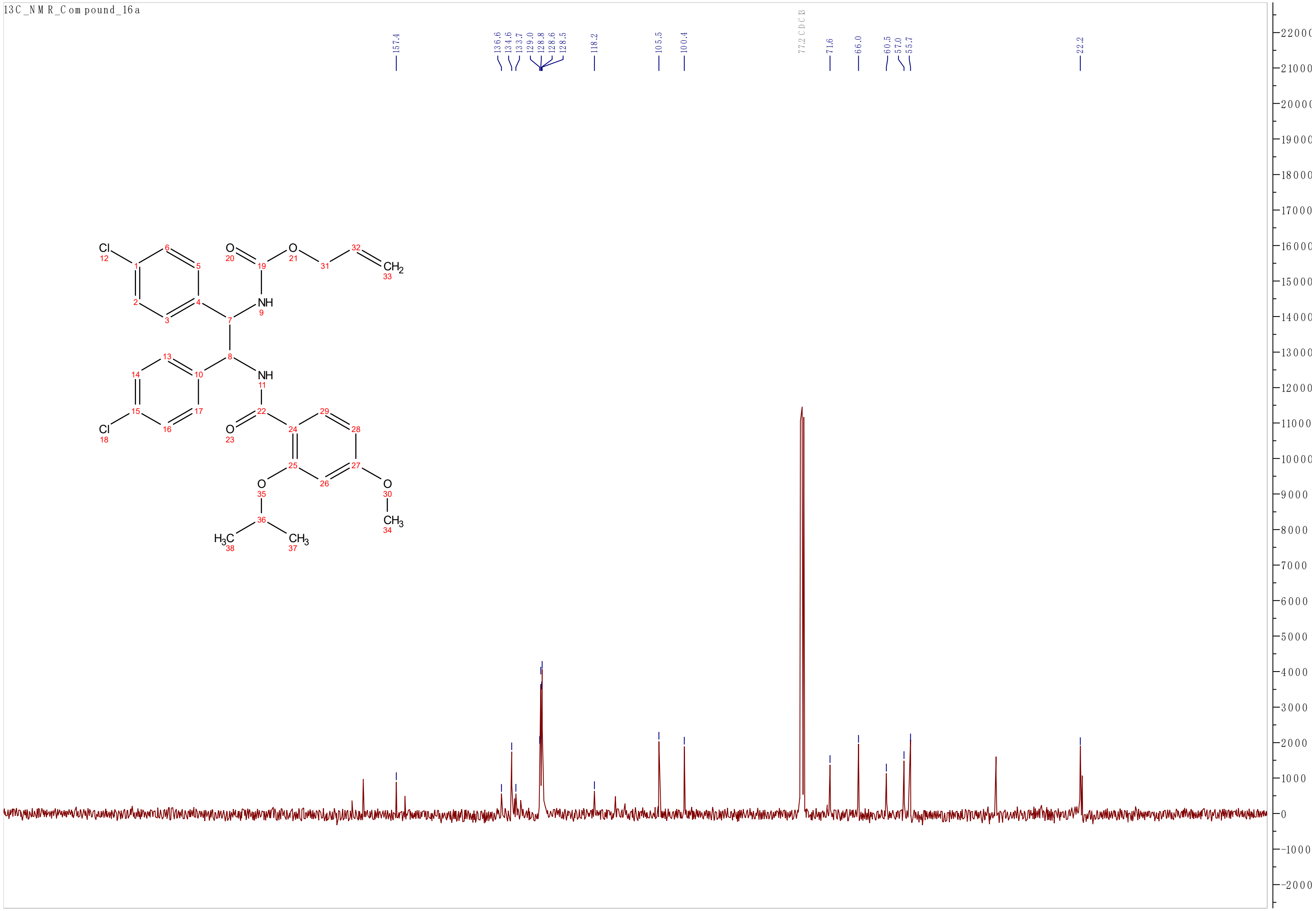

^{1}H NMR Compound **16b**

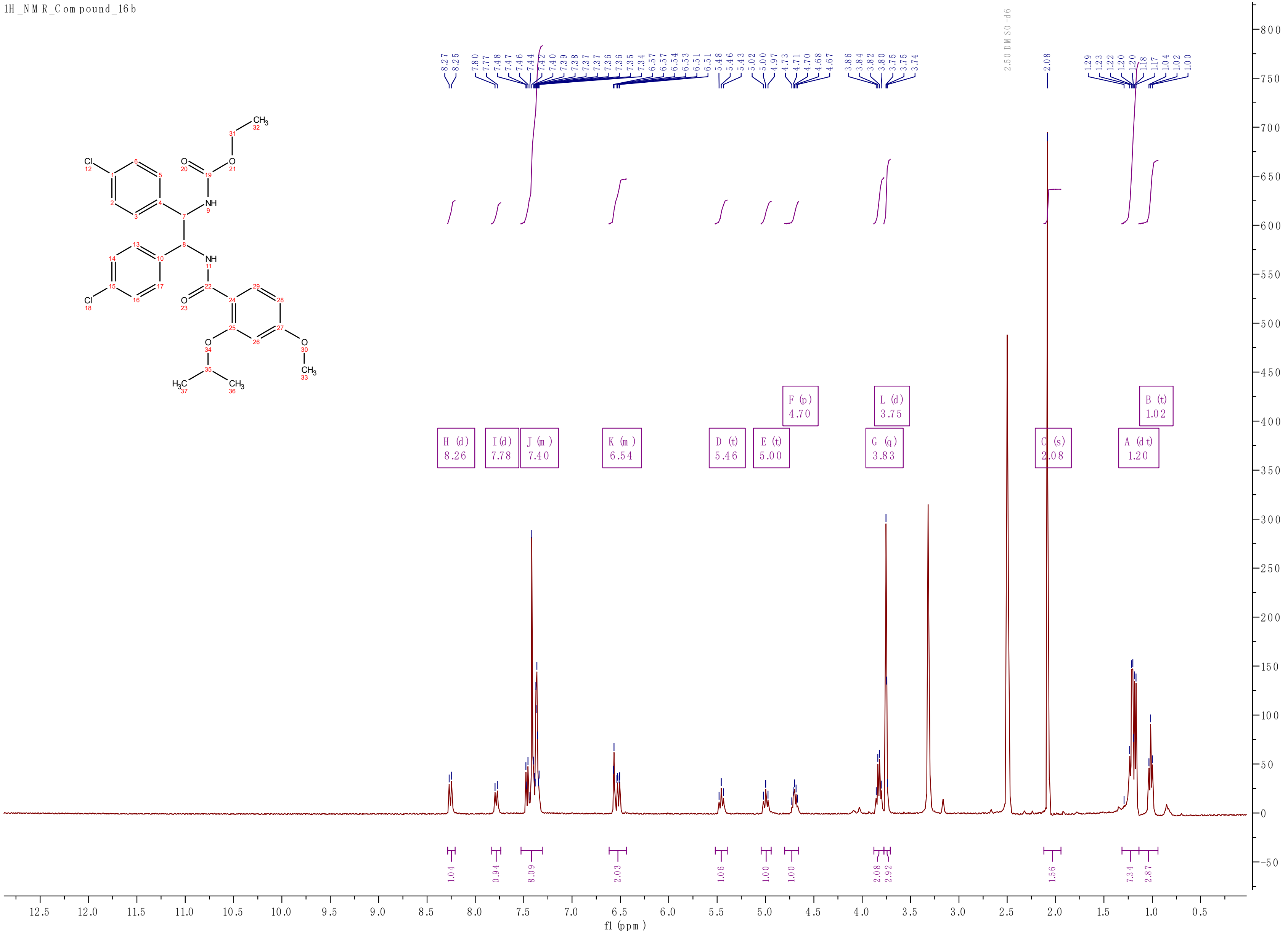

^{13}C NMR Compound **16b**

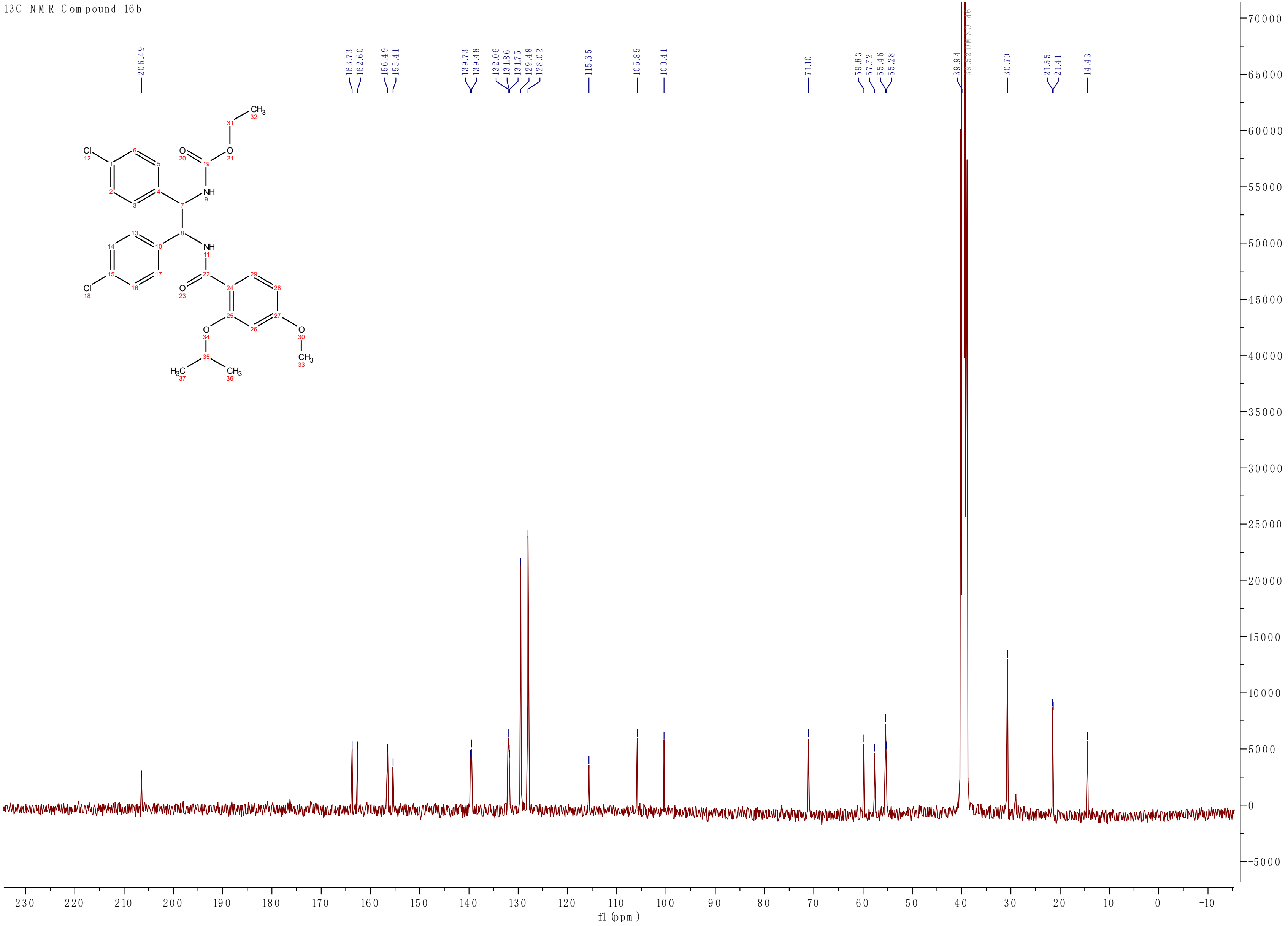

^{1}H NMR Compound **16c**

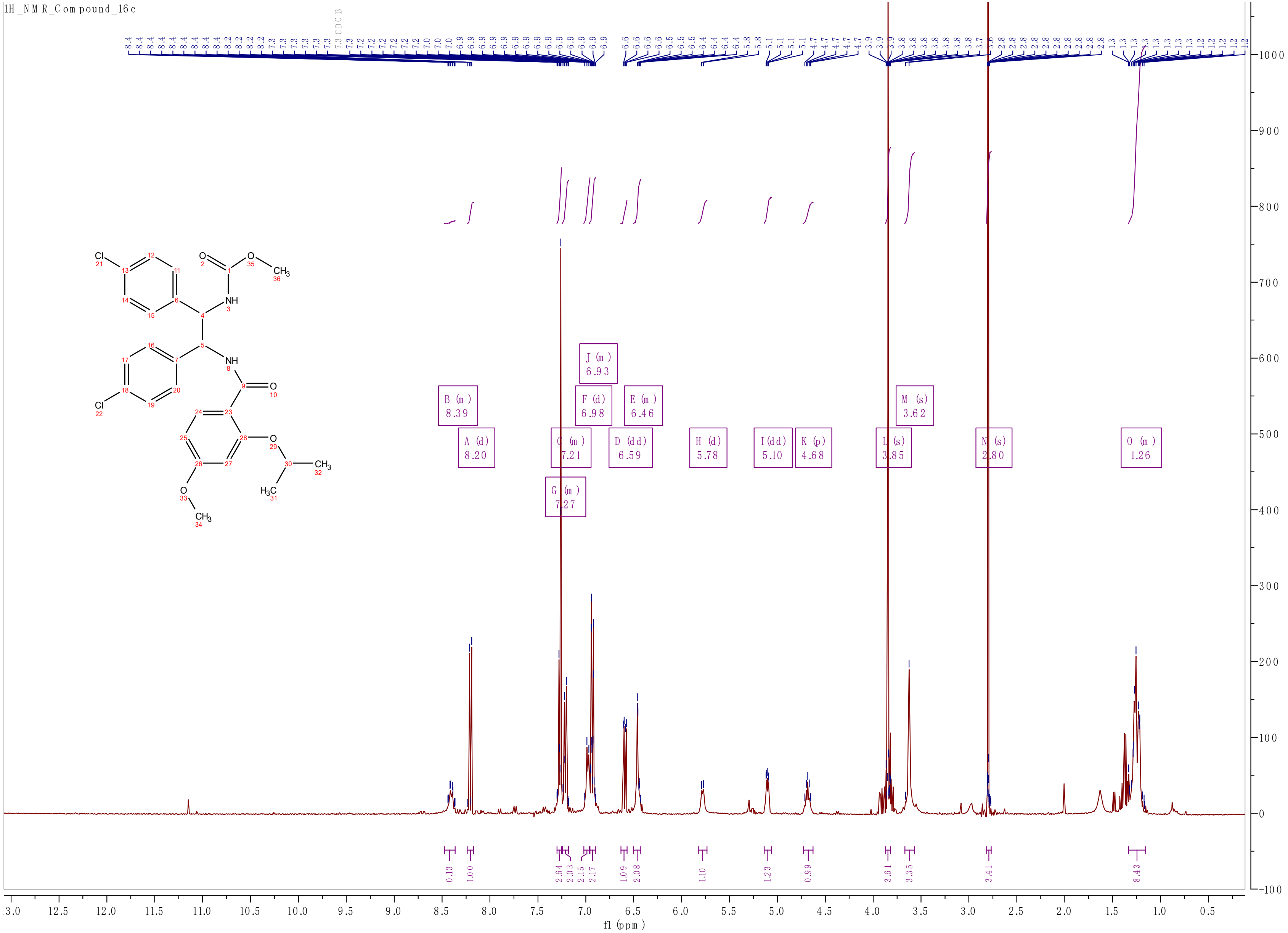

^{13}C NMR Compound **16c**

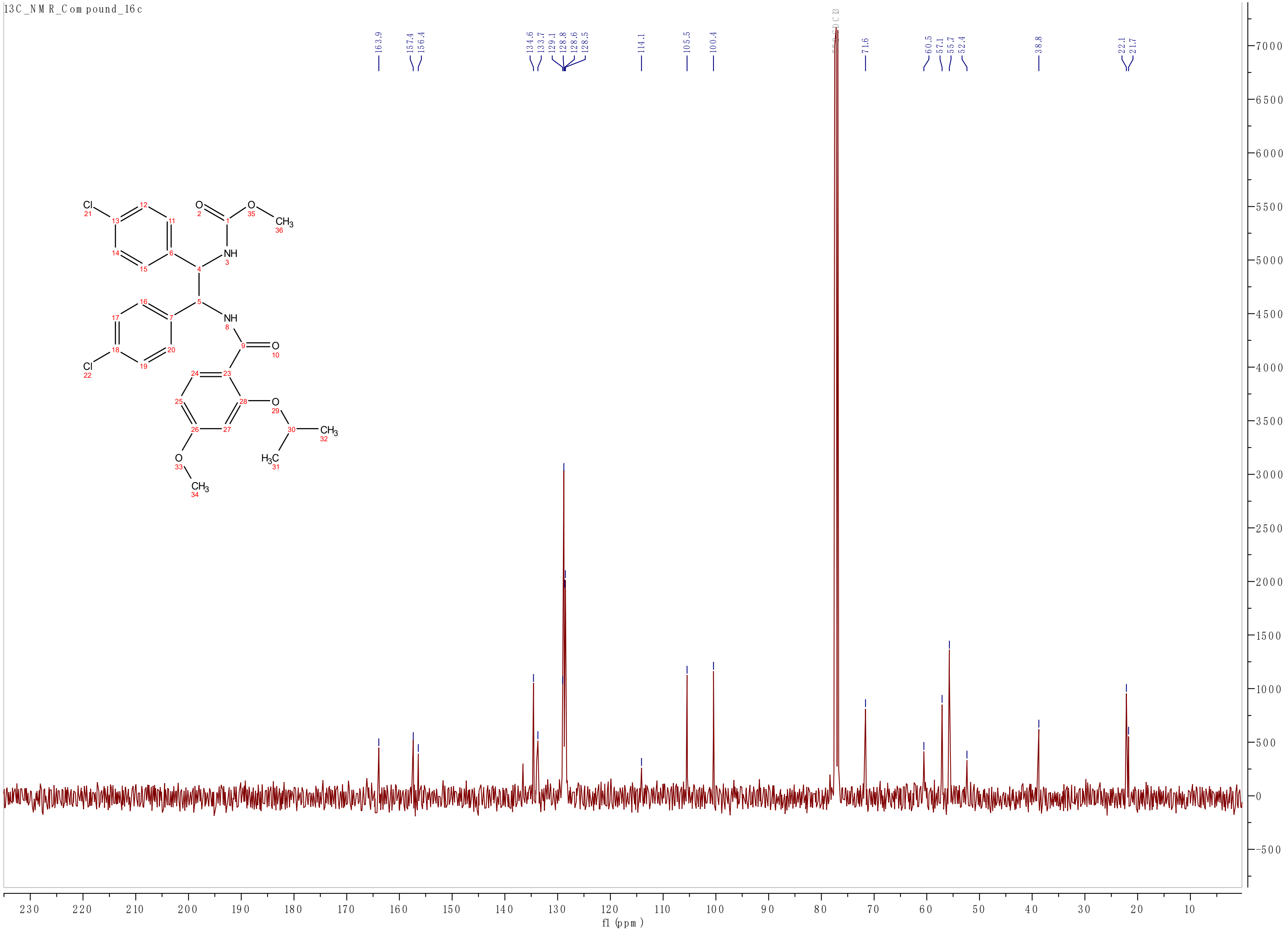

^{1}H NMR Compound **16d**

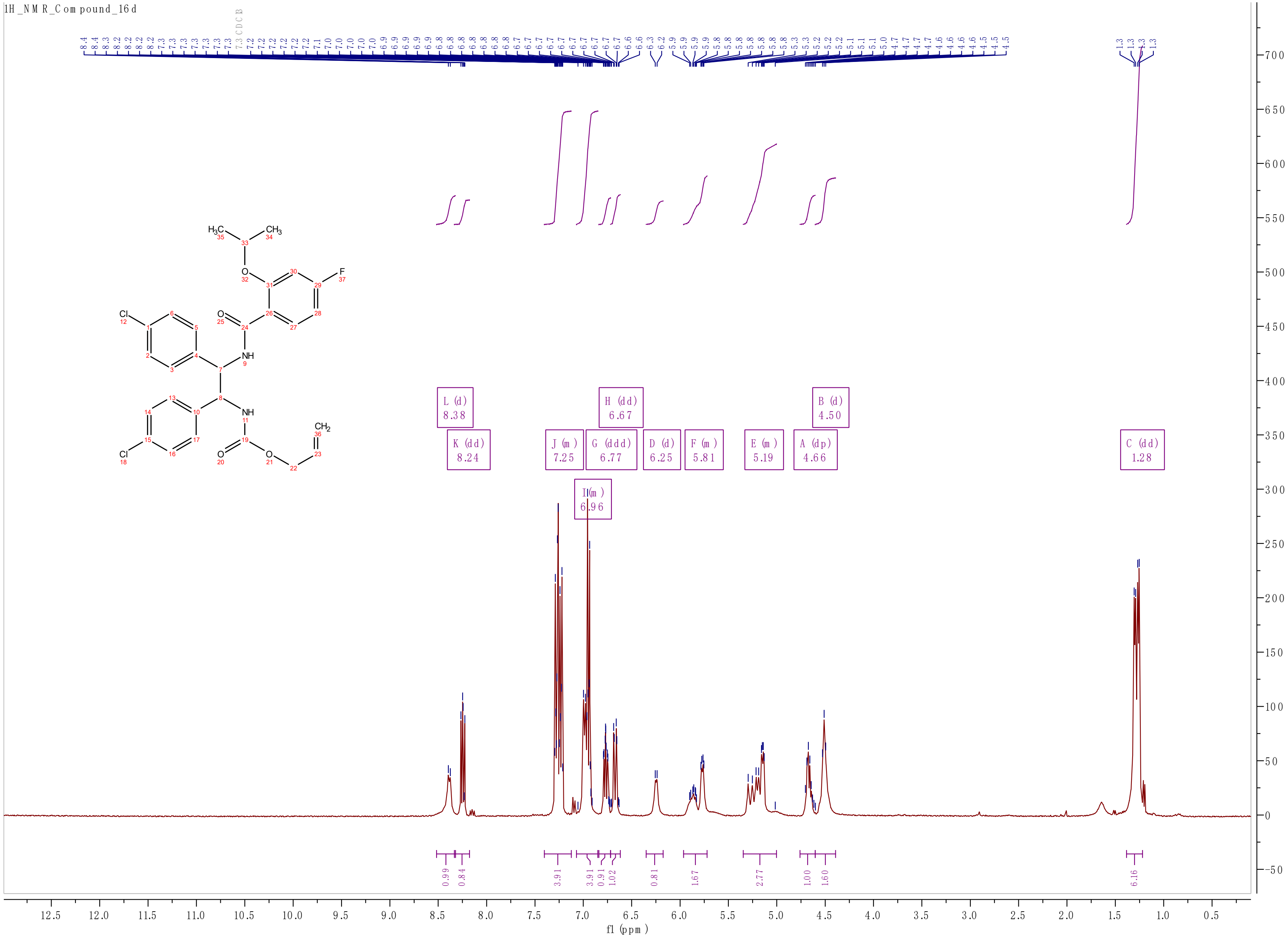

^{13}C NMR Compound 16d

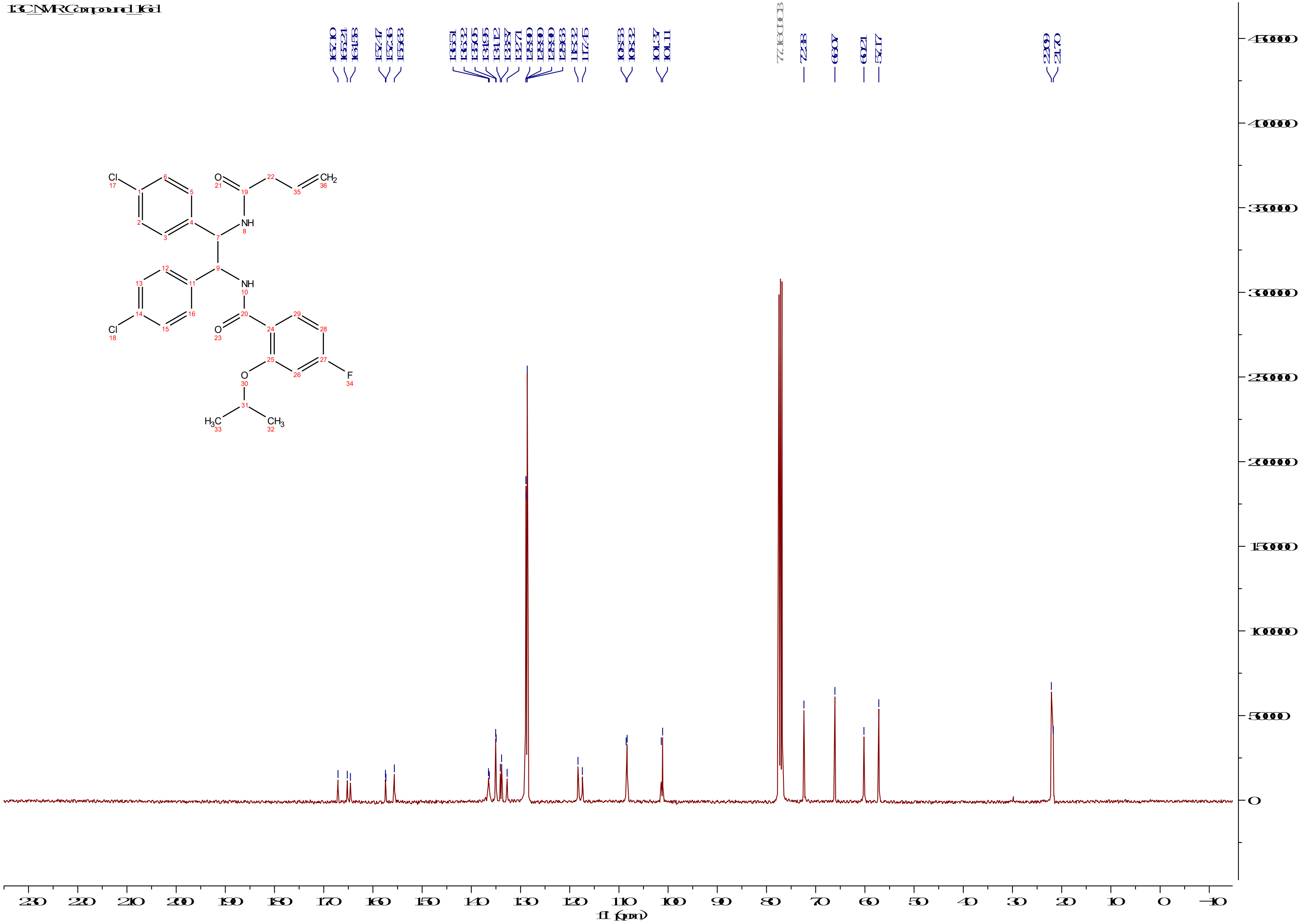

^{19}F NMR Compound **16d**

19F_NMR_Compound_16d

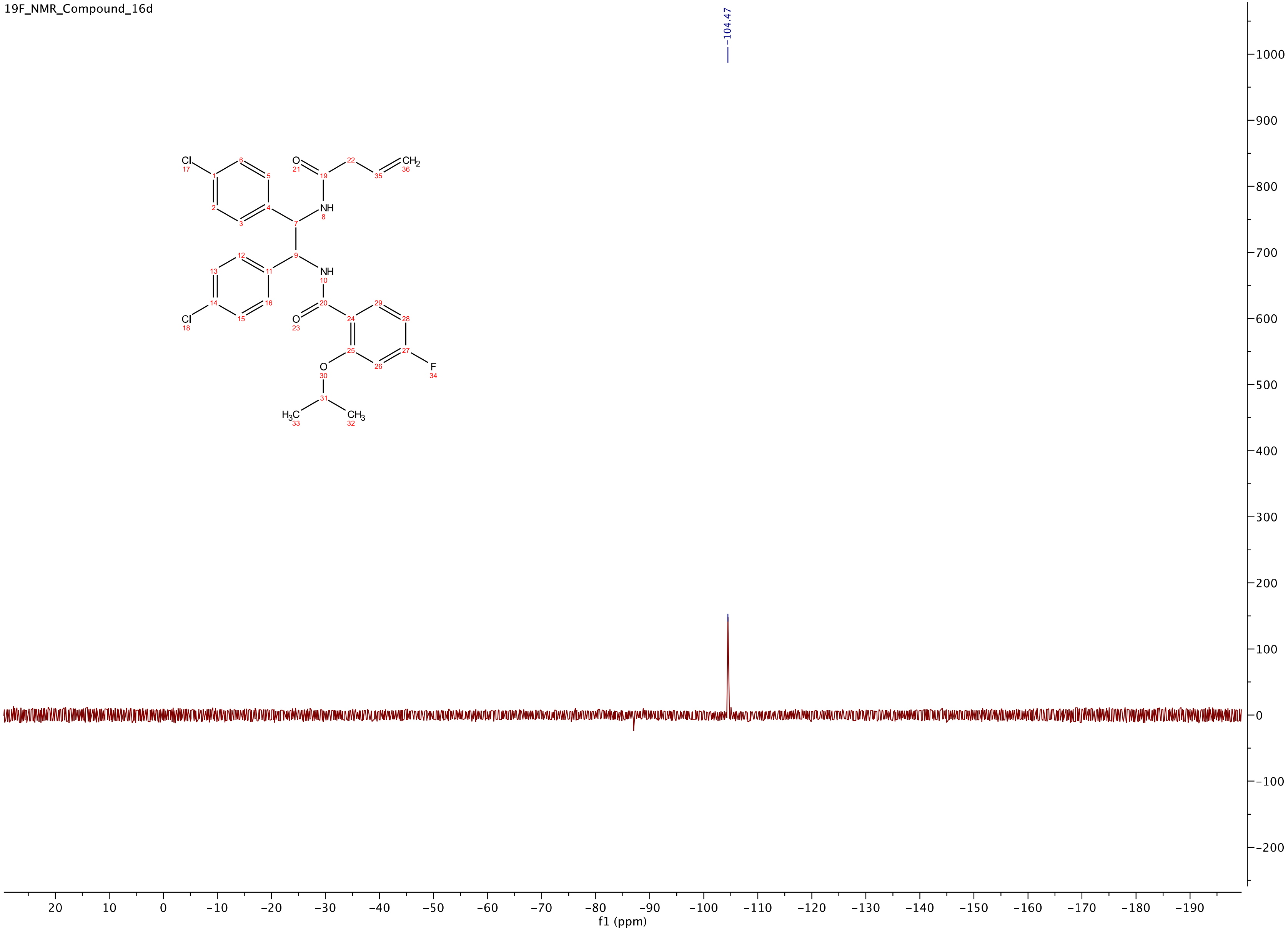

^{1}H NMR Compound **17b**

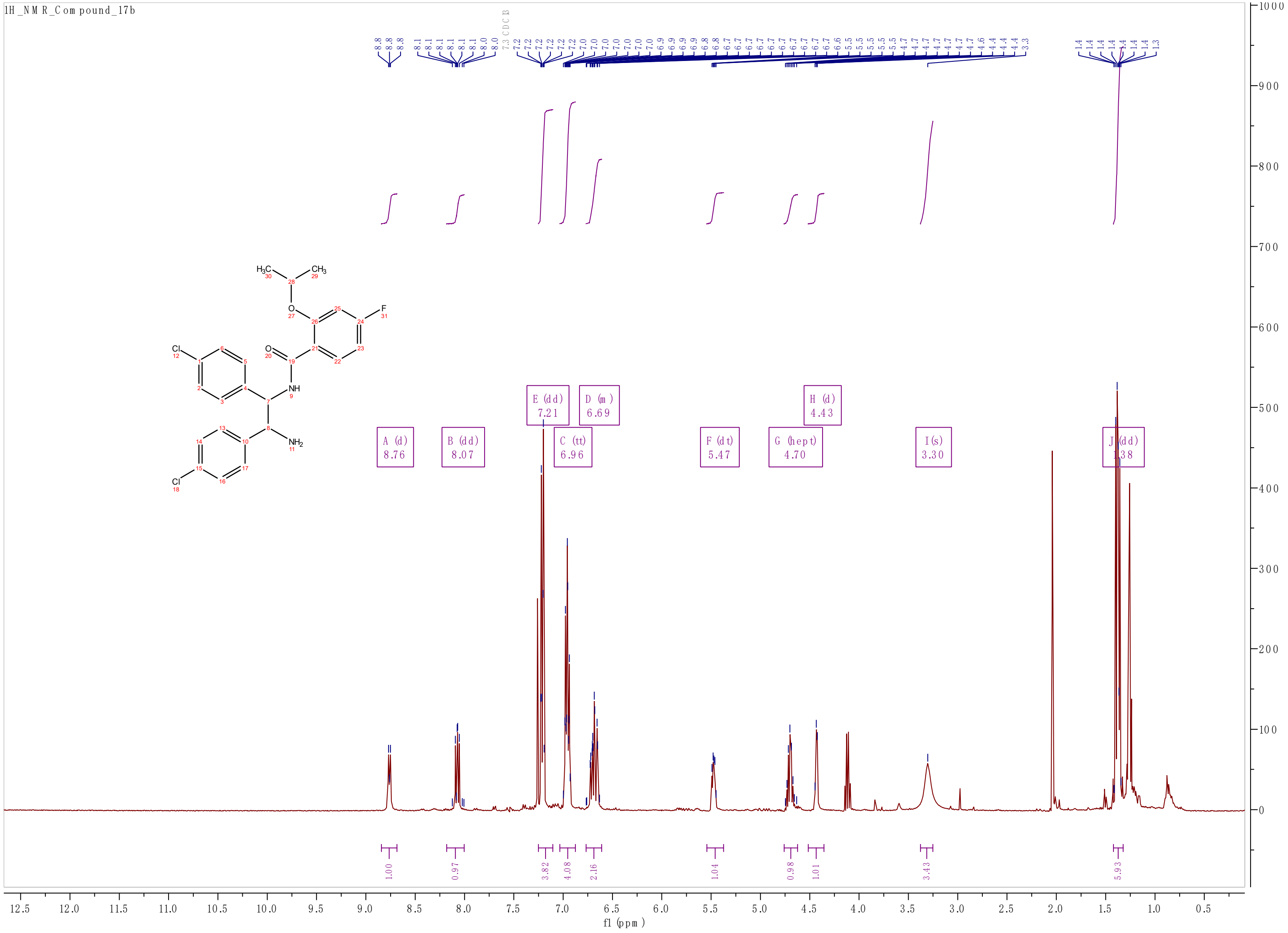

^{13}C NMR Compound **17b**

13C_NMR_Compound_17b

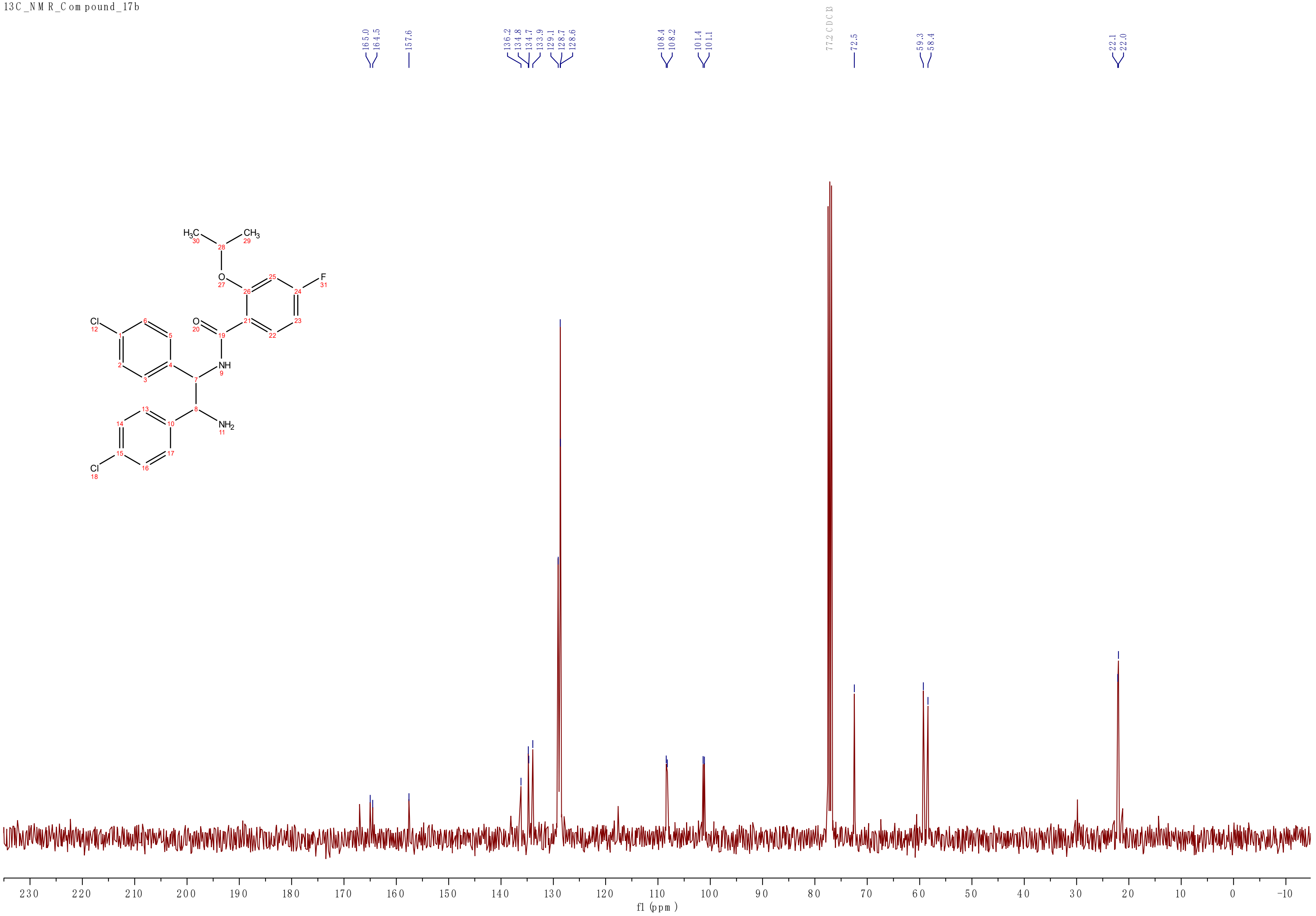

^{1}H NMR Compound 18a

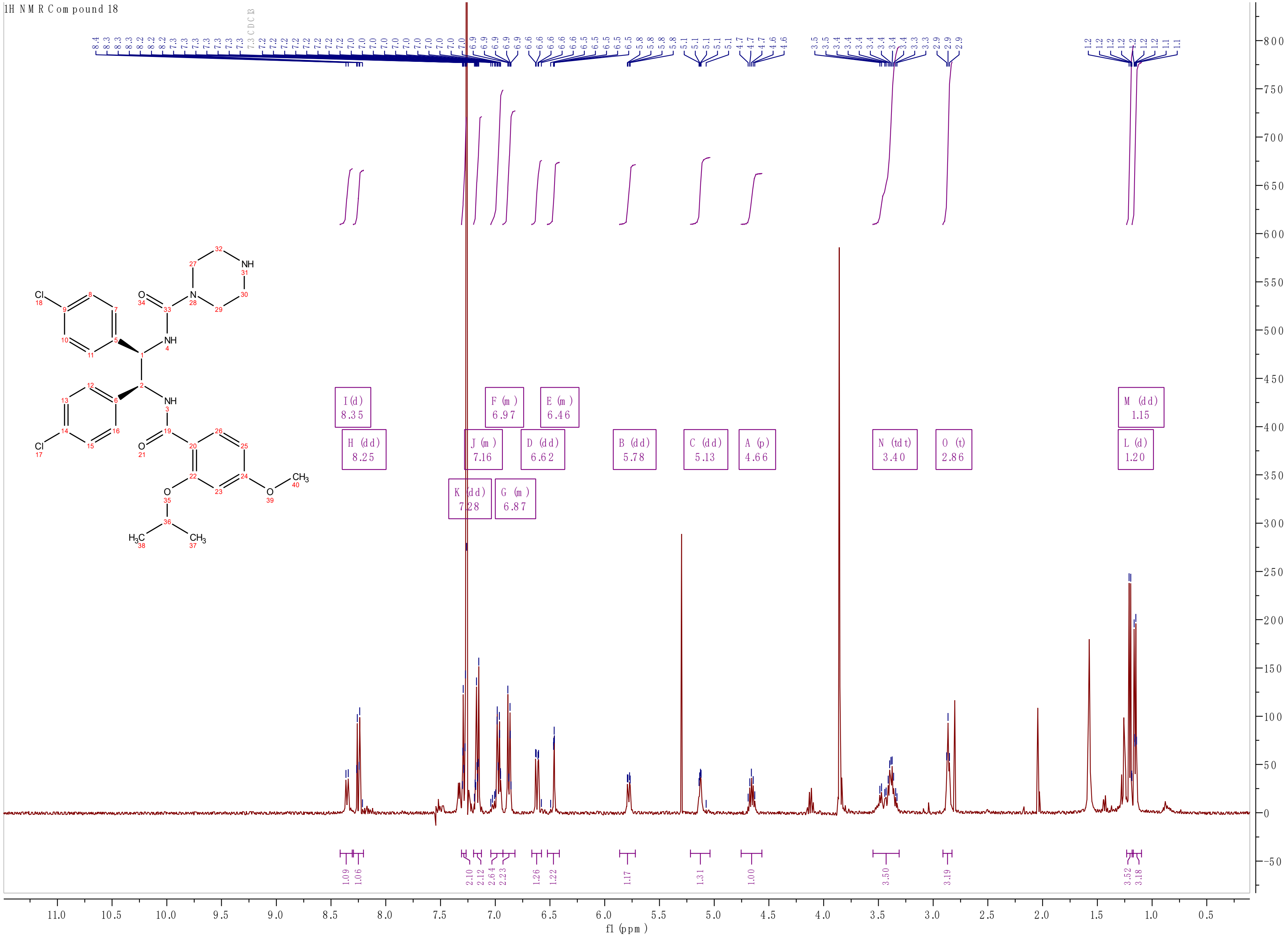

^{13}C NMR Compound **18a**

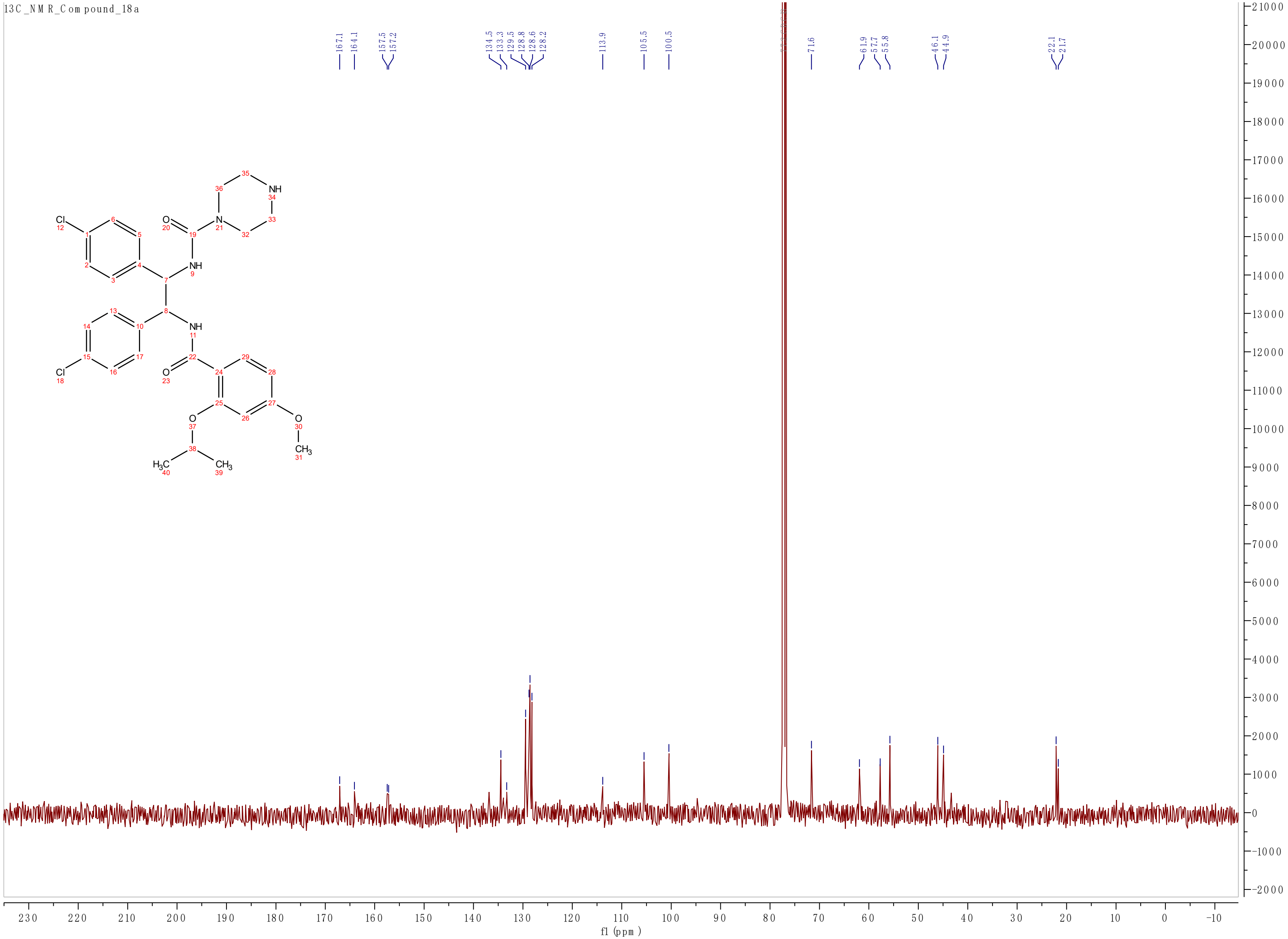

^{1}H NMR Compound **18b**

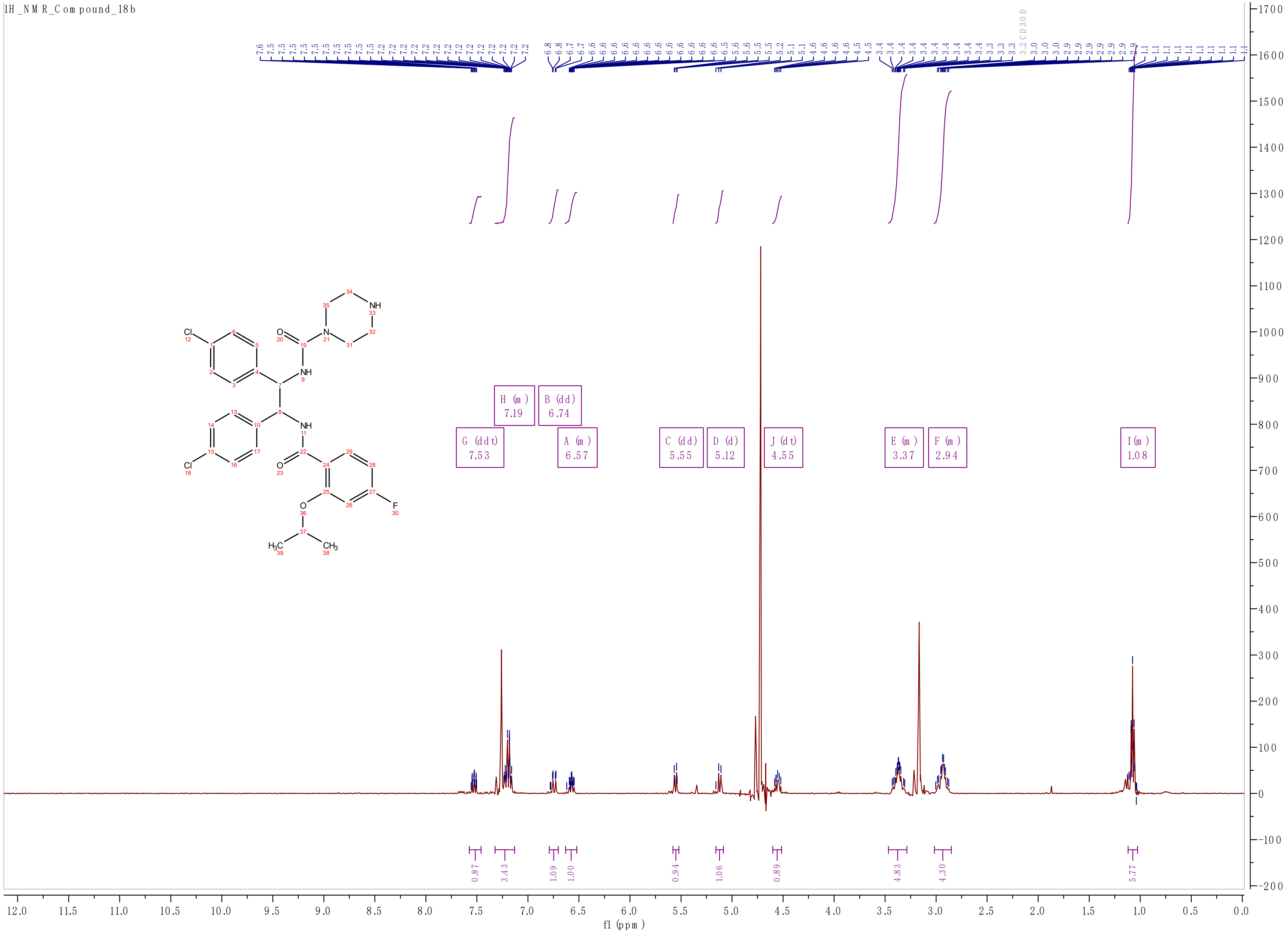

^{13}C NMR Compound **18b**

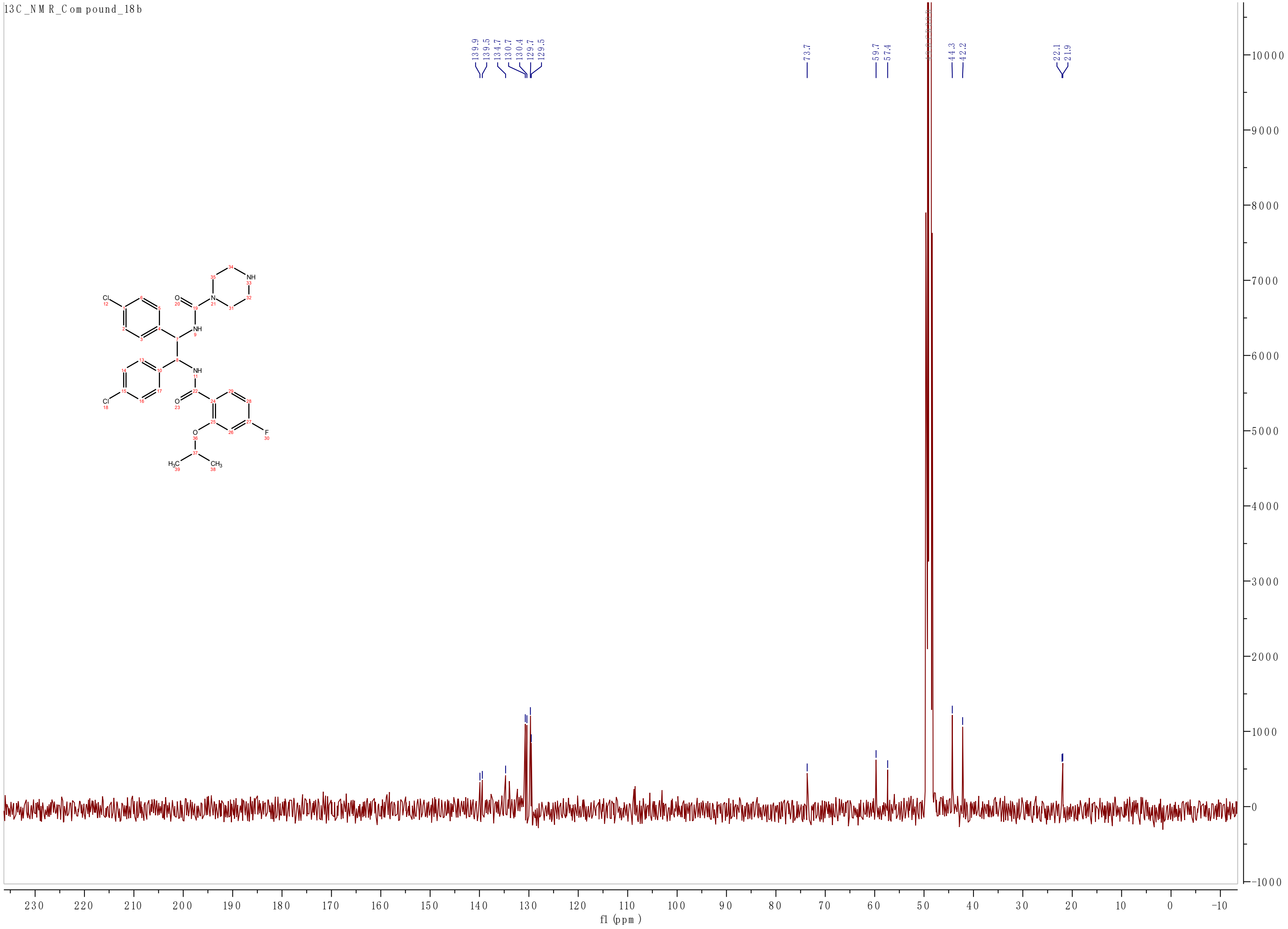

^{1}H NMR Compound **18c**

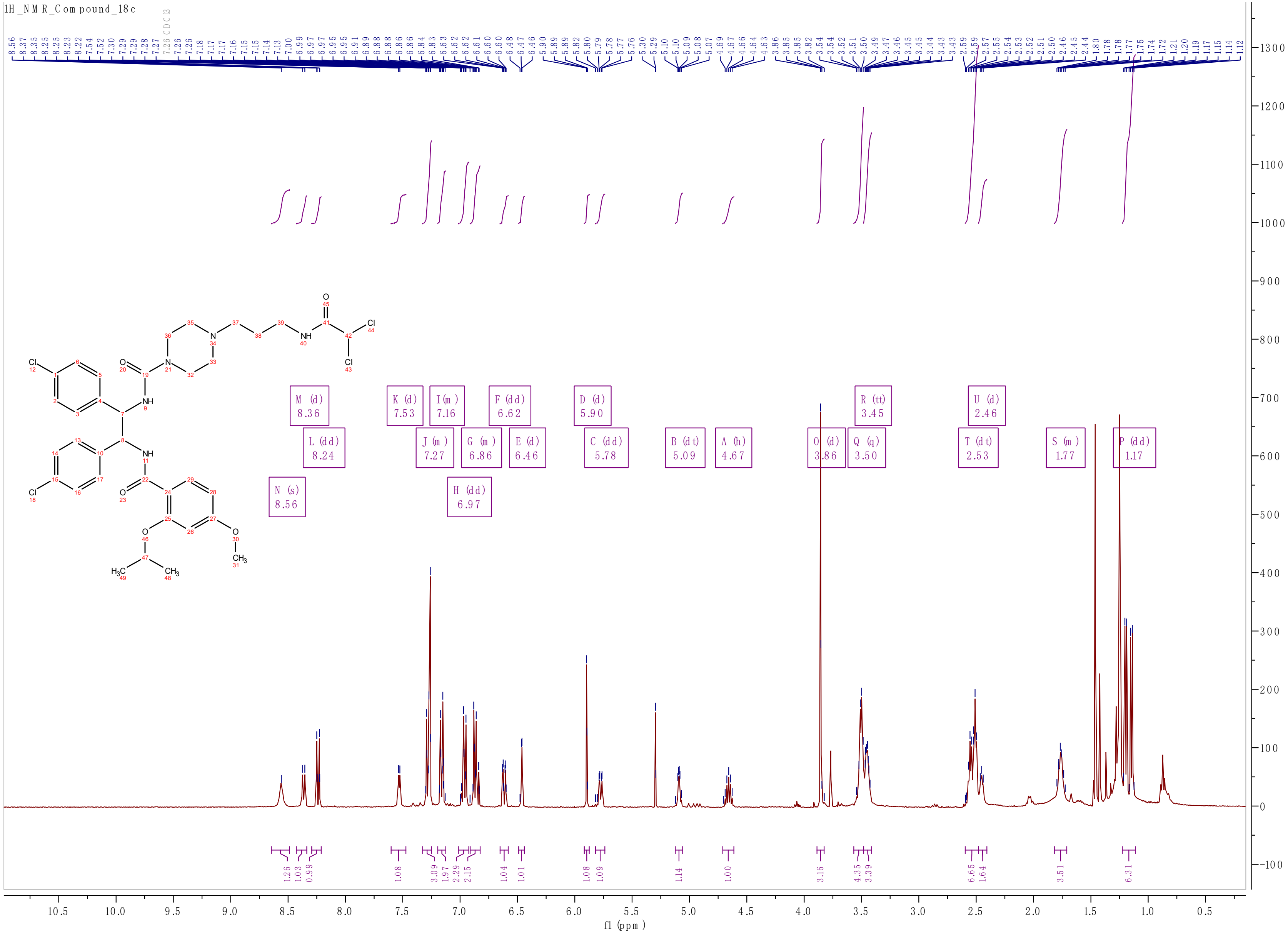

^{13}C NMR Compound **18c**

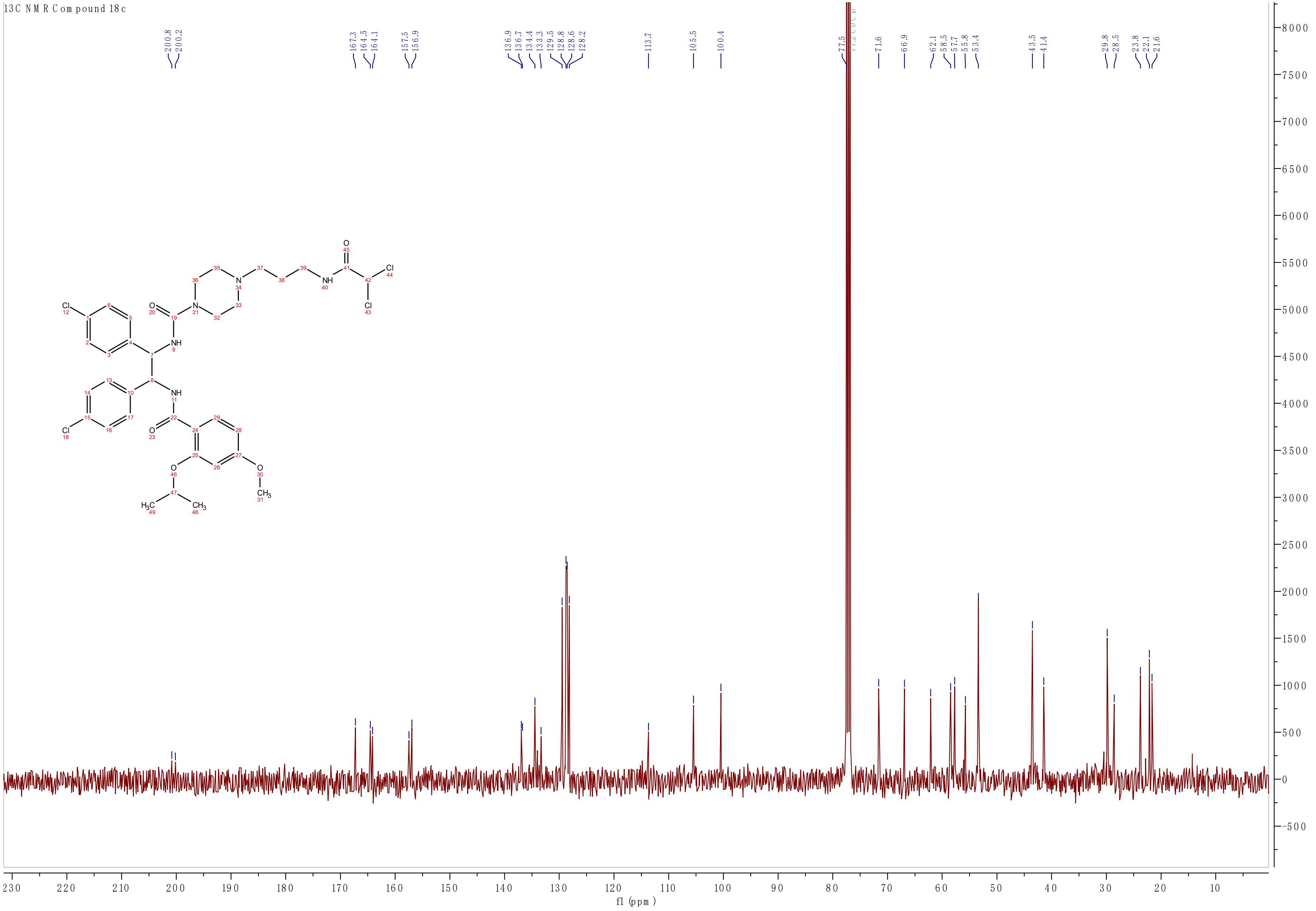

^{1}H NMR Compound **19a**

CD3OD

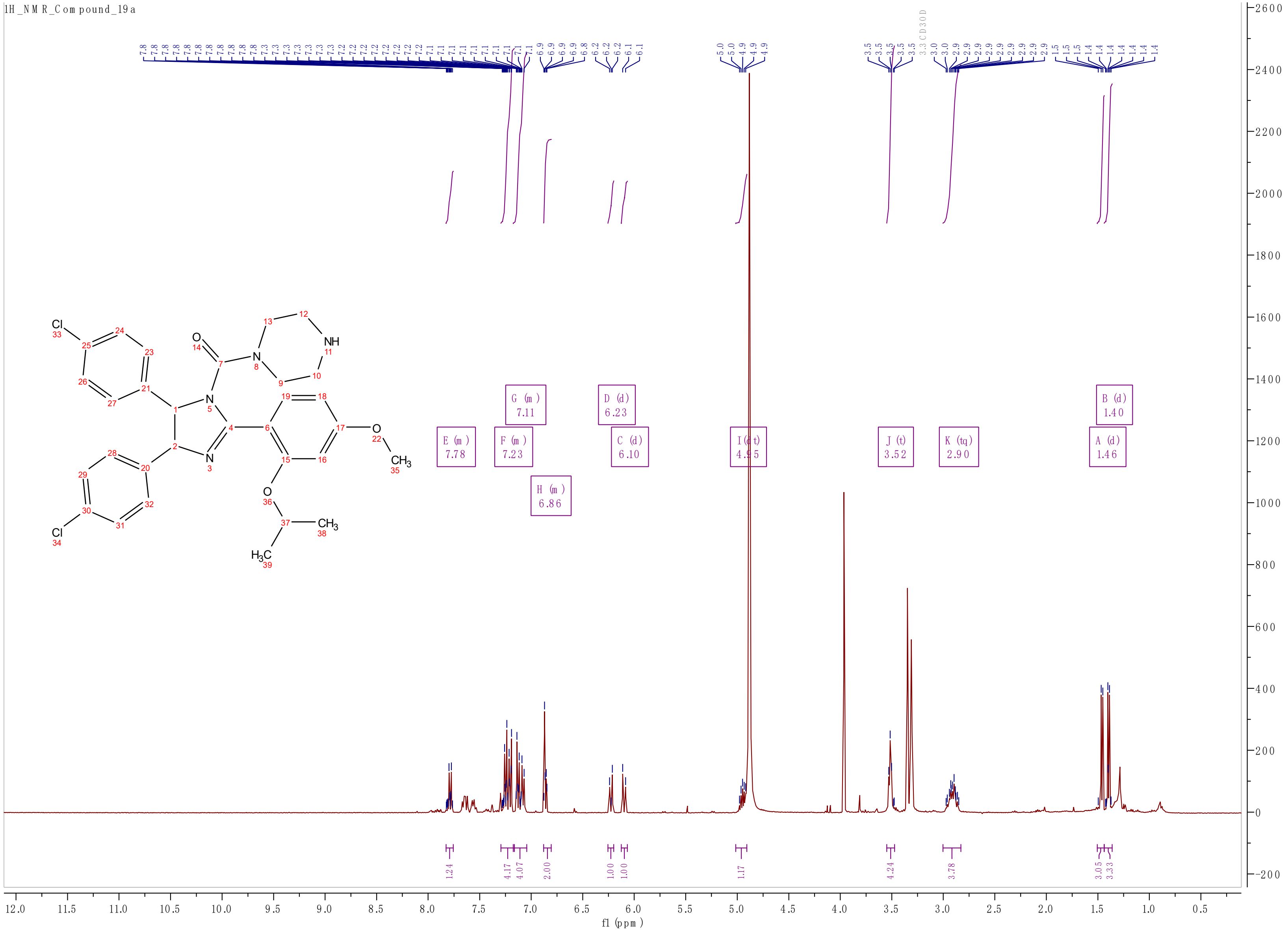

DMSO-d_6

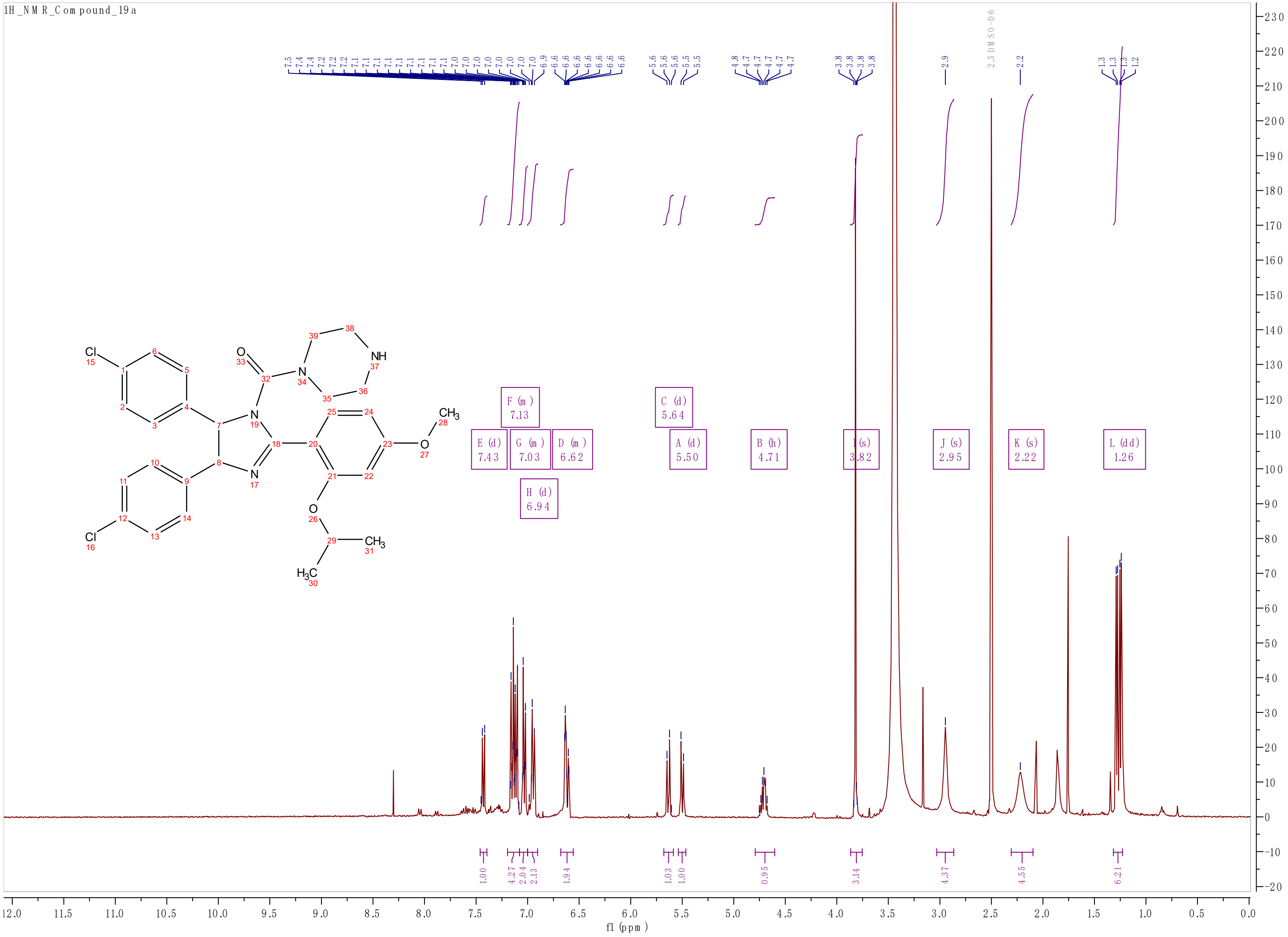

1H_NMR_Compound_19a
E (d) 7.43
F (m) 7.13
G (m) 7.03
H (d) 6.94
D (m) 6.62
C (d) 5.64
A (d) 5.50
B (h) 4.71
I (s) 3.82
J (s) 2.95
K (s) 2.22
L (dd) 1.26
1.00
4.27
2.04
2.13
1.94
1.03
1.00
0.95
3.14
4.37
4.55
6.21
2.50 DMSO-D6
f1 (ppm)

^{13}C NMR Compound **19a**

CD$_3$OD

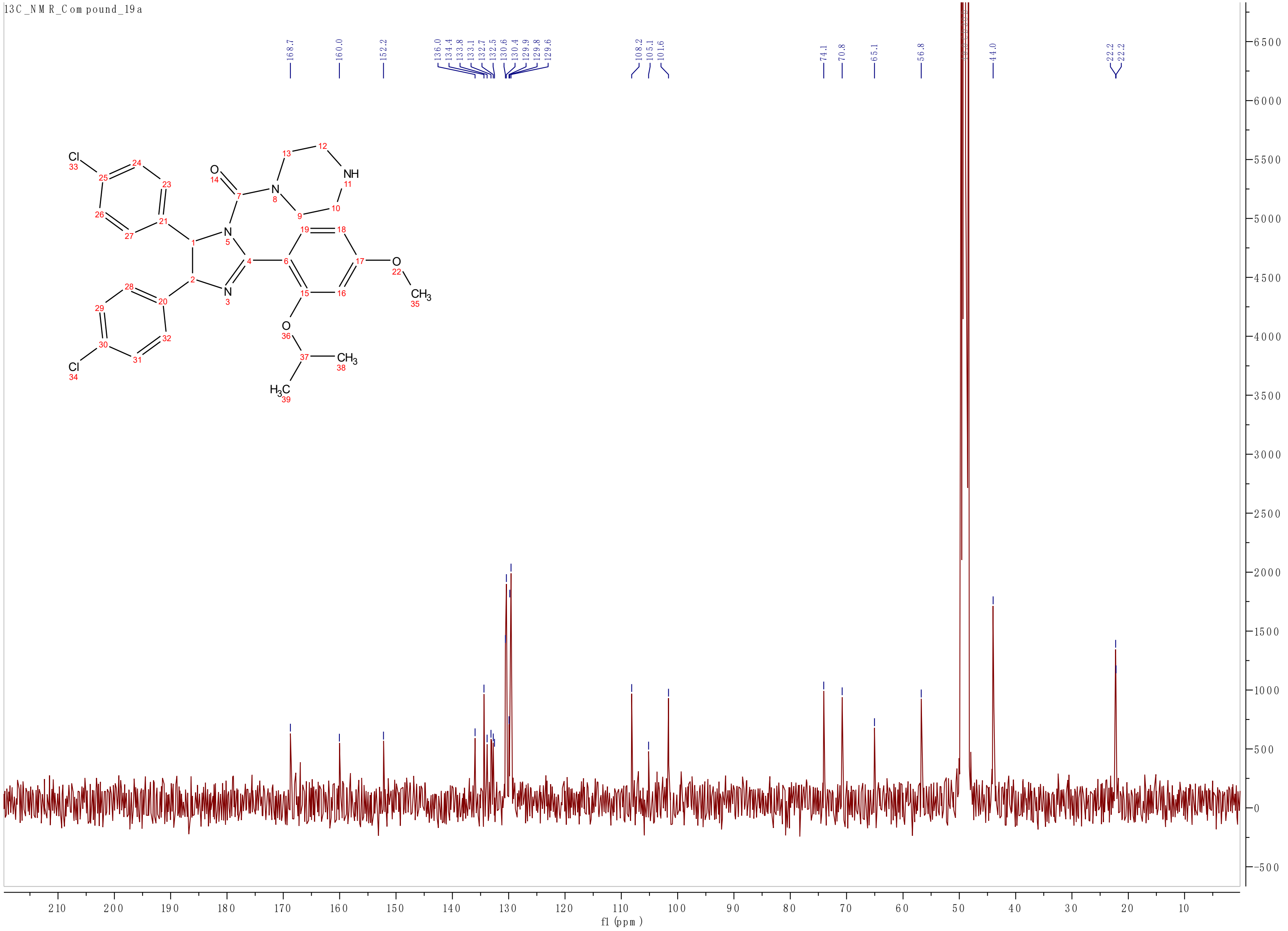

DMSO-d_6

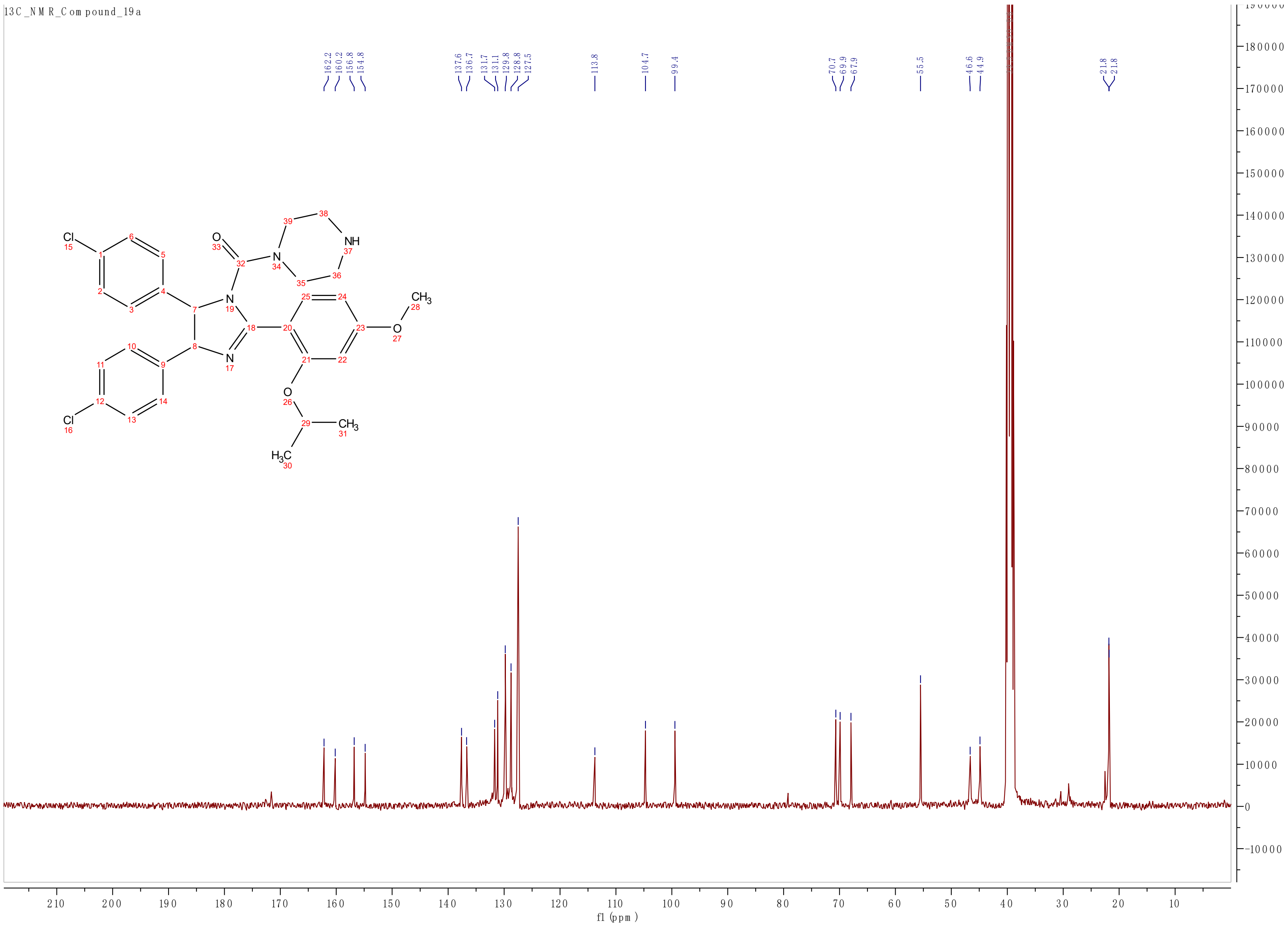

13C_NMR_Compound_19a
162.2
160.2
156.8
154.8
137.6
136.7
131.7
131.1
129.8
128.8
127.5
113.8
104.7
99.4
70.7
69.9
67.9
55.5
46.6
44.9
21.8
21.8
f1 (ppm)

^{1}H NMR Compound **19b**

CD_3OD

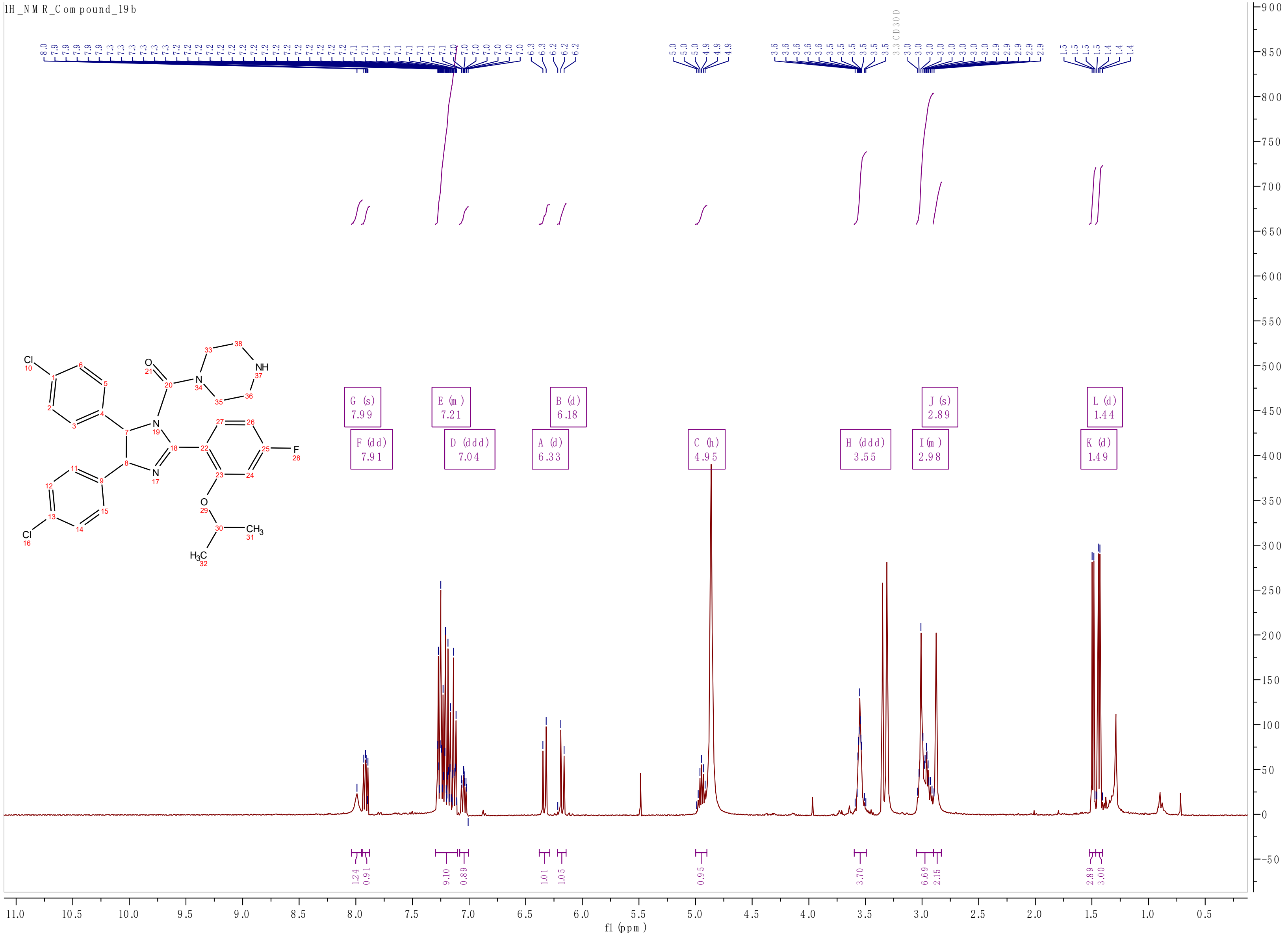

$CDCl_3$

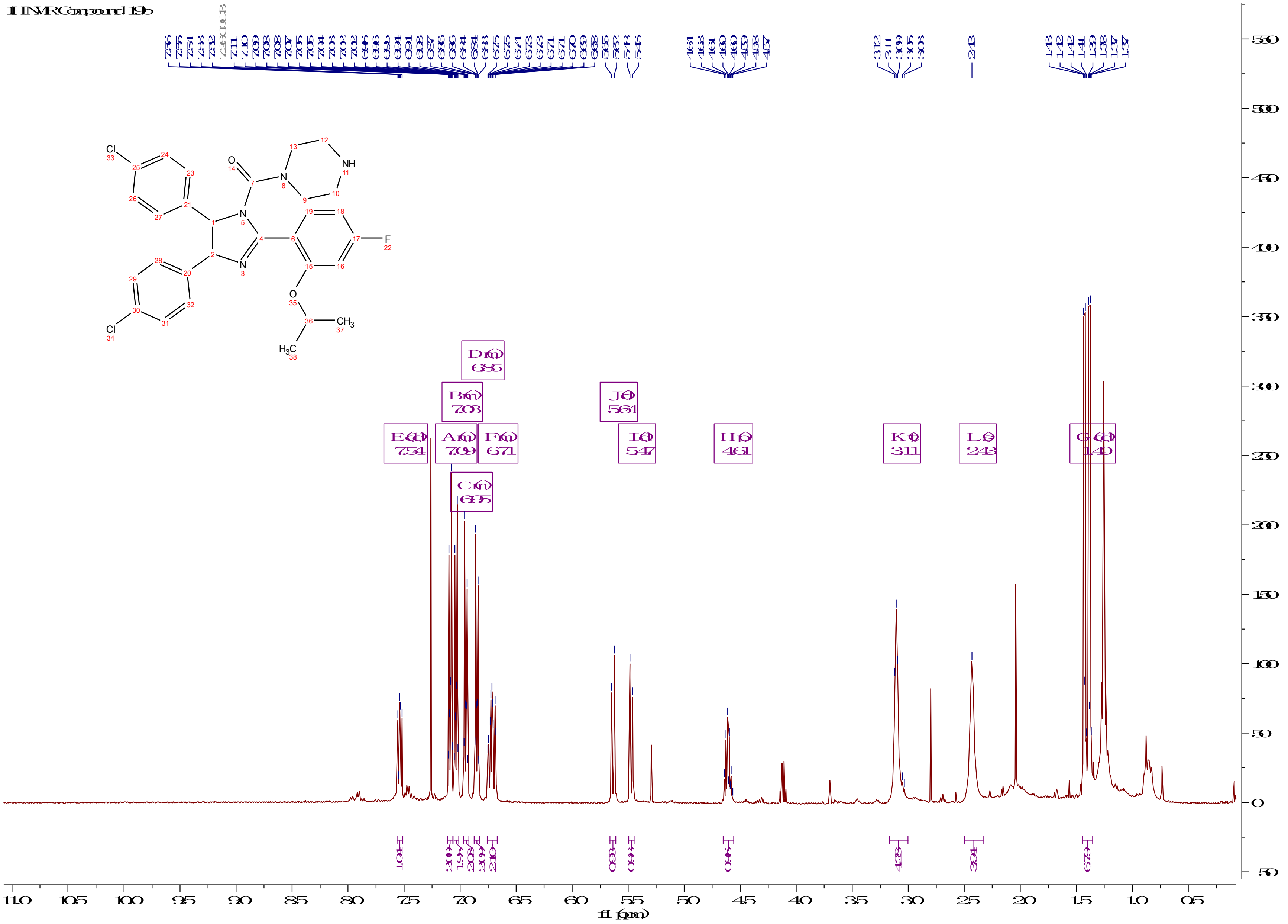

^{13}C NMR Compound **19b**

CD$_3$OD

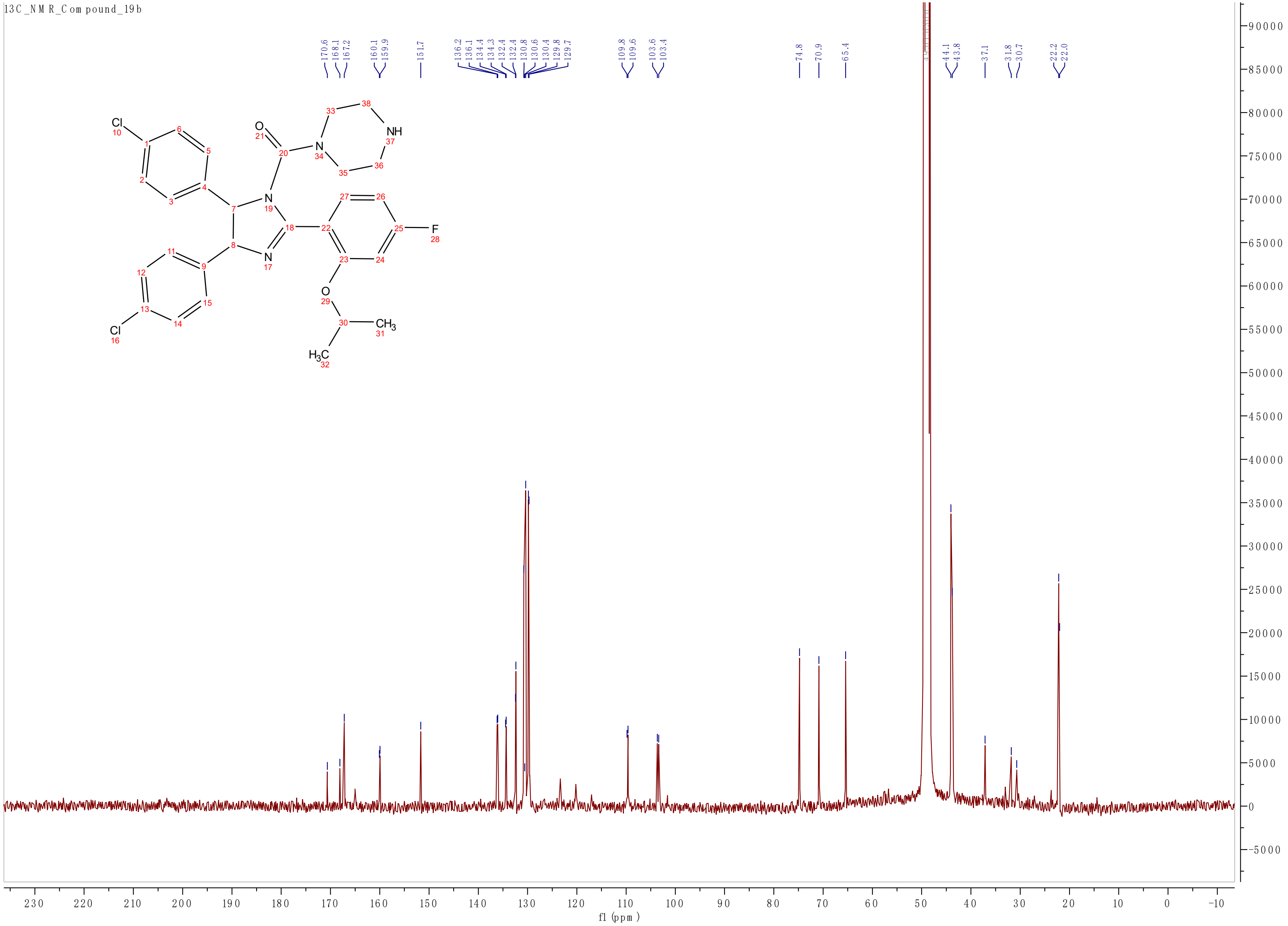

CDCl$_3$

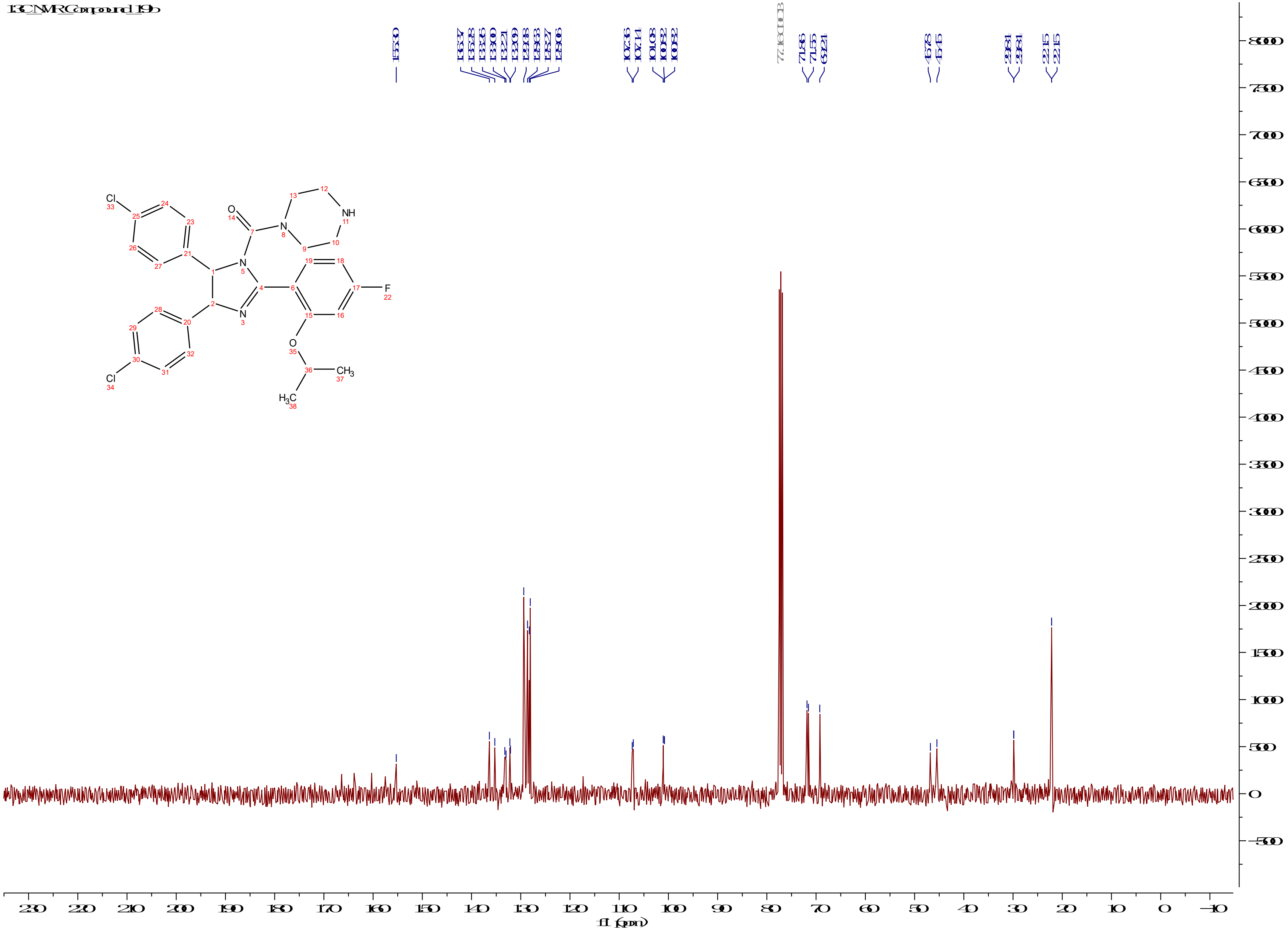

^{19}F NMR Compound **19b**

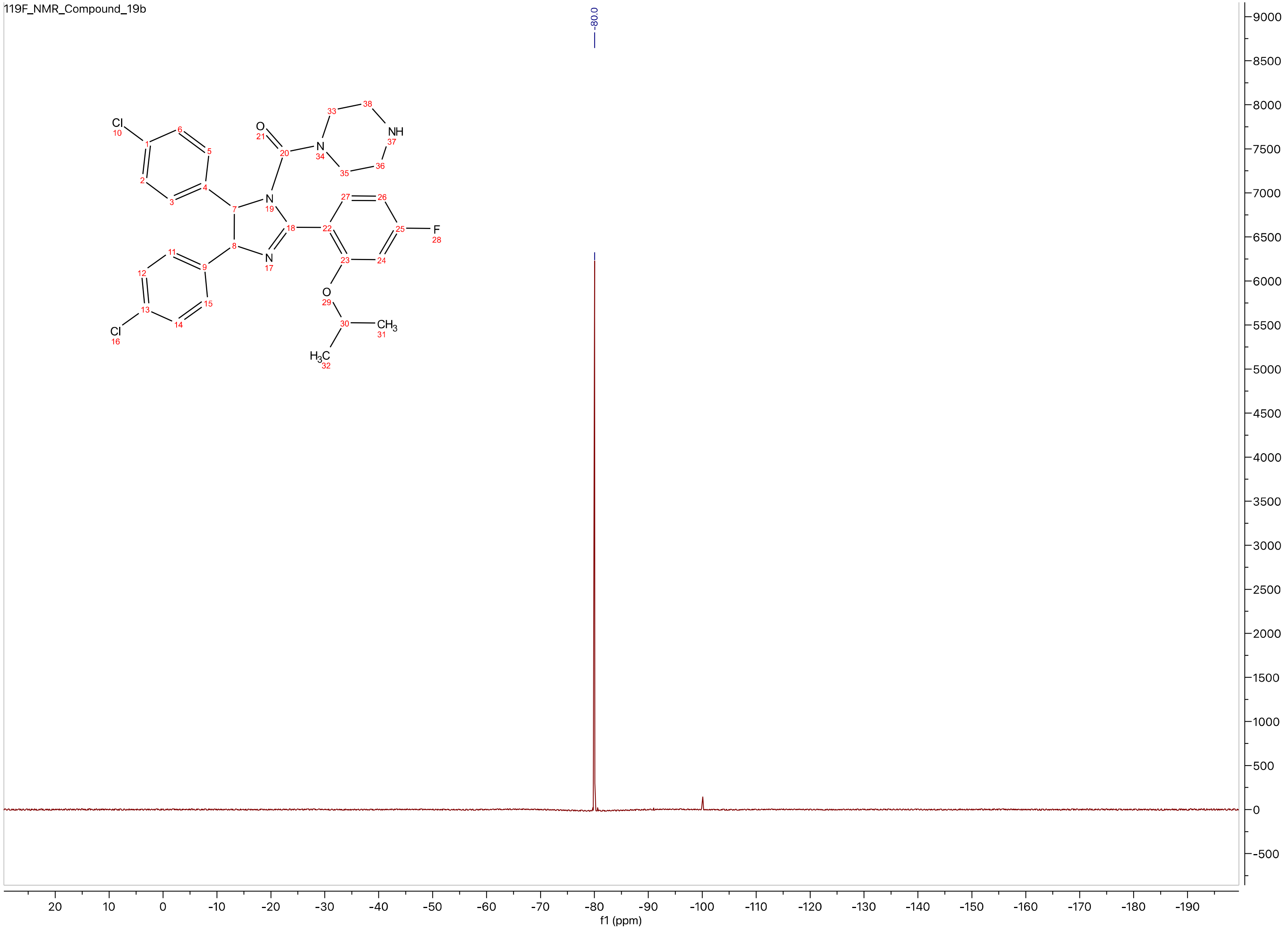

^{1}H NMR Compound 20a

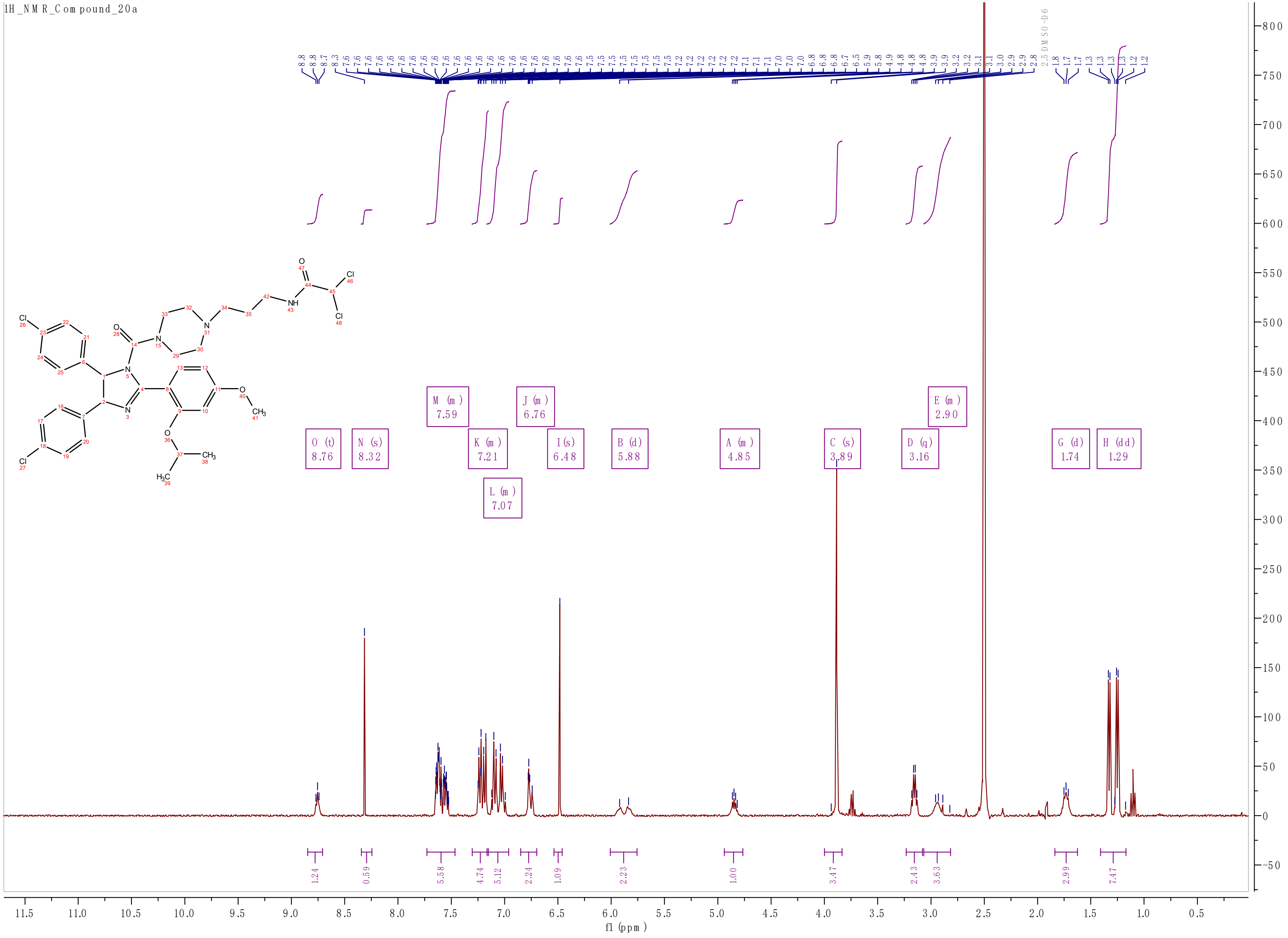

^{13}C NMR Compound 20a

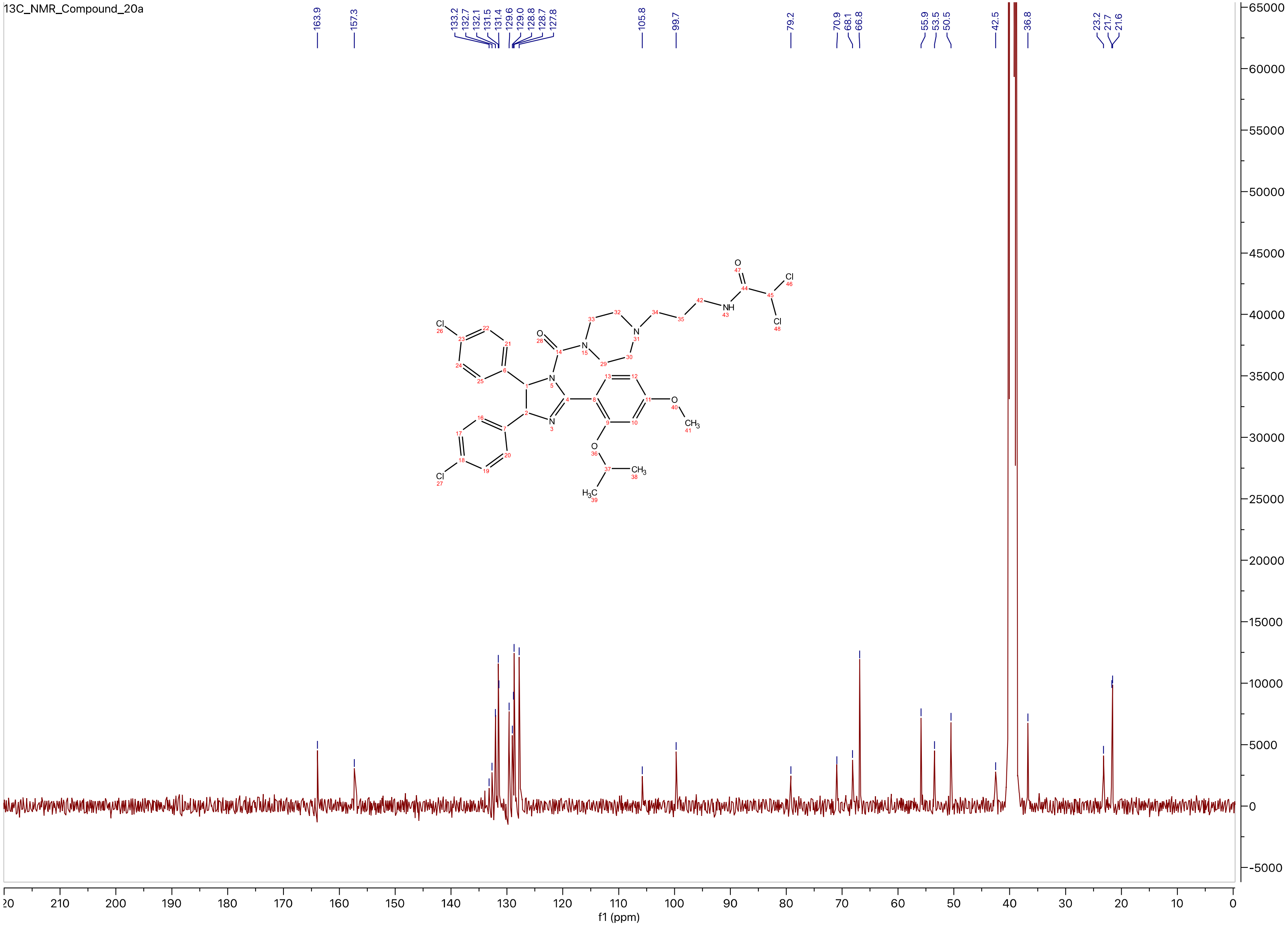

^{1}H NMR Compound **20b**

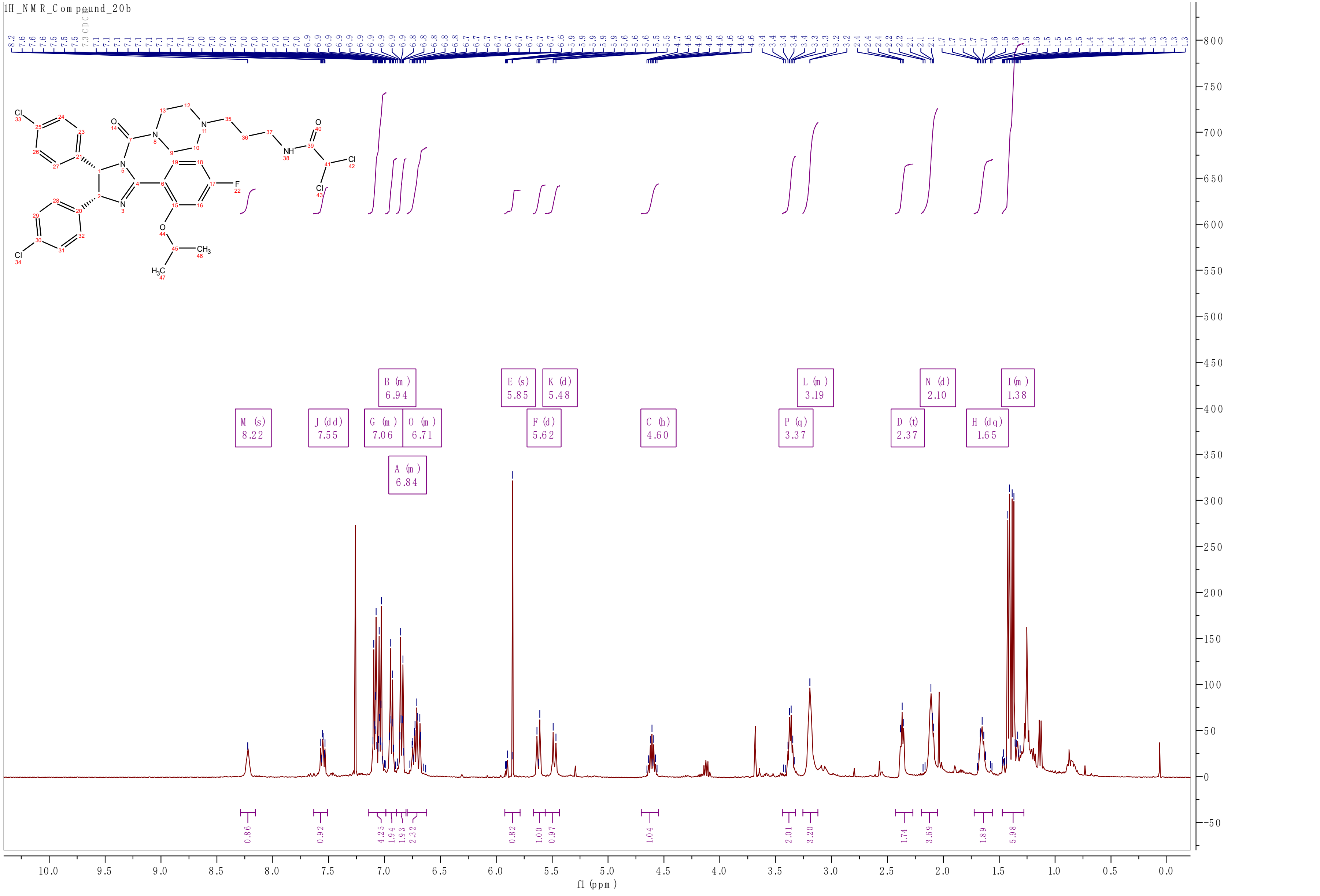

^{13}C NMR Compound **20b**

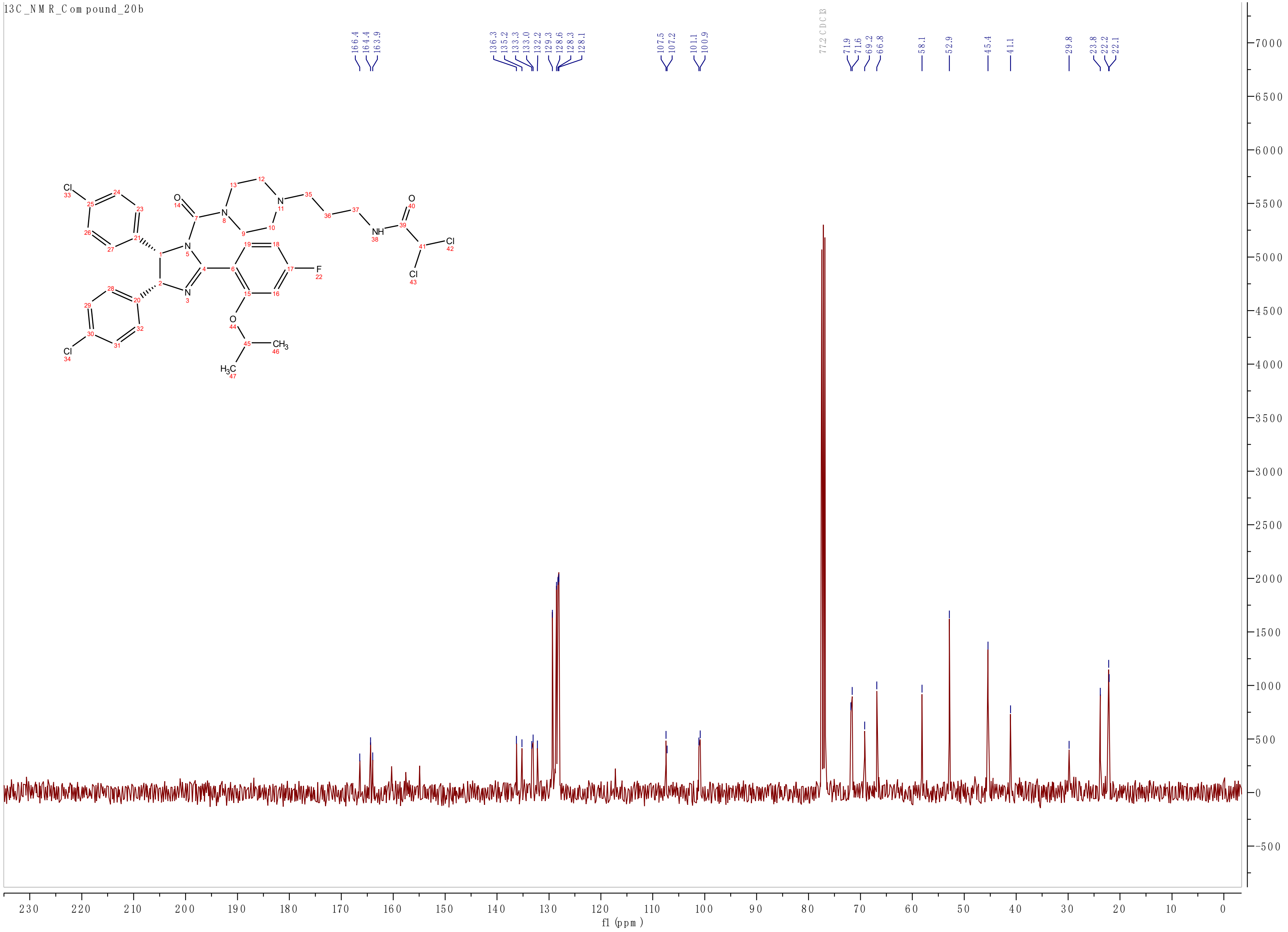

^{1}H NMR Compound **21**

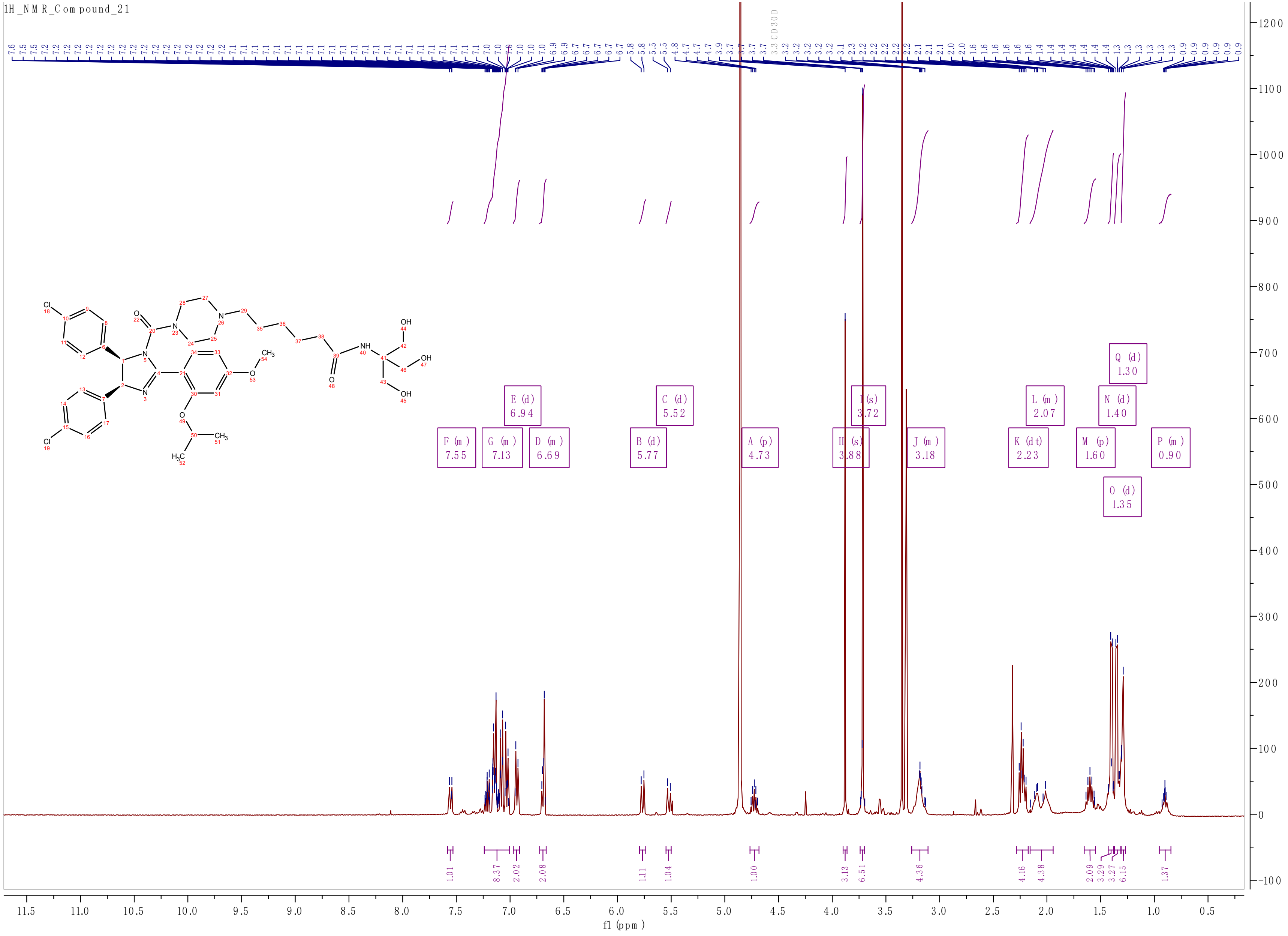

^{13}C NMR Compound **21**

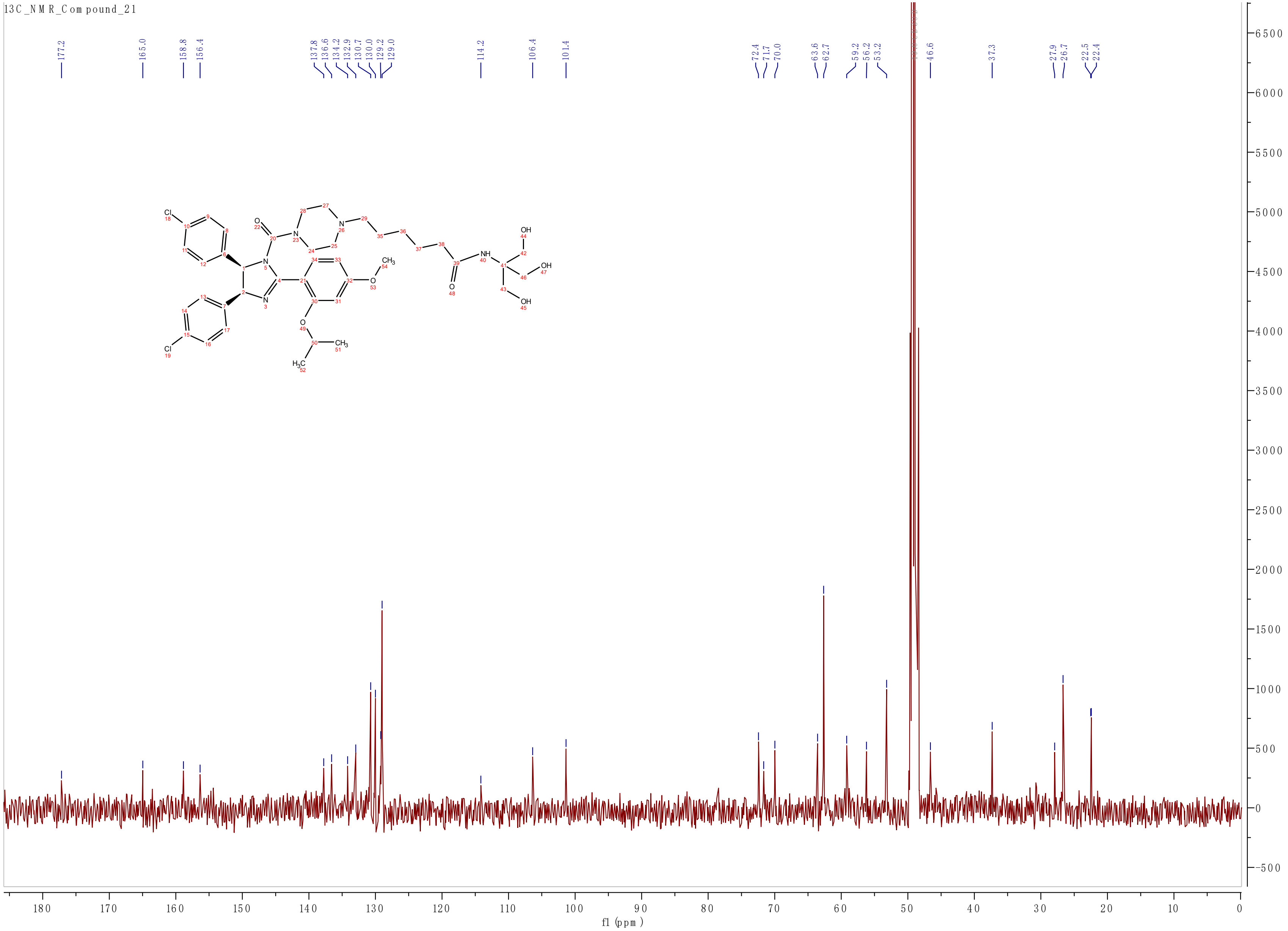

^{1}H NMR Compound 22

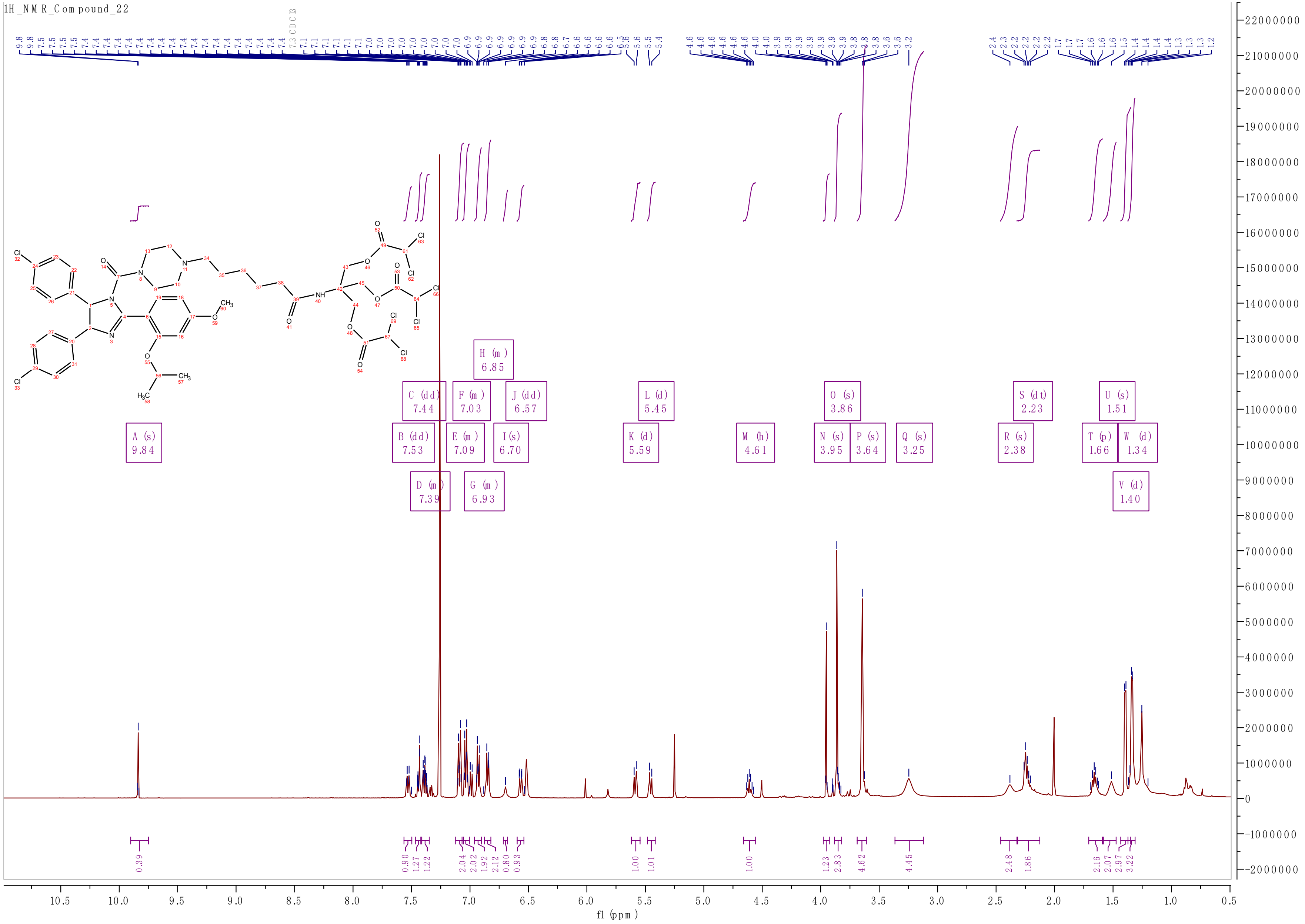

^{13}C NMR Compound 22

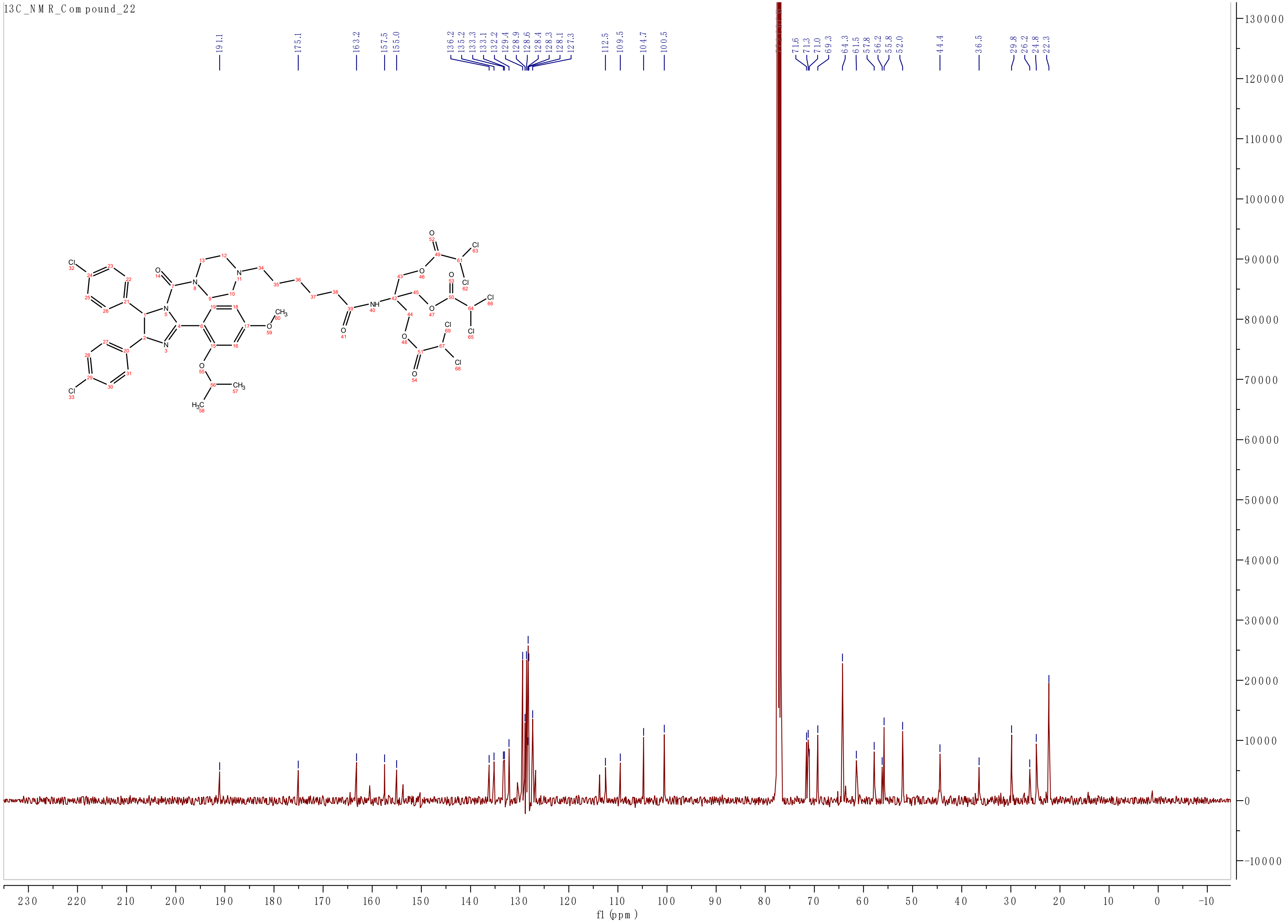

^{1}H NMR Compound 25

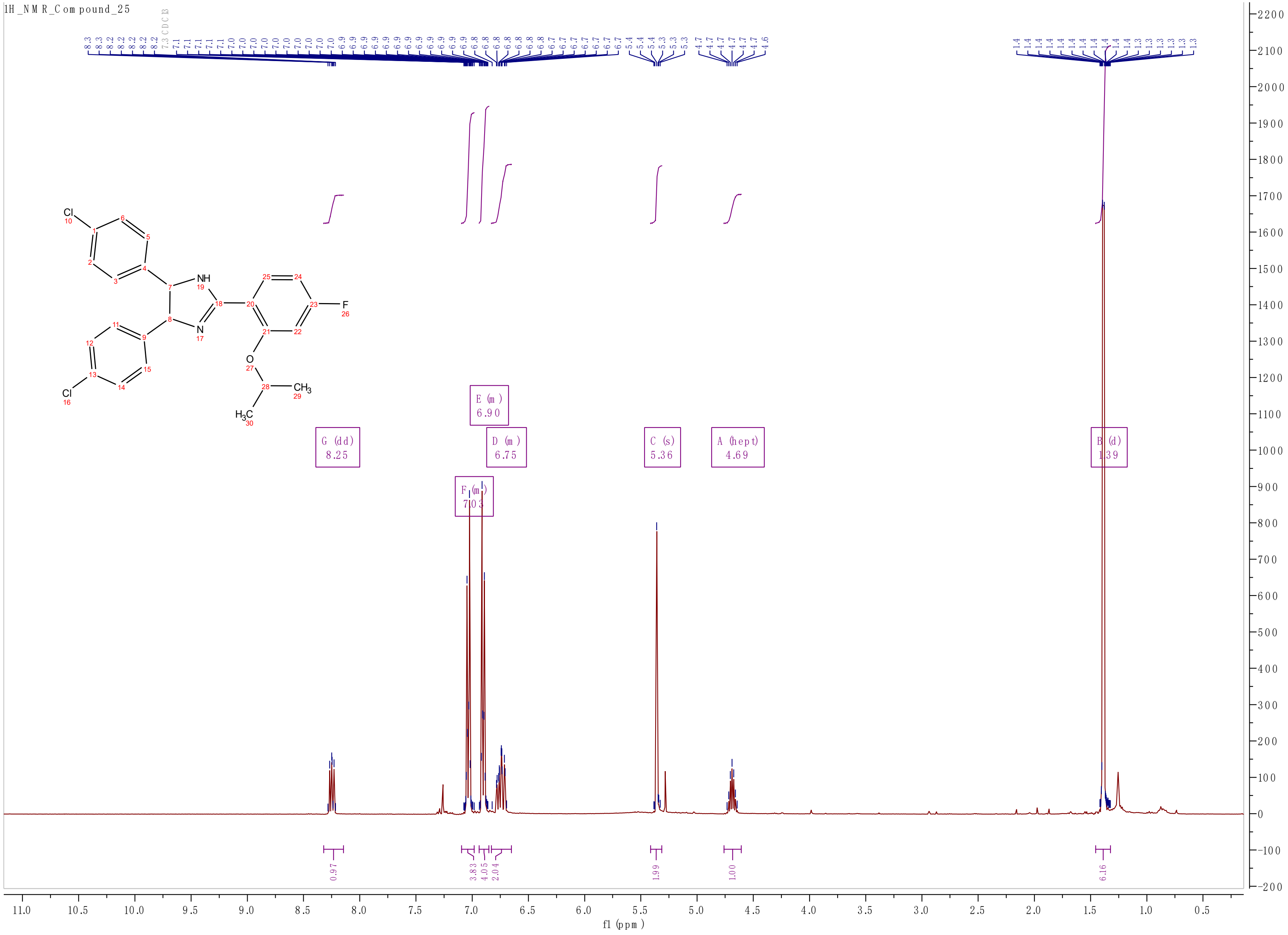

^{13}C NMR Compound 25

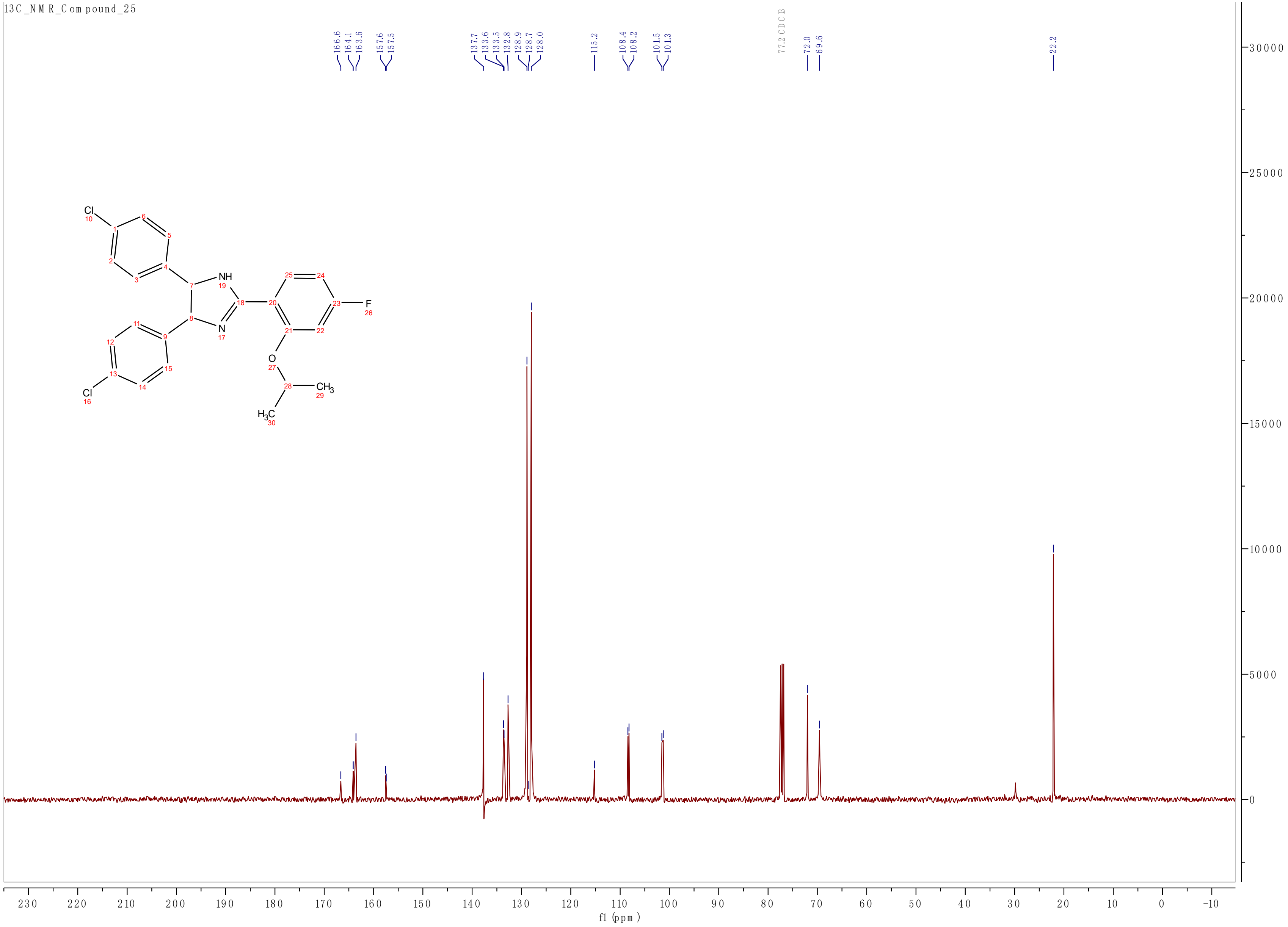

^{19}F NMR Compound **25**

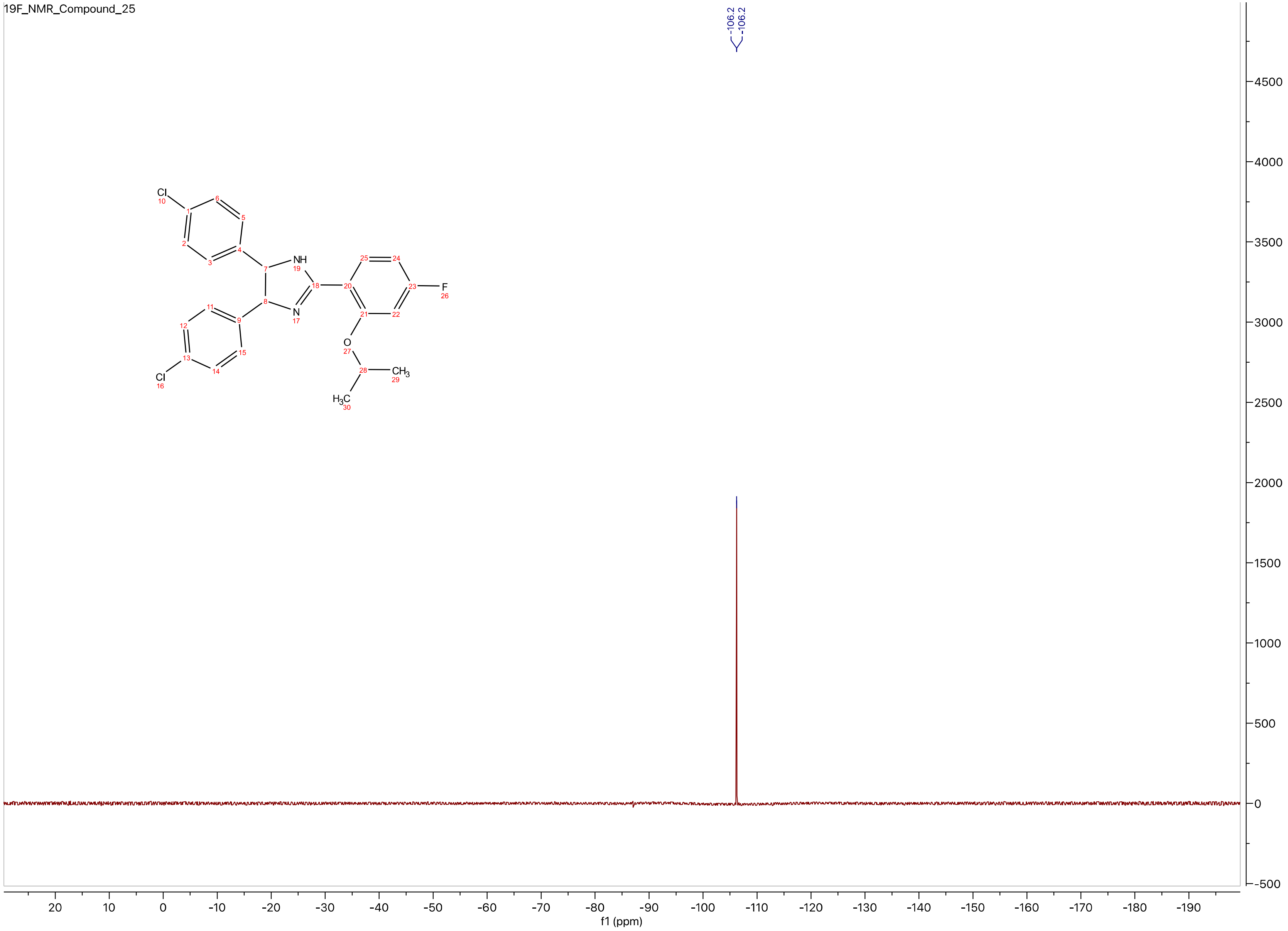

HPLC chromatogram compound 19a

**Result set name:**	nut_ome_rac_ent1		
**Data file:**	BLANK20250904 123914.dx	**Project name:**	LABHPLC
**Sample name:**	BLANK	**Operator:**	SYSTEM (SYSTEM)
**Instrument:**	1260	**Acquired on:**	2025-09-04 12:40:35+02:00
**Inj. volume:**	10.000 µL	**Location:**	1
**Acq. method:**	standard05da10a100.amx		
**Processing method:**	3D UV Quantitative_DefaultMethod.pmx		

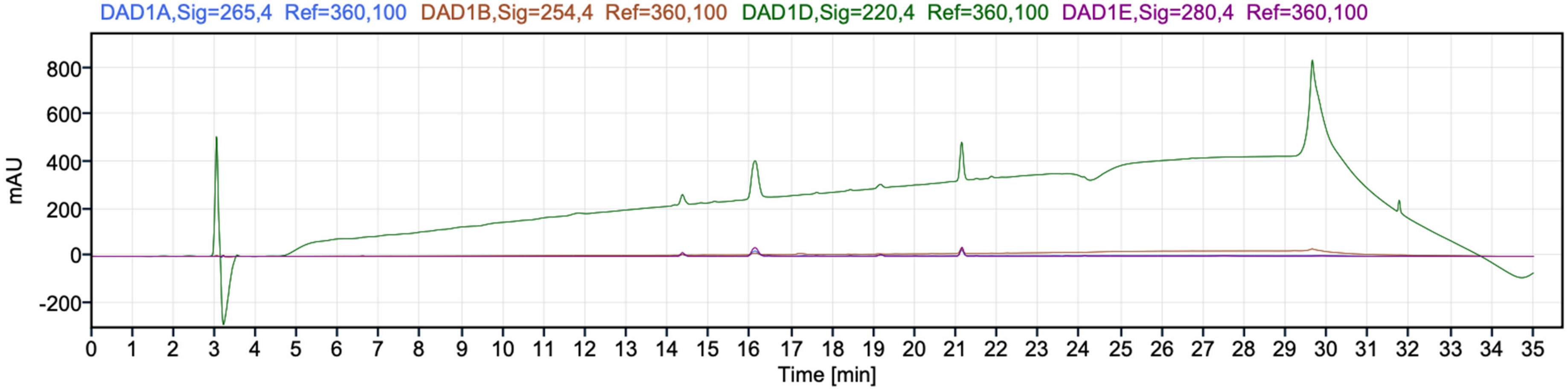

HPLC injection of blank (solution of ACN:H_2O 60:40 + 0,1% TFA)

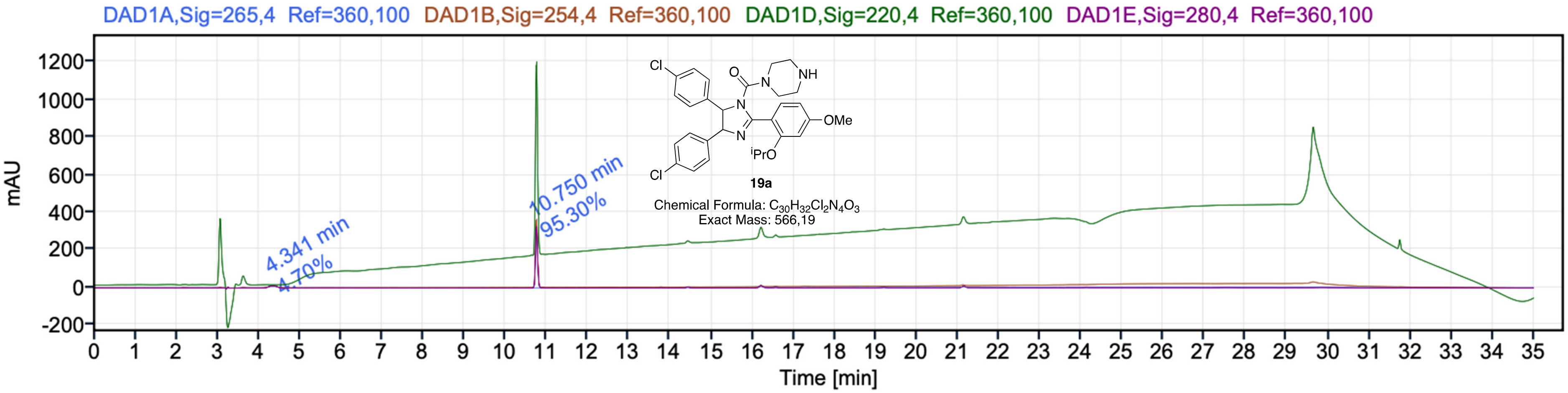

Analytical RP-HPLC column Kinetex® 5µm XB-C18 100Å 150 x 4.6 mm (solvent A: H_2O + 0,1% HCOOH, solvent B: ACN + 0,1% HCOOH, gradient: from 10% to 100% of B in 20 min) t_R= 10.75 (95.30 % purity)

HPLC chromatogram compound *rac*-19a

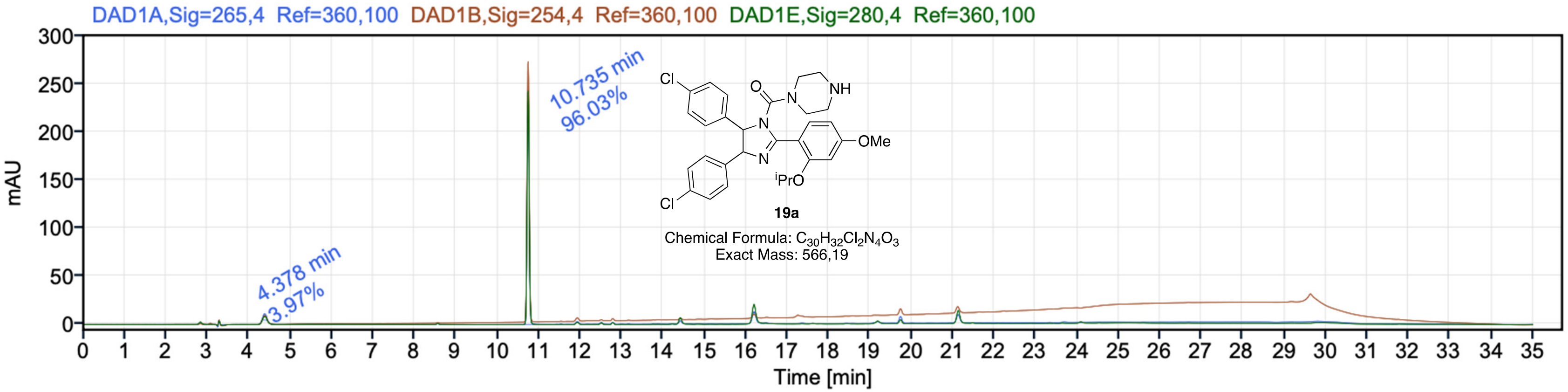

Analytical RP-HPLC column Kinetex® 5µm XB-C18 100Å 150 x 4.6 mm (solvent A: H_2O + 0,1% HCOOH, solvent B: ACN + 0,1% HCOOH, gradient: from 10% to 100% of B in 20 min) t_R= 10.74 min (96.03% purity)

HPLC chromatogram compound 19b

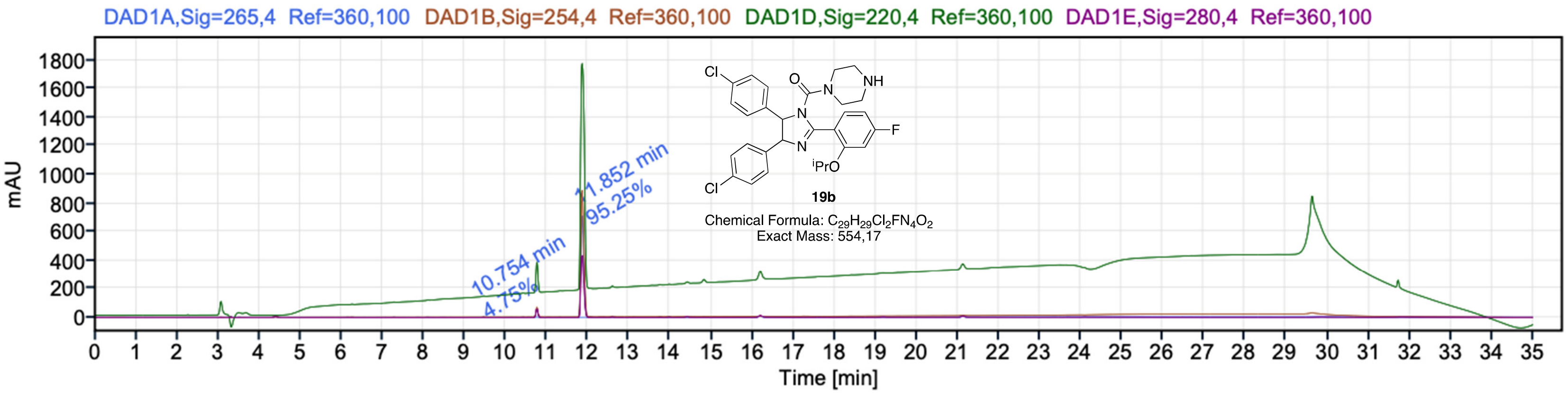

Analytical RP-HPLC column Kinetex® 5µm XB-C18 100Å 150 x 4.6 mm (solvent A: H_2O + 0,1% HCOOH, solvent B: ACN + 0,1% HCOOH, gradient: from 10% to 100% of B in 20 min) t_R=11.852 (95.25% purity)

HPLC chromatogram compound *rac*-19b

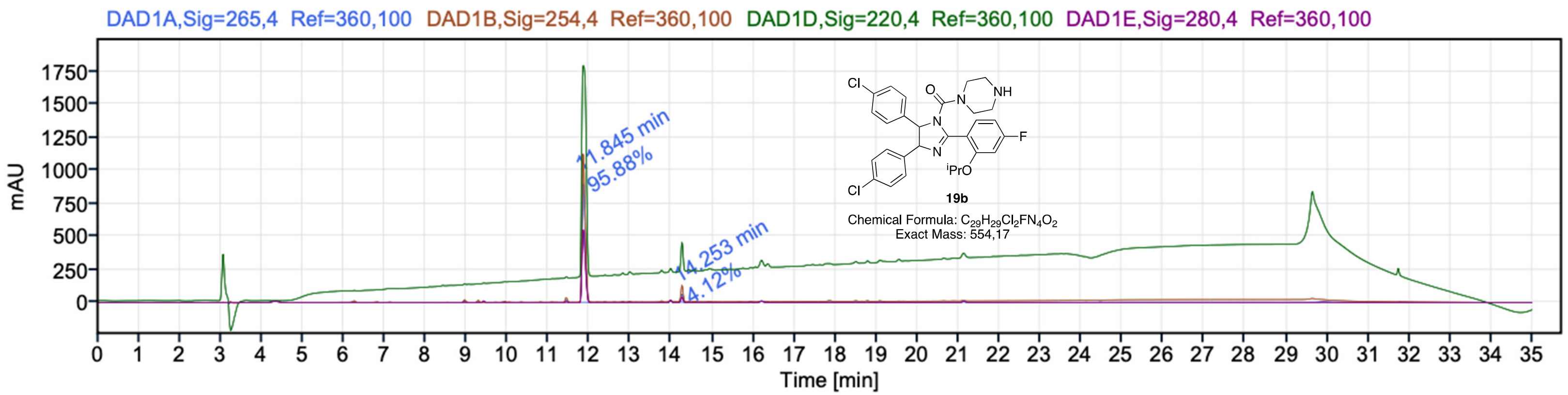

Analytical RP-HPLC (solvent A: H_2O + 0,1% HCOOH, solvent B: ACN + 0,1% HCOOH, gradient: from 10% to 100% of B in 20 min) t_R=11.85 (95.88% purity)

HPLC chromatogram compound *rac*-20a

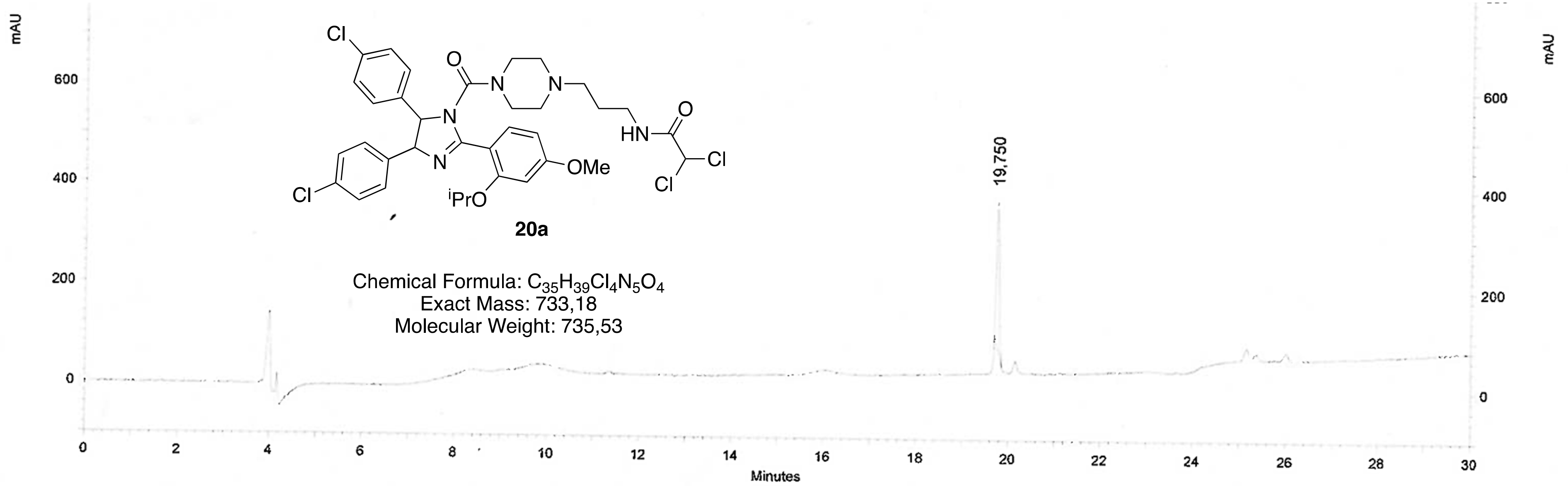

Analytical RP-HPLC (solvent A: H_2O + 0,1% HCOOH, solvent B: ACN + 0,1% HCOOH, gradient: from 10% to 100% of B in 20 min) t_R=19.75 (96.65% purity)

HPLC chromatogram compound *rac*-20b

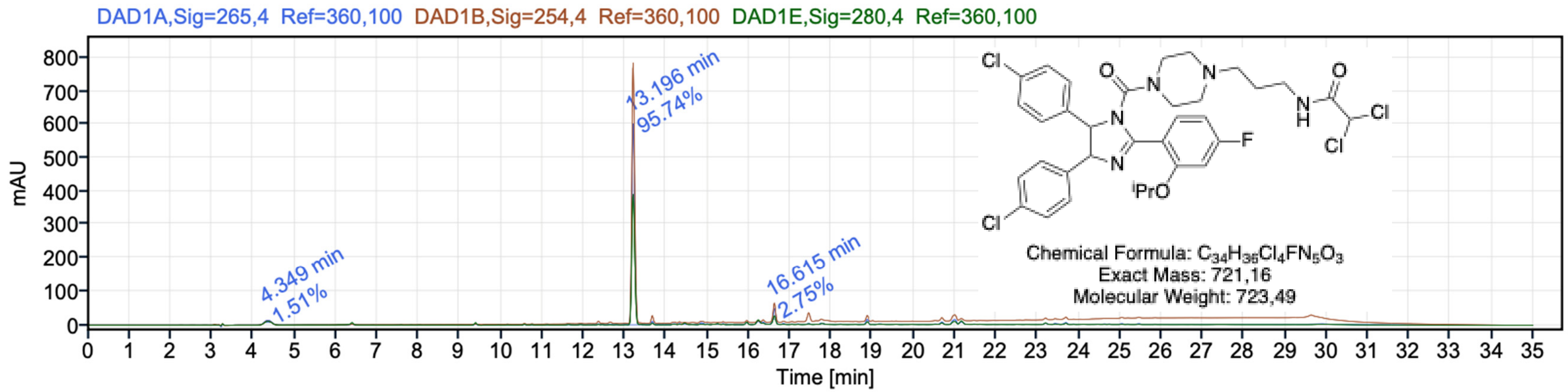

Analytical RP-HPLC (solvent A: H_2O + 0,1% HCOOH, solvent B: ACN + 0,1% HCOOH, gradient: from 10% to 100% of B in 20 min) t_R=13.196 (95.74% purity)

The mass of synthesized compounds was assessed by injecting 1 µL of each sample into a Vanquish Flex Ultra High-Performance Liquid Chromatography (UHPLC) system coupled to a High-Resolution Orbitrap Exploris 240 mass spectrometer (ThermoFisher Scientific). The separation was performed on a Waters BEH C_{18} column (100×2.1 mm L×I.D., 1.7 µm) operated under reversed phase conditions by using H_2O and acetonitrile + 0.1% formic acid as mobile phase. All the samples were analyzed in positive mode.

Sample	Found m/z	Theoretical m/z	Δ (ppm)
**18c**	752.1934	752.1935 $[M+H]^+$	0.13
**19a**	567.1921	567.1924 $[M+H]^+$	0.52
**19b**	555.1721	555.1724 $[M+H]^+$	0.54
**22**	1114.1221	1114.1217 $[M+H]^+$	0.36
**20a**	734.1825	734.1829 $[M+H]^+$	0.54
**20b**	724.1591	724.1599 $[M+H]^+$	1.10
**28**	1661.0508	1661.05096 $[M]^+$	0.4

Exact mass Compound **18c**

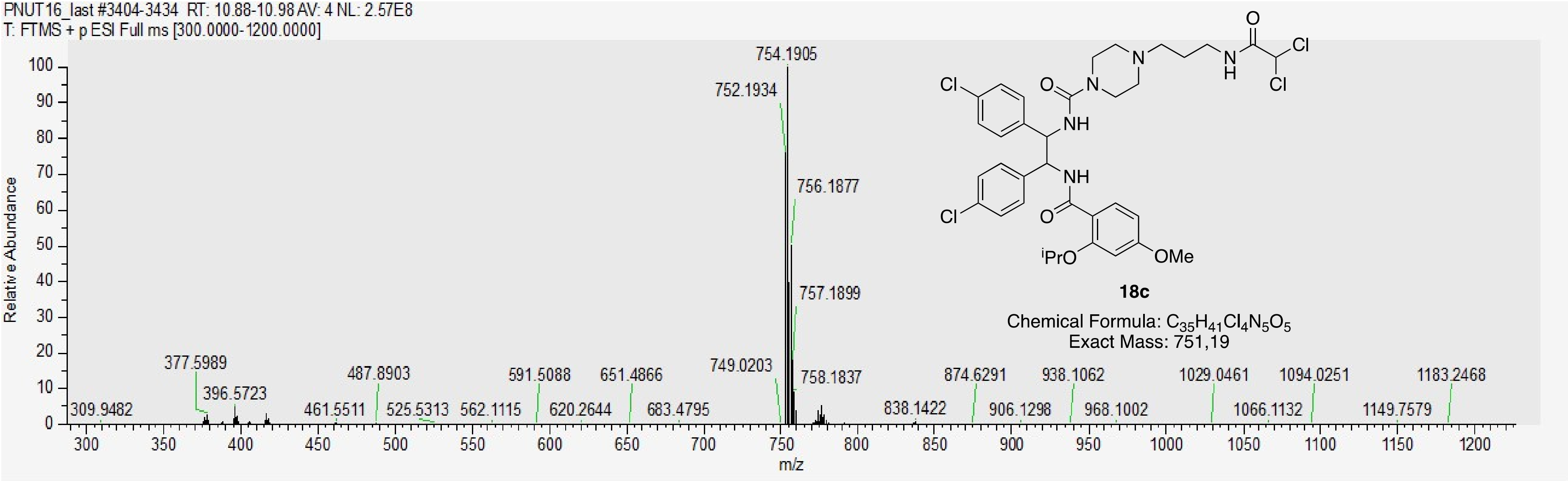

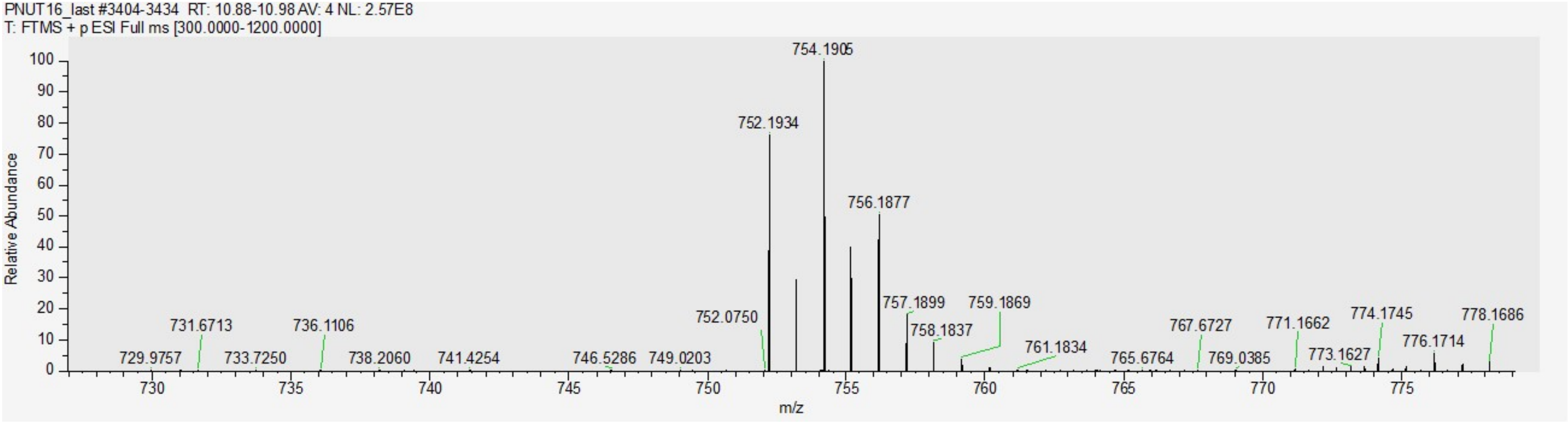

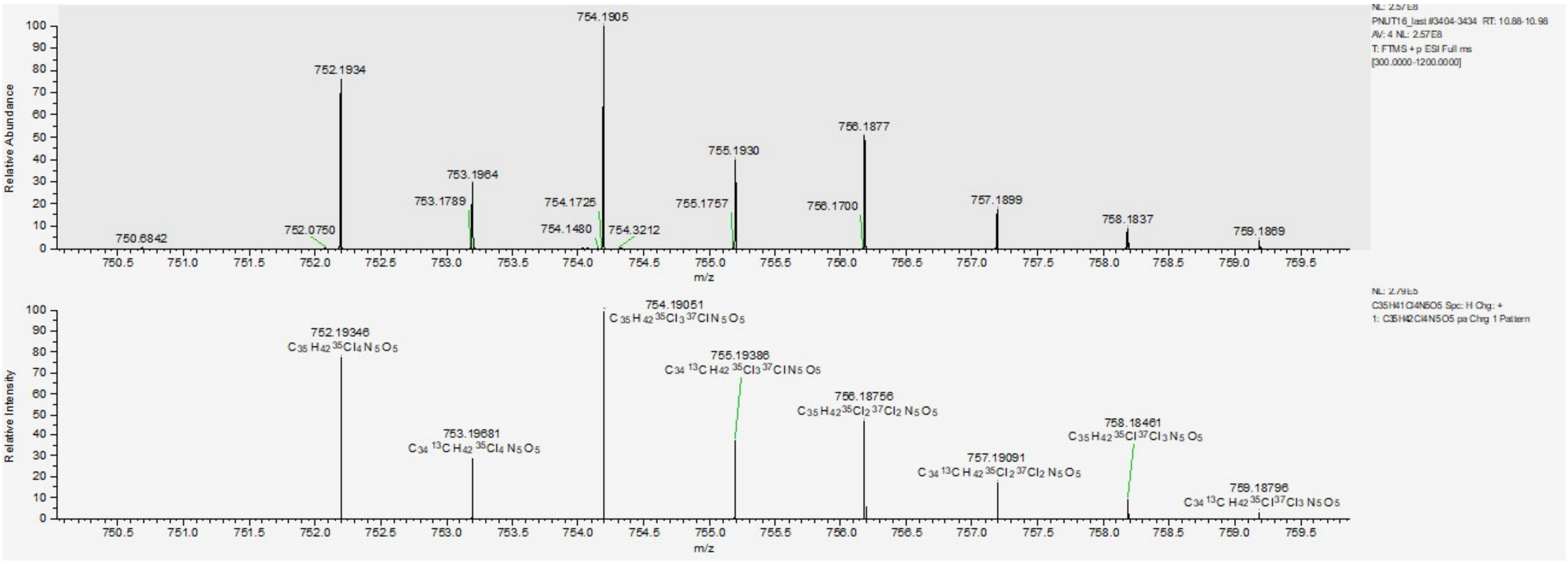

NL: 2.57E8
PNUT16_last #3404-3434 RT: 10.88-10.98
AV: 4 NL: 2.57E8
T: FTMS + p ESI Full ms [300.0000-1200.0000]
754.1905
752.1934
753.1964
753.1789
754.1725
754.1480
754.3212
755.1930
755.1757
756.1877
756.1700
757.1899
758.1837
759.1869
750.6842
752.0750
Relative Abundance
m/z
NL: 2.79E5
C35H41Cl4N5O5 Spc: H Chg: +
1: C35H42Cl4N5O5 pa Chrg 1 Pattern
754.19051
$C_{35}H_{42}{}^{35}Cl_3{}^{37}ClN_5O_5$
752.19346
$C_{35}H_{42}{}^{35}Cl_4N_5O_5$
755.19386
$C_{34}{}^{13}CH_{42}{}^{35}Cl_3{}^{37}ClN_5O_5$
756.18756
$C_{35}H_{42}{}^{35}Cl_2{}^{37}Cl_2N_5O_5$
753.19681
$C_{34}{}^{13}CH_{42}{}^{35}Cl_4N_5O_5$
758.18461
$C_{35}H_{42}{}^{35}Cl{}^{37}Cl_3N_5O_5$
757.19091
$C_{34}{}^{13}CH_{42}{}^{35}Cl_2{}^{37}Cl_2N_5O_5$
759.18796
$C_{34}{}^{13}CH_{42}{}^{35}Cl{}^{37}Cl_3N_5O_5$
Relative Intensity
m/z

Exact mass Compound 19a

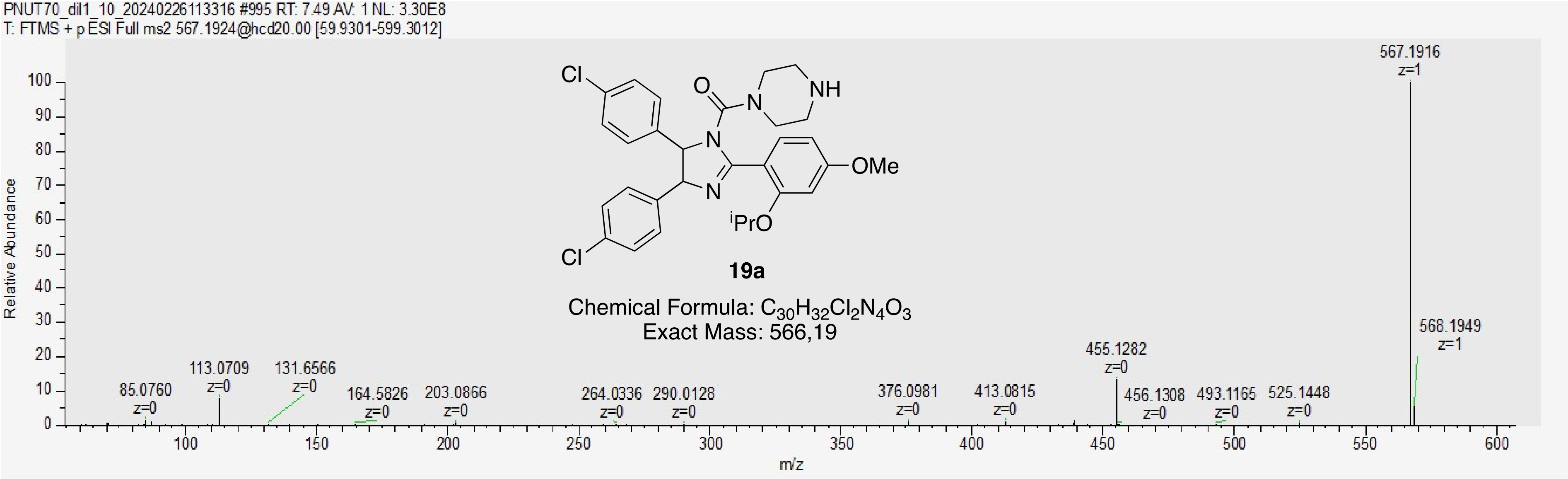

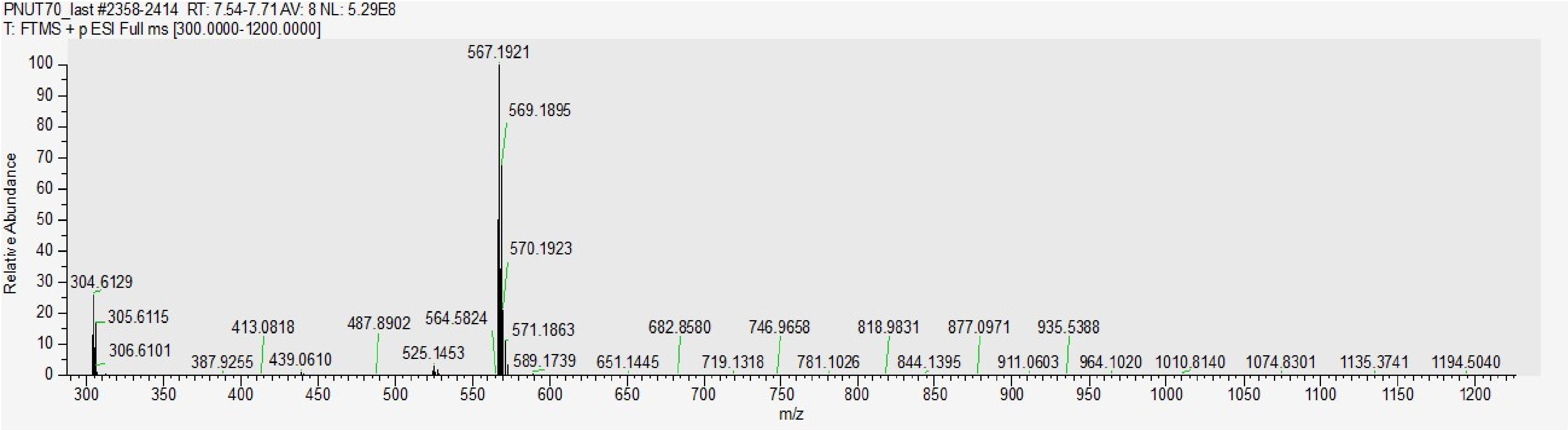

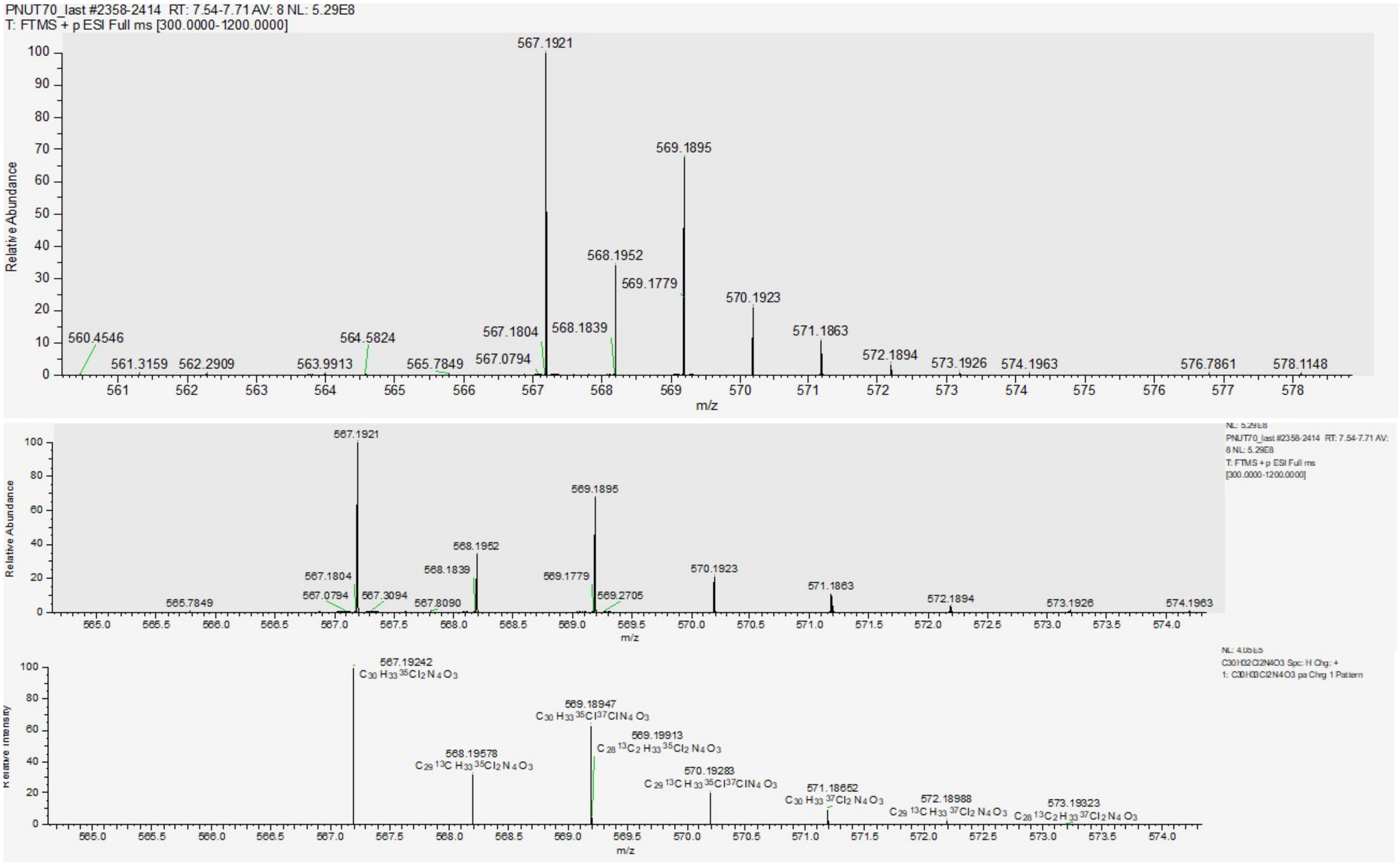
PNUT70_last #2358-2414 RT: 7.54-7.71 AV: 8 NL: 5.29E8
T: FTMS + p ESI Full ms [300.0000-1200.0000]
Relative Abundance
m/z
567.1921
569.1895
568.1952
569.1779
570.1923
571.1863
572.1894
573.1926
574.1963
560.4546
561.3159
562.2909
563.9913
564.5824
565.7849
567.0794
567.1804
568.1839
576.7861
578.1148
567.3094
567.8090
569.2705
NL: 4.05E5
C30H32Cl2N4O3 Spc: H Chg: +
1: C30H33Cl2N4O3 pa Chrg 1 Pattern
Relative Intensity
567.19242
C30 H33 35Cl2 N4 O3
569.18947
C30 H33 35Cl 37Cl N4 O3
569.19913
C28 13C2 H33 35Cl2 N4 O3
568.19578
C29 13C H33 35Cl2 N4 O3
570.19283
C29 13C H33 35Cl 37Cl N4 O3
571.18652
C30 H33 37Cl2 N4 O3
572.18988
C29 13C H33 37Cl2 N4 O3
573.19323
C28 13C2 H33 37Cl2 N4 O3

Exact mass Compound 19b

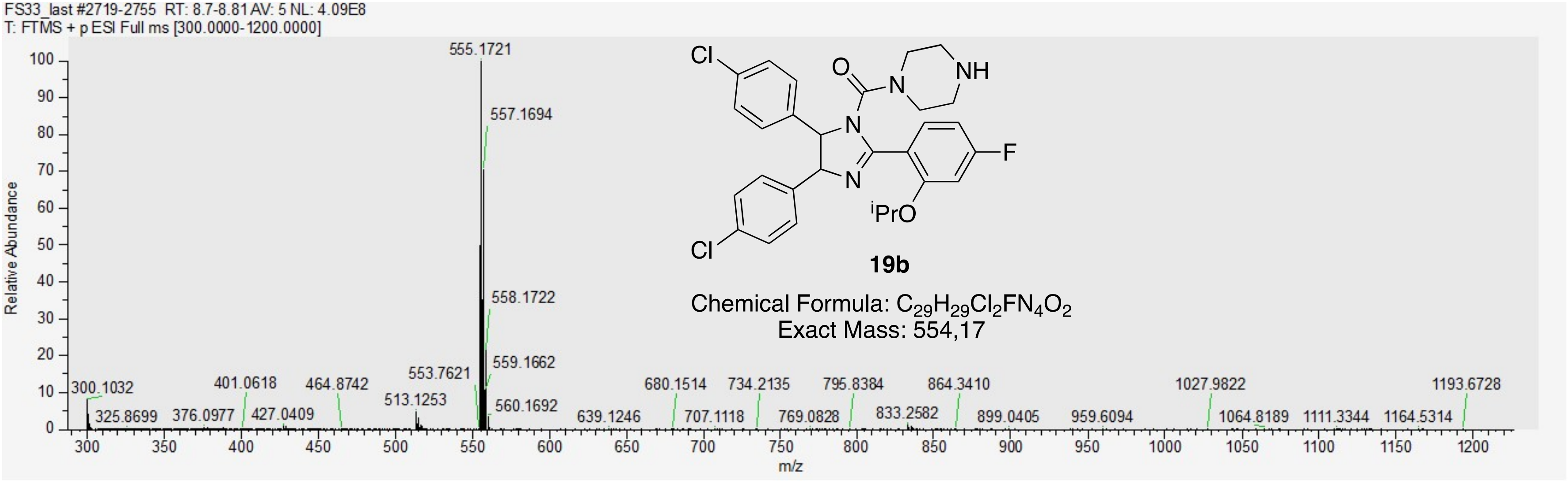

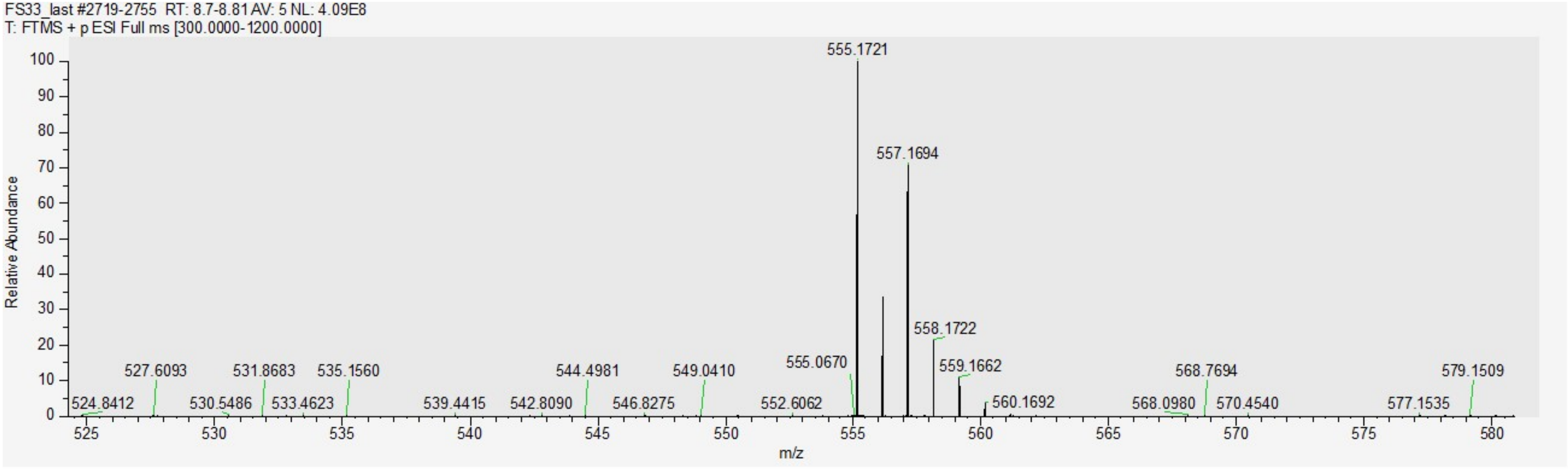

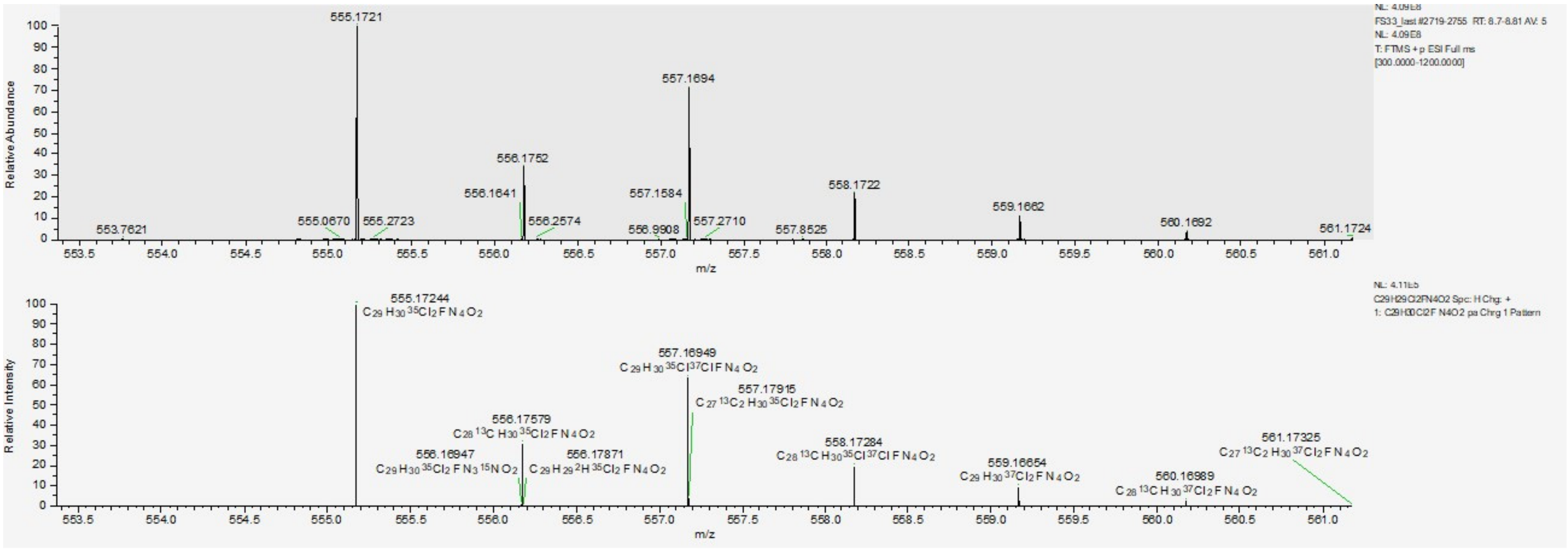

NL: 4.09E8
FS33_last #2719-2755 RT: 8.7-8.81 AV: 5
NL: 4.09E8
T: FTMS + p ESI Full ms
[300.0000-1200.0000]
555.1721
557.1694
556.1752
556.1641
557.1584
558.1722
559.1662
553.7621
555.0670
555.2723
556.2574
556.9908
557.2710
557.8525
560.1692
561.1724
Relative Abundance
m/z
NL: 4.11E5
C29H29Cl2FN4O2 Spc: H Chg: +
1: C29H30Cl2FN4O2 pa Chrg 1 Pattern
555.17244
C29 H30 35Cl2 F N4 O2
557.16949
C29 H30 35Cl 37Cl F N4 O2
557.17915
C27 13C2 H30 35Cl2 F N4 O2
556.17579
C28 13C H30 35Cl2 F N4 O2
556.16947
C29 H30 35Cl2 F N3 15N O2
556.17871
C29 H29 2H 35Cl2 F N4 O2
558.17284
C28 13C H30 35Cl 37Cl F N4 O2
559.16654
C29 H30 37Cl2 F N4 O2
560.16989
C28 13C H30 37Cl2 F N4 O2
561.17325
C27 13C2 H30 37Cl2 F N4 O2
Relative Intensity
m/z

Exact mass Compound **20a**

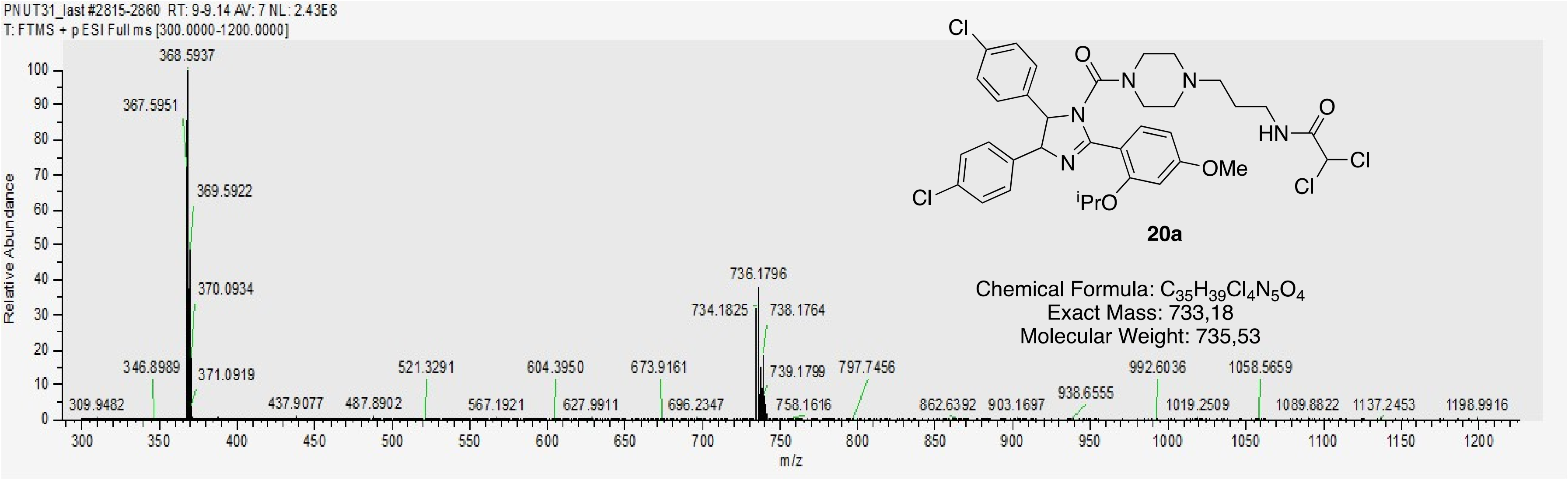

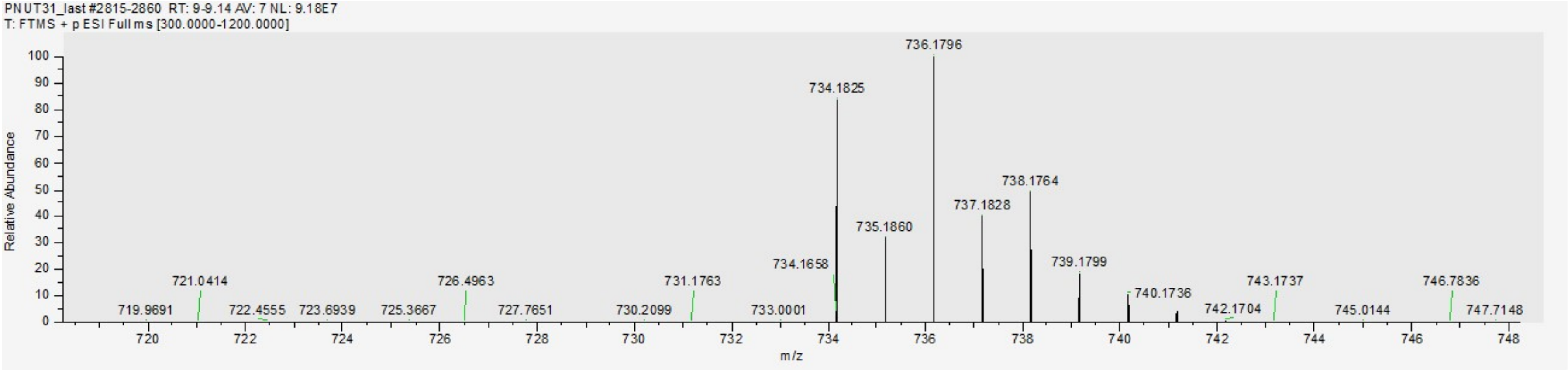

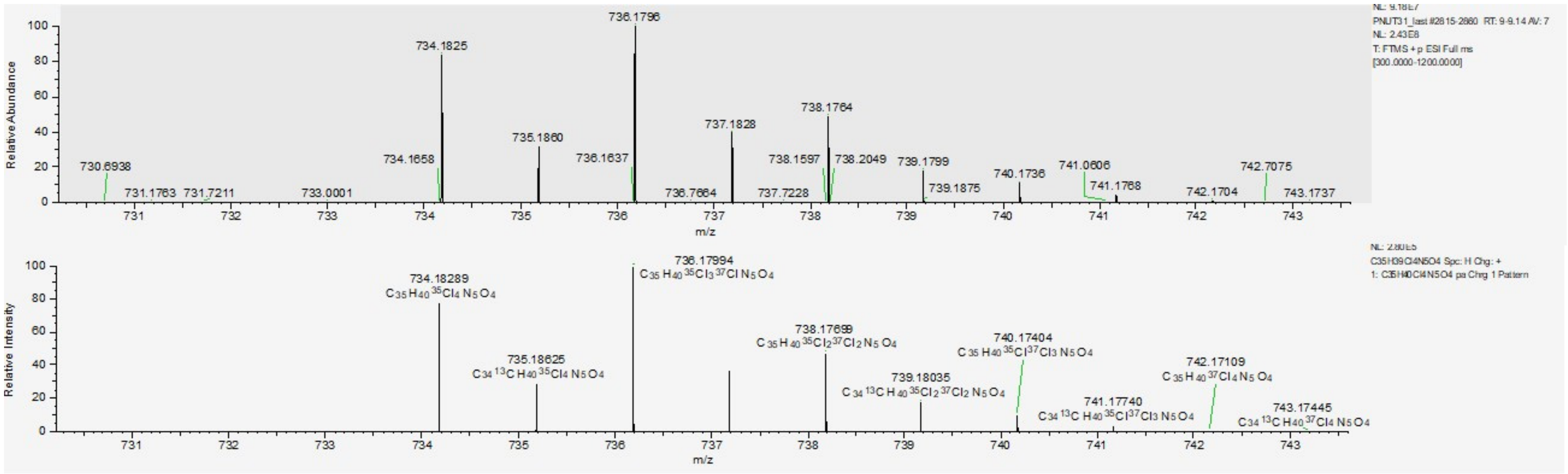

NL: 2.43E8
T: FTMS + p ESI Full ms [300.0000-1200.0000]
Relative Abundance
736.1796
734.1825
735.1860
737.1828
738.1764
739.1799
740.1736
741.0606
742.7075
m/z
NL: 2.80E5
C35H39Cl4N5O4 Spc: H Chg: +
Relative Intensity
734.18289
736.17994
735.18625
738.17699
739.18035
740.17404
741.17740
742.17109
743.17445

Exact mass Compound **20b**

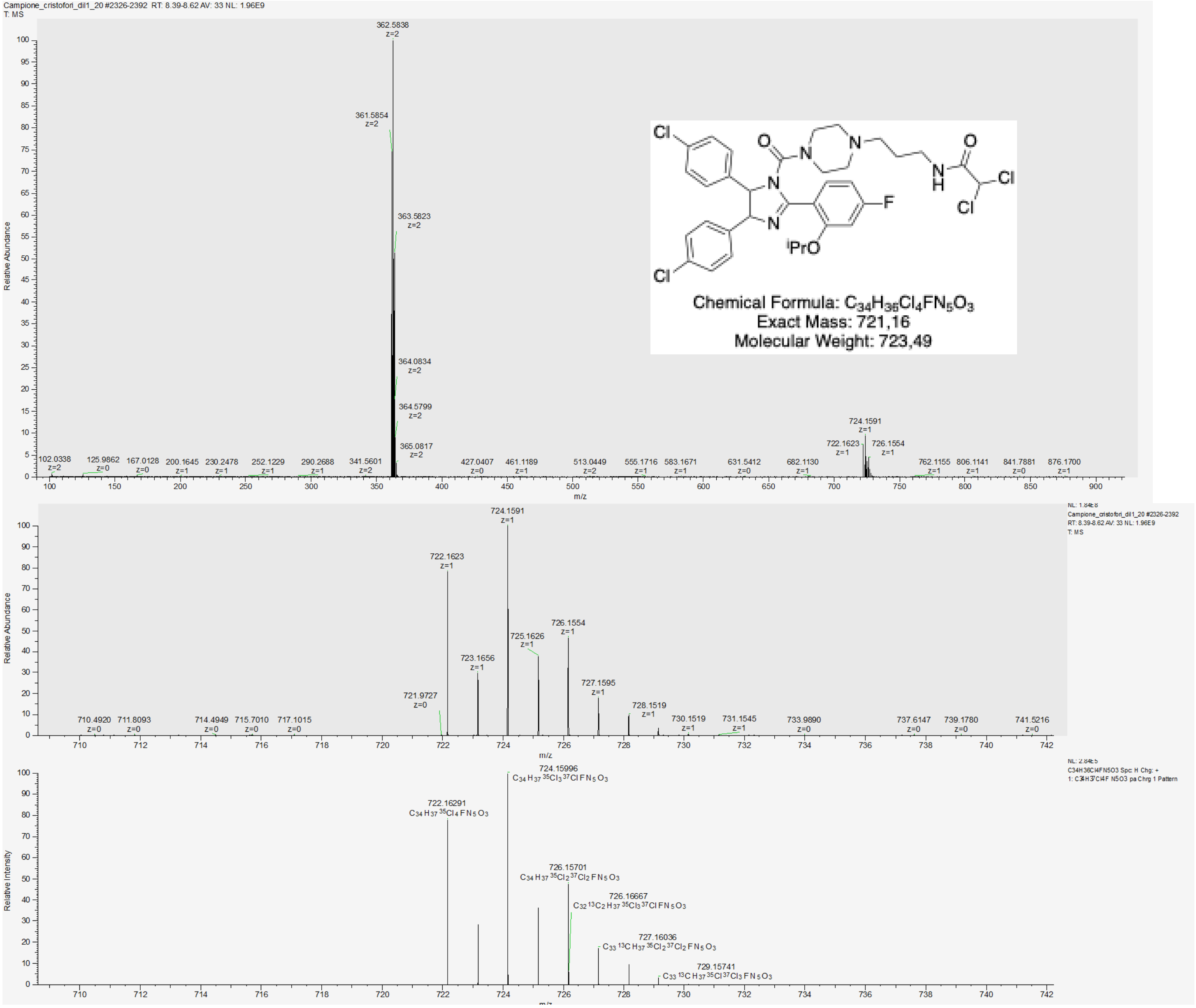

Exact mass Compound 22

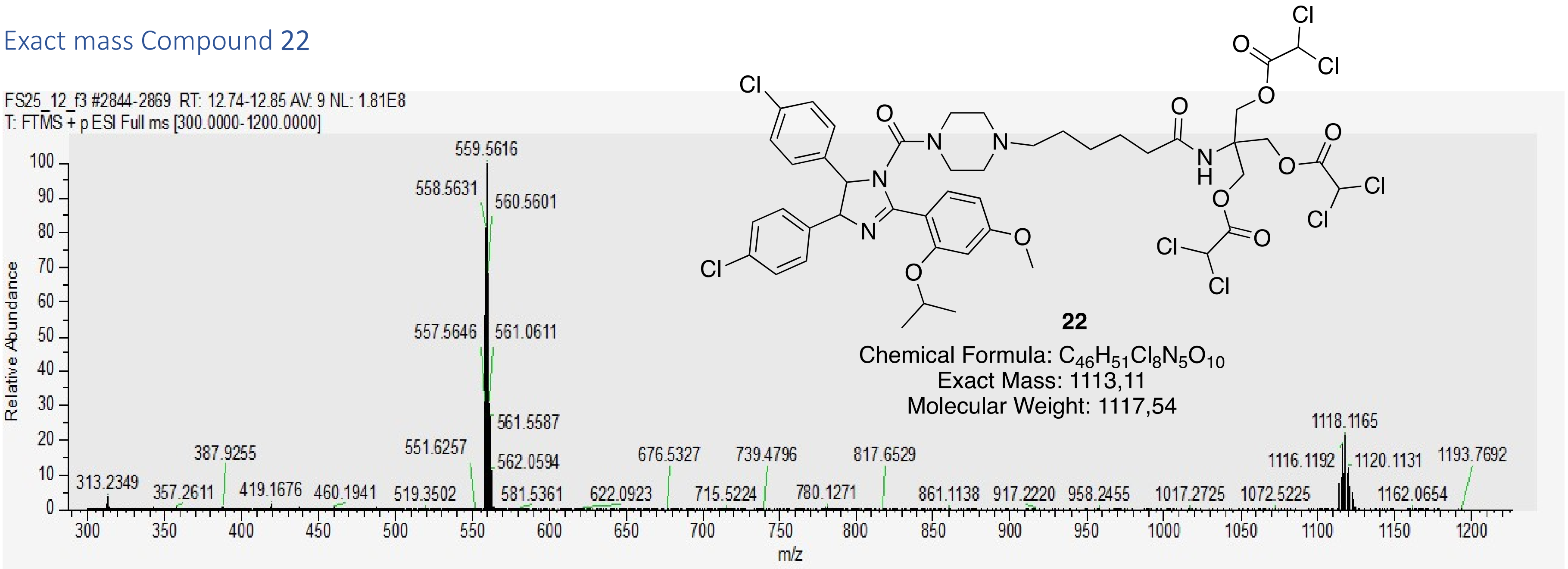

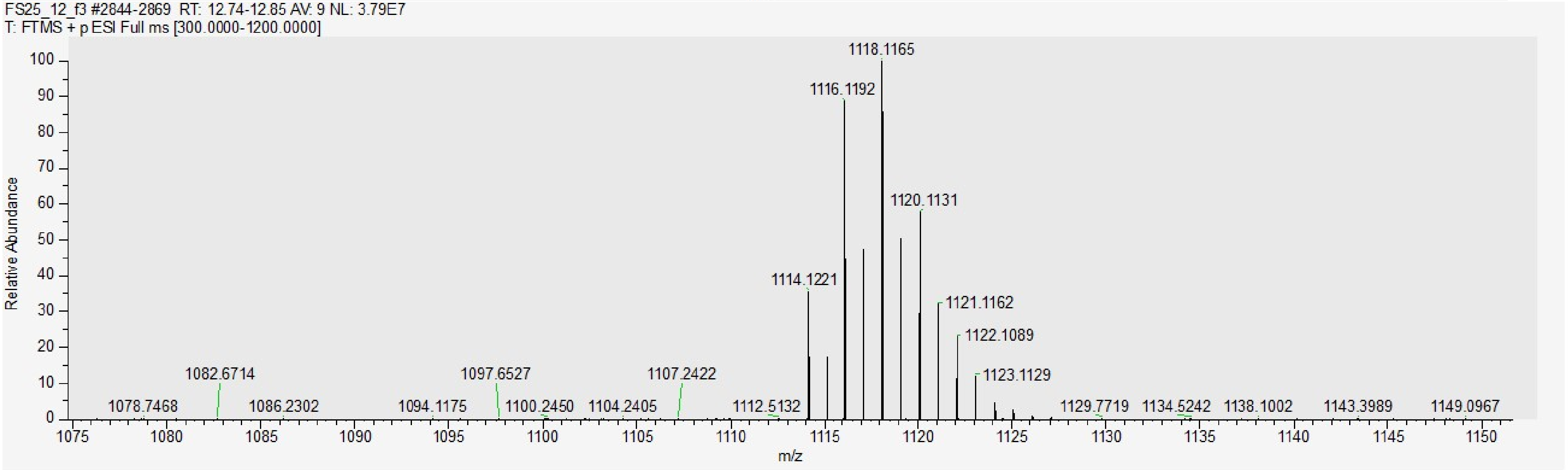

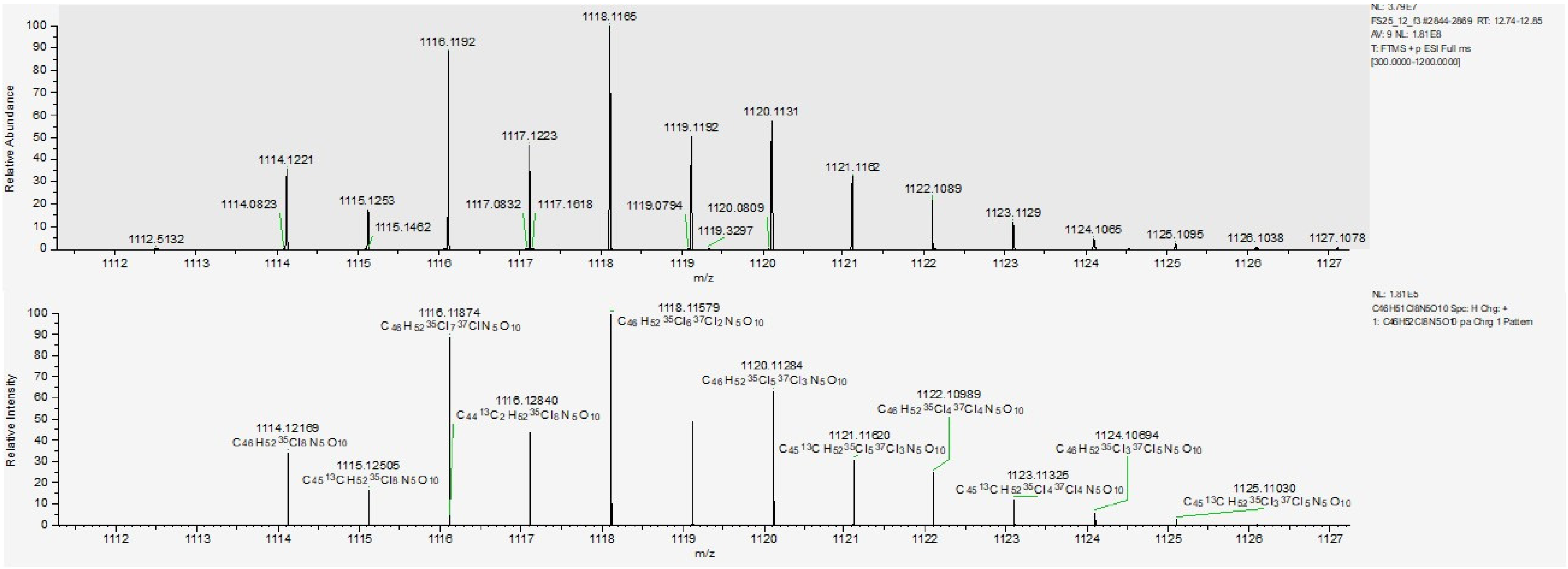
NL: 3.79E7
FS25_12_f3 #2844-2869 RT: 12.74-12.85
AV: 9 NL: 1.81E8
T: FTMS + p ESI Full ms
[300.0000-1200.0000]
Relative Abundance
m/z
NL: 1.81E5
C46H51Cl8N5O10 Spc: H Chg: +
1: C46H52Cl8N5O10 pa Chrg 1 Pattern
Relative Intensity
m/z

Exact mass Compound 28

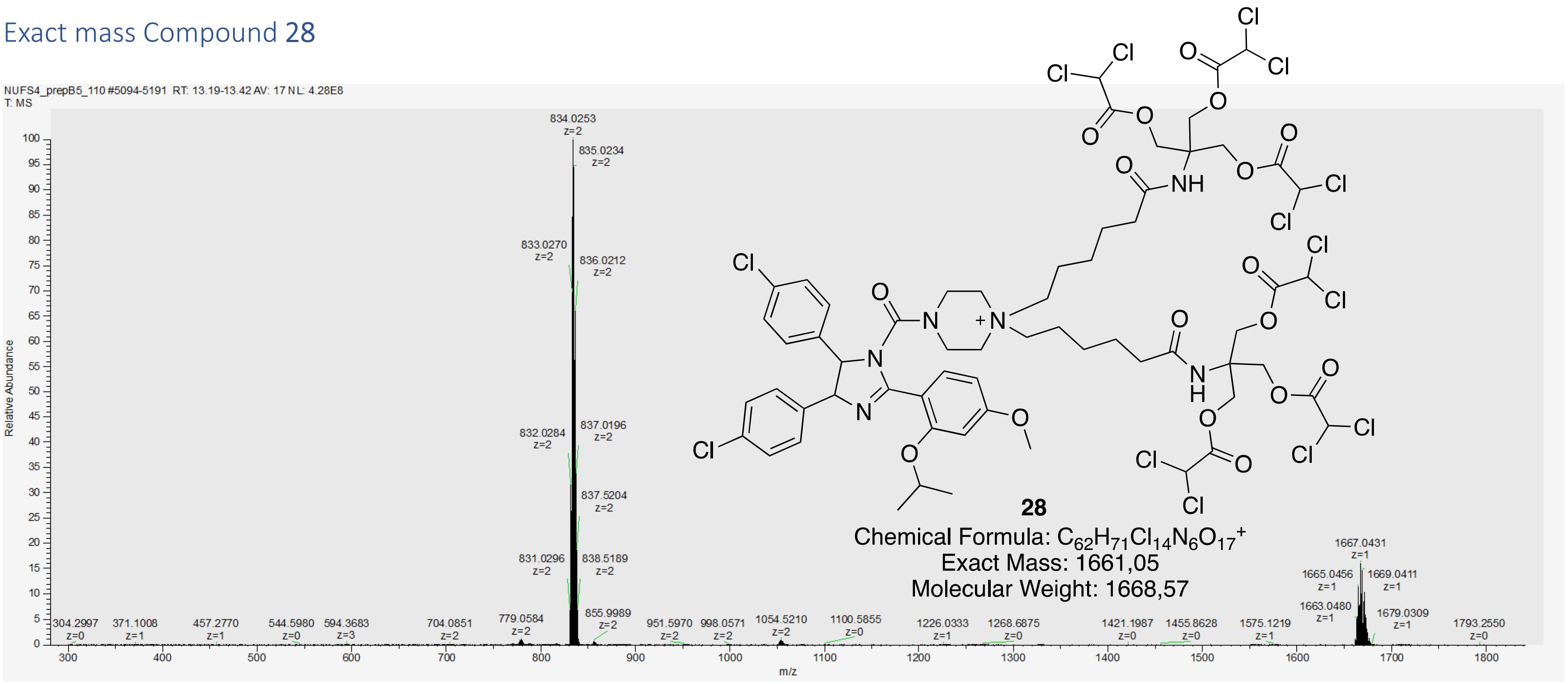

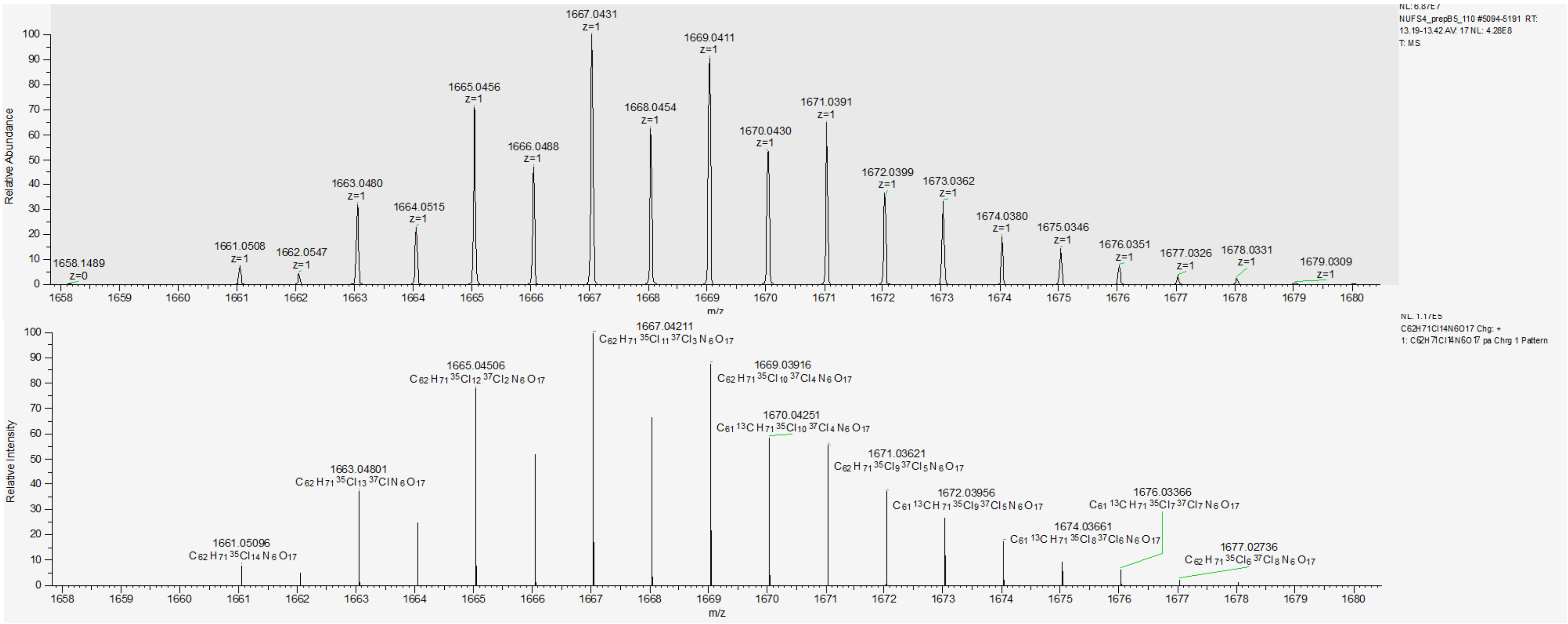
NL: 6.87E7
NUFS4_prepB5_110 #5094-5191 RT: 13.19-13.42 AV: 17 NL: 4.28E8
T: MS
Relative Abundance
1658.1489 z=0
1661.0508 z=1
1662.0547 z=1
1663.0480 z=1
1664.0515 z=1
1665.0456 z=1
1666.0488 z=1
1667.0431 z=1
1668.0454 z=1
1669.0411 z=1
1670.0430 z=1
1671.0391 z=1
1672.0399 z=1
1673.0362 z=1
1674.0380 z=1
1675.0346 z=1
1676.0351 z=1
1677.0326 z=1
1678.0331 z=1
1679.0309 z=1
m/z
NL: 1.17E5
C62H71Cl14N6O17 Chg: +
1: C62H71Cl14N6O17 pa Chrg 1 Pattern
Relative Intensity
1661.05096 C62 H71 35Cl14 N6 O17
1663.04801 C62 H71 35Cl13 37Cl N6 O17
1665.04506 C62 H71 35Cl12 37Cl2 N6 O17
1667.04211 C62 H71 35Cl11 37Cl3 N6 O17
1669.03916 C62 H71 35Cl10 37Cl4 N6 O17
1670.04251 C61 13C H71 35Cl10 37Cl4 N6 O17
1671.03621 C62 H71 35Cl9 37Cl5 N6 O17
1672.03956 C61 13C H71 35Cl9 37Cl5 N6 O17
1674.03661 C61 13C H71 35Cl8 37Cl6 N6 O17
1676.03366 C61 13C H71 35Cl7 37Cl7 N6 O17
1677.02736 C62 H71 35Cl8 37Cl8 N6 O17
m/z

Chiral HPLC Chromatogram Compound 19a

***rac*-19a**	2 mg/ml in 50:50 HEX-IPA
**19a**	1 mg/ml in 100% IPA
***rac*-20a**	1 mg/ml in 100% IPA,
**20a**	1 mg/ml in 100% IPA
Column	WHELK01 150x4.6mm
MPA	H_2O
MPB	ACN
Fv	1ml/min
λ	300 nm
V inj	2µl

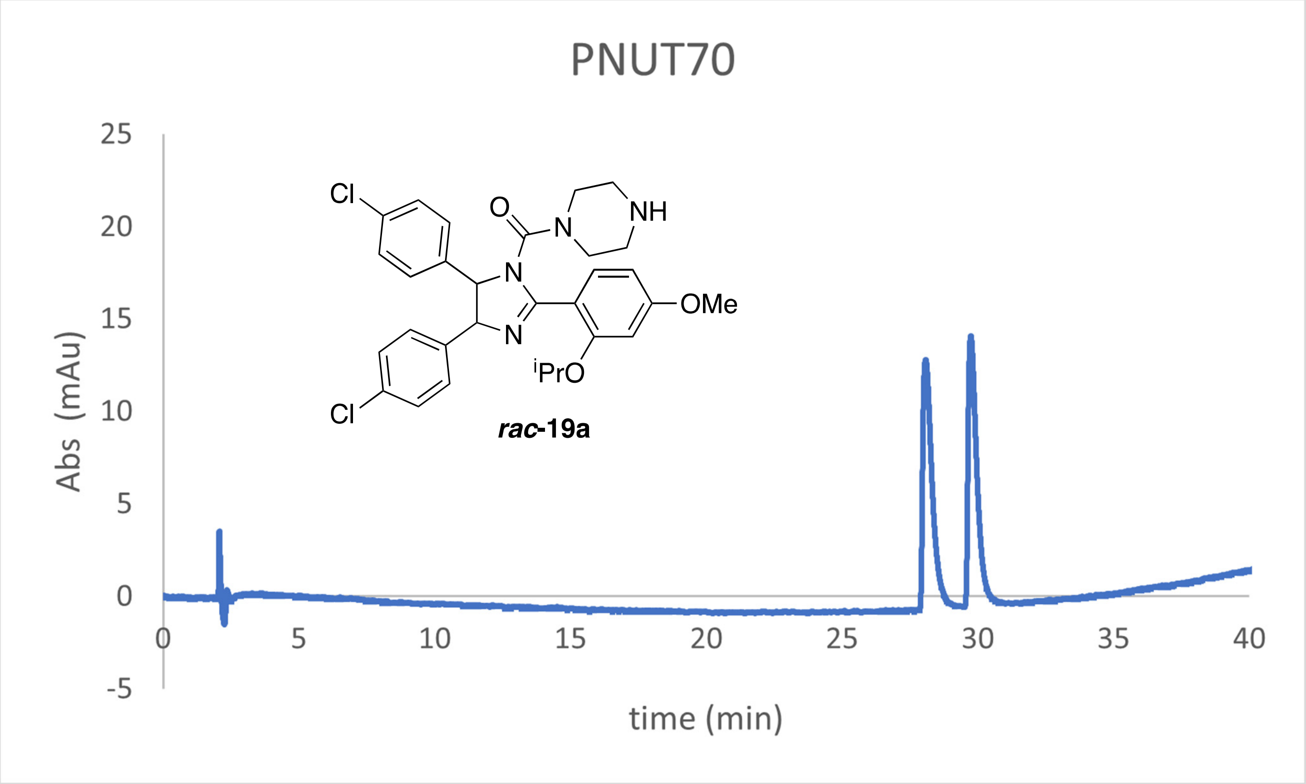

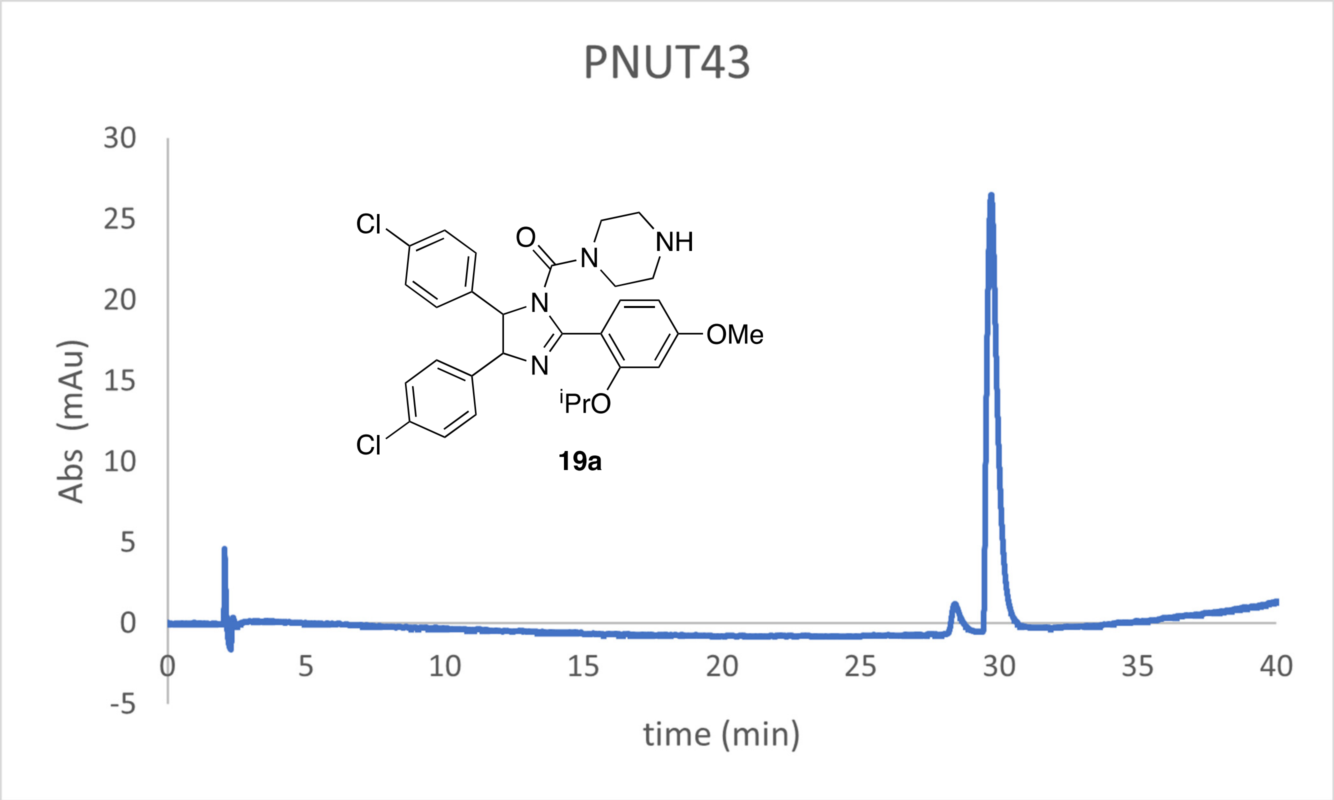

Analytical HPLC (Whelk01 150 mm x 4.6 mm, 5µm. %MP solvent system A= H_2O, B = ACN: from 20% to 40% of B in 40 min. Flow = 1 mL/min. UV: 300 nm): t_R[(1S,2R)-**19a**] = 28.2 min (minor ent), t_R[(1R,2S)-**19a**] = 29.8 min (major ent) ($ee_{\%}$ = 89%).

Chiral HPLC Chromatogram Compound **20a**

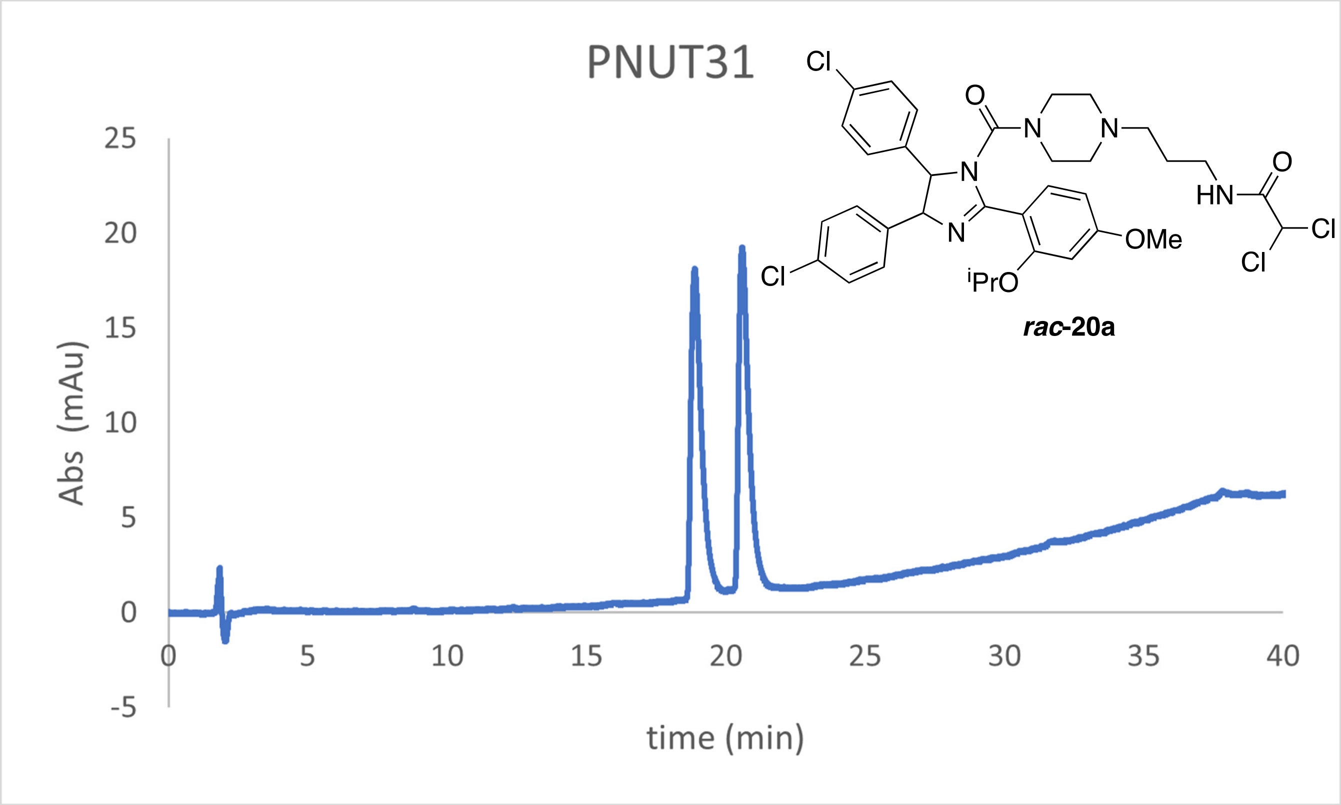

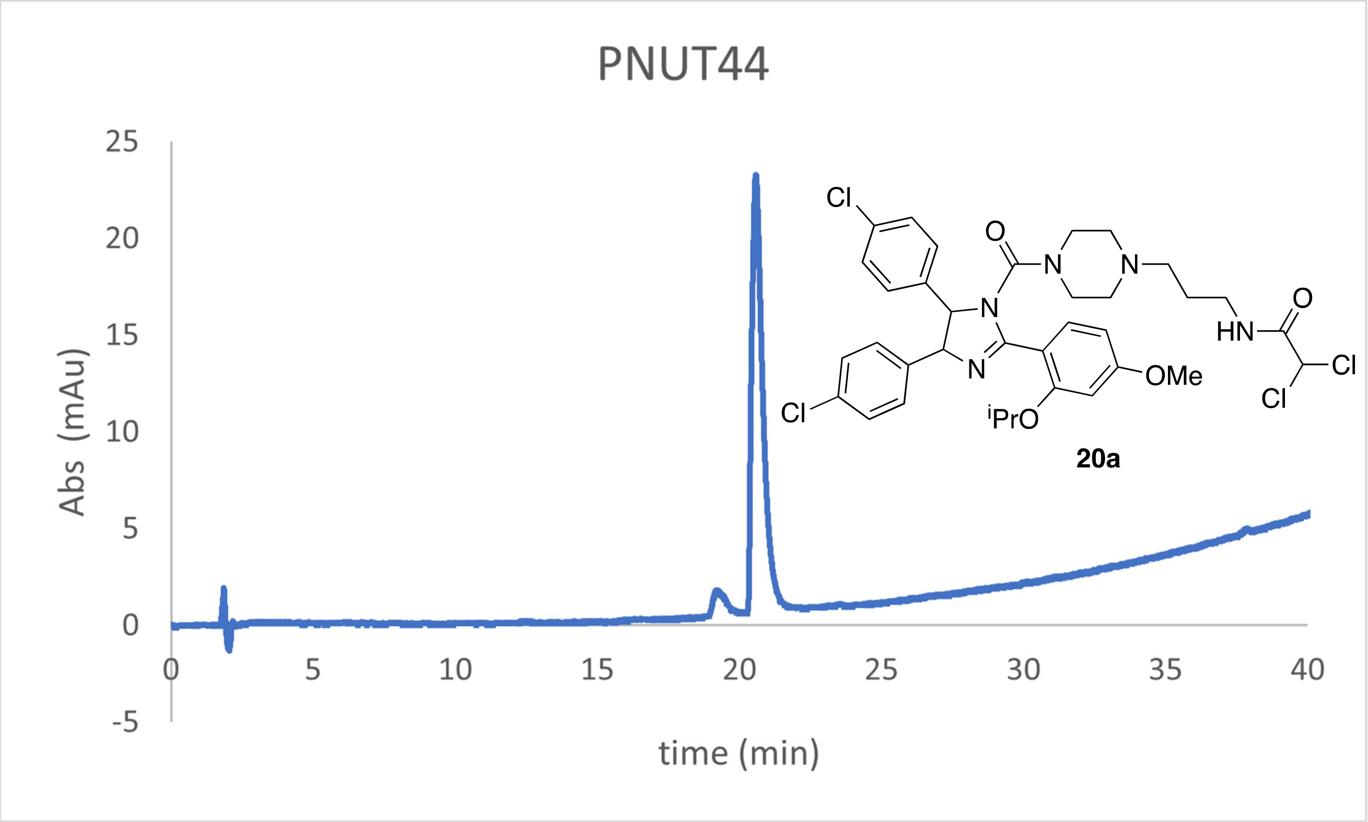

Analytical HPLC (Whelk01 150 mm x 4.6 mm, 5µm. %MP solvent system A= H_2O, B = ACN: from 30% to 50% of B in 40 min. Flow = 1 mL/min. UV: 300 nm): t_R[(4S,5R)-**20a**] = 19.1 min (minor ent), t_R[(4R,5S)-**20a**] = 20.6 min (major ent)($ee_{\%}$ = 88.2%).

Biological results

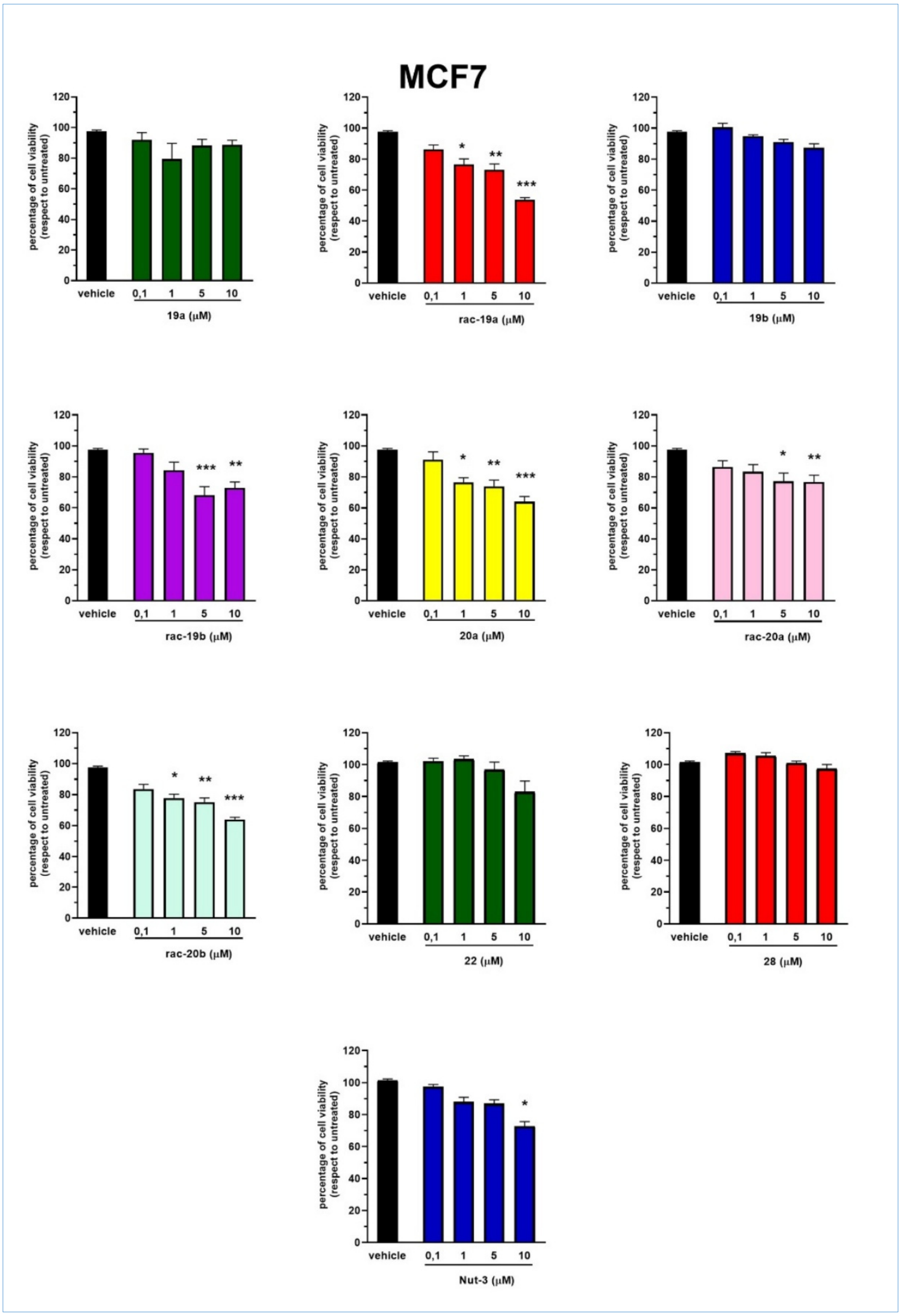

HCT-116^{WT}

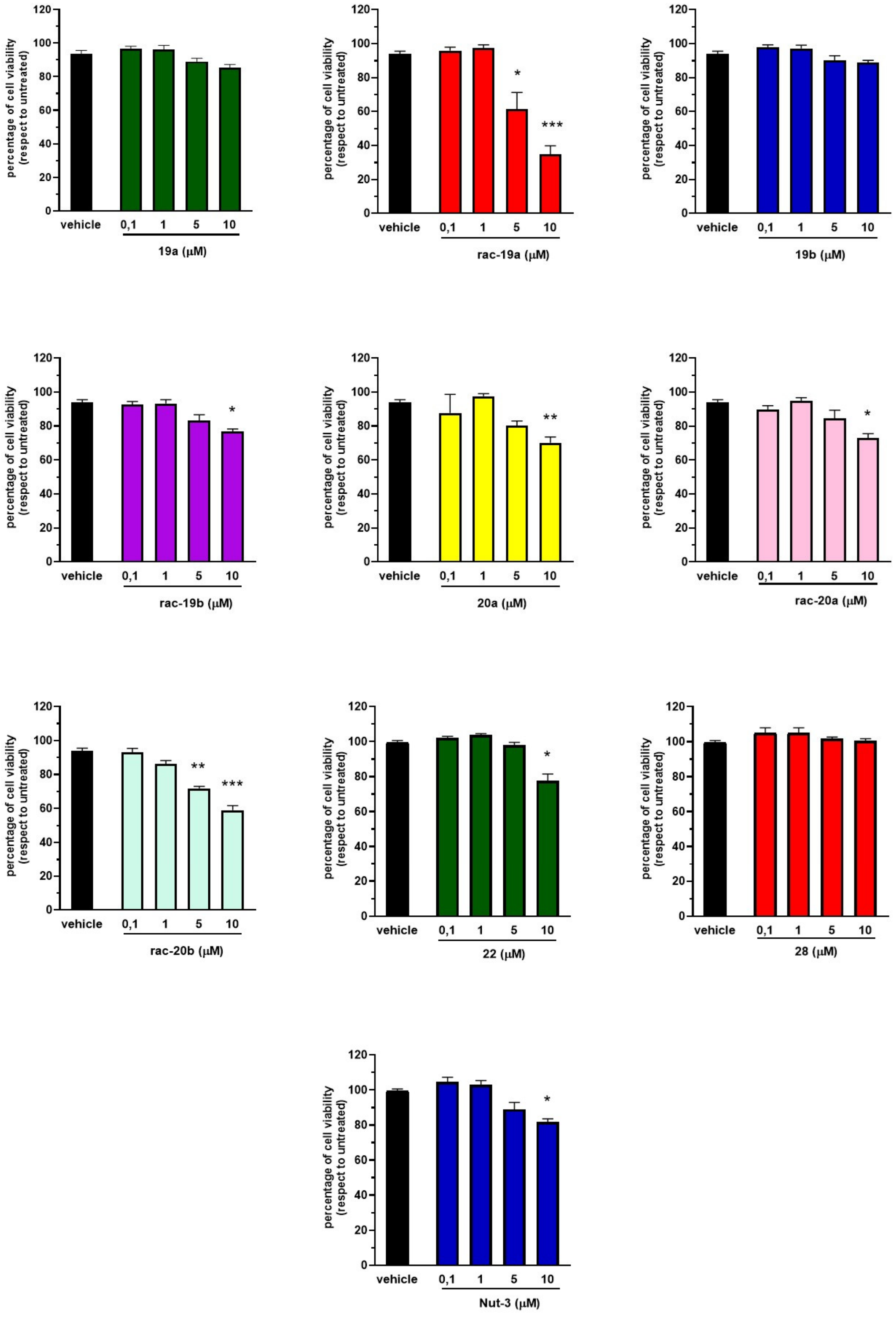
percentage of cell viability (respect to untreated)
120
100
80
60
40
20
0
vehicle
0,1
1
5
10
19a (µM)
rac-19a (µM)
19b (µM)
rac-19b (µM)
20a (µM)
rac-20a (µM)
rac-20b (µM)
22 (µM)
28 (µM)
Nut-3 (µM)
*
**
***

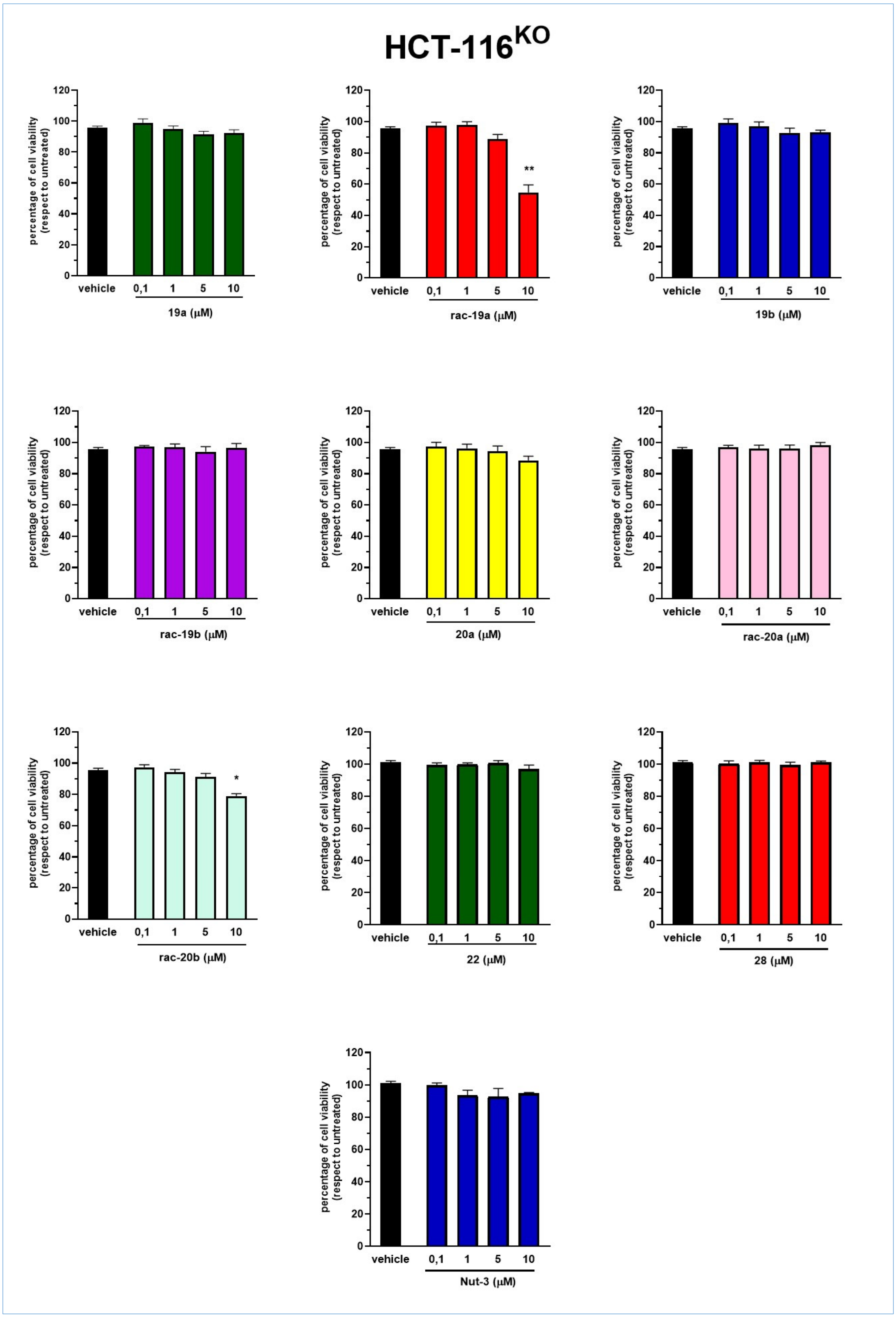

*Cytotoxicity effects of the new synthetic compounds on MCF7 and HCT-116^{WT} cell lines, both expressing p53 wild-type, and on the p53 knock-out HCT-116^{KO} cell line. Cells were treated with the new synthetic compounds (0.1, 1, 5 and 10µM) and commercial Nutlin-3 (Nut-3, 10µM) for 24 hours and assessed with MTT for cytotoxic evaluation. Untreated cultures and cultures exposed to vehicle were run as negative controls. Results are reported as mean±SEM percentage of cell viability respect to untreated cultures set to 100 percent, from at least three independent experiments. Statistical analyses were performed using Kruskal-Wallis method. *p<0.05; **p<0.01; ***p<0.005 respect to vehicle.*
